# Effect of *Xylopia frutescens* Essential Oil on the Activation of Defense Mechanisms Against Phytopathogenic Fungi

**DOI:** 10.3390/microorganisms13112571

**Published:** 2025-11-11

**Authors:** Dalmarcia de Souza C. Mourão, Bruna L. Dias, Mateus S. Dalcin, Luis O. Viteri, Manuel A. Gonzales, Paulo R. S. Fernandes, Vitória B. Silva, Mariana A. Costa, Maria J. González, Ana G. Amaral, Ildon R. do Nascimento, Cristiano B. de Moraes, Vânia Thais S. Gomes, Marcos P. Câmara, Marcos G. da Silva, Adalberto C. Café-Filho, Wellington S. Moura, Gil R. dos Santos

**Affiliations:** 1 Departamento de Fitopatologia, Universidade Federal do Tocantins, Gurupi 77402-970, TO, Brazil; dalmarciaadm@uft.edu.br (D.d.S.C.M.); bruletdias@hotmail.com (B.L.D.); mateussuntidalcin@hotmail.com (M.S.D.); luis.viteri@uft.edu.br (L.O.V.); manuelgon51295@gmail.com (M.A.G.); pauloricardosena@mail.uft.edu.br (P.R.S.F.); vitoria.beatriz@mail.uft.edu.br (V.B.S.); marianna123aguiar@gmail.com (M.A.C.); vania.gomes@ufac.br (V.T.S.G.); 2 Programa de Pós-Graduação em Produção Vegetal, Universidade Federal do Tocantins, Gurupi 77402-970, TO, Brazil; ildon@uft.edu.br; 3 Programa de Pós-Graduação em Biotecnologia, Universidade Federal do Tocantins, Gurupi 77402-970, TO, Brazil; gontijobio@mail.uft.edu.br; 4 Departamento de Bioquímica e Imunologia, Universidade Federal de Minas Gerais, Belo Horizonte 31270-901, MG, Brazil; majito130692@ufmg.br; 5 Departamento de Agronomia, Universidade Federal Rural de Pernambuco, Recife 52171-900, PE, Brazil; gabriele.160713@gmail.com (A.G.A.); marcos.camara@ufrpe.br (M.P.C.); 6 Programa de Pós-Graduação em Ciências Florestais e Ambientais, Universidade Federal do Tocantins, Gurupi 77402-970, TO, Brazil; cbmoraes@mail.uft.edu.br; 7 Departamento de Fitopatologia, Universidade de Brasília, Brasília 70910-900, DF, Brazil; cafefilh@unb.br; 8 Programa de Pós-Graduação em Biodiversidade e Biotecnologia—Rede Bionorte, Universidade Federal do Tocantins, Gurupi 77402-970, TO, Brazil; bussund@gmail.com

**Keywords:** enzyme activity, oxidative stress, resistance induction, *Curvularia lunata*, *Rhizoctonia solani* chitinase, secondary metabolites, *Vigna unguiculata*, *Zea mays*

## Abstract

The induction of resistance in plants involves prior activation of physiological and biochemical systems in the face of external stimuli, promoting greater tolerance to biotic stresses. Faced with the growing challenge of emerging diseases in agricultural plants and the search for more sustainable phytosanitary practices, natural substances are promising alternatives. *Xylopia frutescens*, known as “pindaiba-da-folha-pequena”, native to the Brazilian Cerrado and traditionally used as an antimicrobial treatment, is still little-explored in the literature and presents potentially effective compounds for the control of plant diseases. This study characterized the chemical composition and thermal stability of the *X. frutescens* essential oil (XEO), while evaluating its physiological and phytotoxic effects and the potential for disease control in maize and cowpea plants. The main constituents found in *X. frutescens* essential oil were nopinone (13.75%), spatulenol (12.94%), myrtenal (12.47%), and β-pinene (11.02%). Thermogravimetric analysis indicated that *X. frutescens* is highly volatile, with a large mass loss at 94.74 °C. In bioassays, the oil preserved chlorophyll levels at adequate amounts and activated several antioxidant mechanisms, but also showed a dose-dependent phytotoxic effect. In vitro assays further confirmed its antifungal activity against key phytopathogens, supporting its potential use in disease control. A general increase in the activities of the enzymes superoxide dismutase (SOD), ascorbate peroxidase (APx) and—partially—chitinase (CHIT) was observed. Catalase (CAT) decreased in both maize and cowpea plants treated with this essential oil but was higher in untreated infected plants. Such enzymatic changes suggest that the oil acts as a natural elicitor of resistance, strengthening biochemical and physiological defenses. Finally, disease severities, as measured by AUDPCs, demonstrated significant reductions in the progress of maize “Curvularia leaf spot” (*Curvularia lunata*) and cowpea “Web blight” (*Rhizoctonia solani*). The results highlight the potential of *X. frutescens* essential oil as an active compound stimulating defense mechanisms for applications in sustainable agricultural systems.

## 1. Introduction

The interaction between plants and microorganisms is a complex and dynamic process, in which most pathogens encounter barriers to the establishment of infection. However, when these defense mechanisms fail or are overcome by the pathogens, this leads to the development of diseases that significantly affect global agricultural productivity [1,2]. This constant threat and the growing environmental concerns about conventional agricultural disease control practices require the search for alternative sustainable solutions for phytosanitary management.

In the maize (*Zea mays*) crop, incidence of “Curvularia leaf spot”, caused by the fungus *Curvularia lunata*, increasingly impacts production. The reduction in the leaf photosynthetic area and the impairment of grain formation result in yield losses [3,4]. Similarly, this is observed in cowpea (*Vigna unguiculata*), a crop of great social and economic significance to small farmers [5,6]. Especially in the tropics, cowpea is severely affected by “web blight”, caused by the fungus *Rhizoctonia solani*. *R. solani* causes damping-off, seedling death, leaf necrosis, adult plant death and drastically compromises productivity [7,8,9]. The challenges in the management of these diseases, including possible emergence of resistance to synthetic fungicides and concerns about the environmental impact of fungicide use, drive the need for better and effective alternatives.

As the ability of microorganisms to establish in the host is limited by the plant response, the preventive induction of defense mechanisms may enhance practical plant resistance [10]. This plant immune response, important in restraining pathogenic multiplication, involves the production of reactive oxygen species (ROS) as a central component [11,12]. Although ROS, when generated in excess during oxidative stress, may adversely affect vital molecules [13], plant cells are equipped with enzymatic mechanisms, such as superoxide dismutase (SOD), ascorbate peroxidase (APX), and catalase (CAT), which act to eliminate or reduce these harmful effects while maintaining oxidative balance [14].

Given the general need for better and more sustainable choices for disease management, alternative strategies, including the use of natural substances, have gained prominence. Recent studies have demonstrated that compound of essential oils are capable of activating the plant’s defense mechanisms and are part of the strategies to restrict the activity of the pathogen in plant tissues [15,16,17,18,19,20,21].

Among natural substances, essential oils stand out for their proven antimicrobial action, attributed to active compounds derived from the plant secondary metabolism [22]. Additionally, their limited environmental impact and lower toxicity to humans make them suitable alternatives for phytosanitary control [23,24]. *Xylopia frutescens* Aubl. (Pindaíba-da-folha-pequena), a species native to the Brazilian Cerrado [25], has shown promise for the control of plant diseases. The main compounds reported in this species are pinocarveol, (-)-spathulenol, myrtenal, β-pinene E-caryophyllene, germacrene D, bicyclogermacrene, and α-pinene, which have shown biological activity [26,27,28,29]. Despite the still limited number of studies, antimicrobial, insecticidal, and antifungal activities have been reported for *X. frutescens* essential oil [26,30,31,32], indicating a spectrum of biological activities relevant to disease management. However, the potential effects and mechanisms of XEO related specifically to plant protection are not known, representing a knowledge gap.

In spite of their promising prospects, the stability of essential oils is a challenging factor for practical application, since they are susceptible to degradation by abiotic factors such as light, oxygen, heat, and humidity, which hinders their potential use. Thus, quality-control methods such as thermogravimetric analysis (TGA) are essential for providing information on the thermal stability of these compounds, a prerequisite for the development of effective plant protection products [33,34,35].

The present study reports on the chemical composition and thermal stability of the essential oil of *X. frutescens*, as well as an estimation of the plant enzyme responses induced in XEO-treated maize and cowpea plants [36]. In addition, in vitro assays were conducted to assess the antifungal activity of XEO against phytopathogens. Furthermore, the study includes in planta evaluation of its disease-suppressing effects in fungal pathosystems of two plant host species. These will provide rational bases for understanding the potential of XEO as a sustainable alternative for plant disease management.

## 2. Materials and Methods

### 2.1. Collection, Botanical Provenance and Traceability, and Location of Experiments

The plant collections of *Xylopia frutescens* Aubl. they were carried out in Cerrado areas, located near the municipality of Duere Brazil (11°20′38″ S and 49°16′14″ W, at an altitude of 235 meters) in the winter of 2022. The species collection was registered at the Herbarium of the State University of Tocantins, located at UNITINS Agro. The collection, which was made by Dalmarcia de Sousa Carlos Mourão (Mourão, D.S.C.), received the registration number 7795 (MS2). The experiments were conducted in the laboratories of Phytopathology, Integrated Plant Management and Greenhouse of the Federal University of Tocantins (UFT), Gurupi campus, State of Tocantins, Brazil.

### 2.2. Obtaining the Essential Oil Xylopia frutescens Aubl.

The collected leaves were dehydrated in a greenhouse (approximately 38 °C) for five days. After dehydration, they were cut into small pieces. For the extraction of the essential oil, the method of hydrodistillation in a Clevenger-type apparatus was used. Approximately 200 g of the plant material in two liters of water was subjected to boiling for a period of two hours. After the time elapsed, the essential oil was collected in the form of supernatant with the aid of a micropipette, deposited in amber bottles, and stored in a refrigerator at 4 °C until the moment of implementation of the tests [37].

The essential oil content (EOC) was determined based on the volume/weight (*v*/*w*) of the dry plant material and expressed as a percentage (%), using the following equation:

(1) $$\mathrm{EOC}\;(\%)=\frac{Volume\;of\;extracted\;essential\;oil\left(\mathrm{mL}\right)}{Mass\;of\;dried\;plant\;material\left(g\right)}\times 100$$

### 2.3. Effects on Crops and Diseases

#### 2.3.1. Chromatographic Analysis of Essential Oil *Xylopia frutescens* Aubl.

The qualitative and quantitative analyses of the essential oil were carried out using gas chromatography coupled with mass spectrometry (GC-MS) on a Shimadzu QP2010 Ultra system (Kyoto, Japan). Separation was achieved on a BP-5 capillary column (30 m × 0.25 mm i.d.; 0.25 μm film thickness). Helium was used as the carrier gas at a constant flow rate of 1.0 mL/min with a 1:20 split ratio. The oven temperature program was set from 65 to 210 °C at a heating rate of 3 °C/min. The injector temperature was maintained at 230 °C. The mass spectrometer was operated under electron impact ionization (70 eV), with the ion source temperature at 200 °C. Mass spectra were acquired in the range of 40–650 amu. Identification of the constituents was performed by comparing the obtained spectra with the NIST and Wiley libraries, as well as by comparison of calculated retention indices with literature values. Quantification of the compounds was based on relative peak area normalization, and results are expressed as percentages [38].

#### 2.3.2. Thermal Analysis of *Xylopia frutescens* Essential Oil

The thermal stability of the essential oil was evaluated by thermogravimetric analysis (TGA) in a Shimadzu equipment model TGA Q500 (software version V20.13 Build 39). Approximately 4.045 mg of sample was heated from 25 °C to 950 °C at a constant rate of 10 °C min^−1^. The analysis was performed in an open platinum crucible under a controlled atmosphere. Nitrogen was used as the balance purge gas at a flow rate of 40.0 mL min^−1^ and as the sample purge gas at 60.0 mL min^−1^. Air was also listed as a secondary gas in the system configuration. Thermal events were interpreted according to standard criteria for plant-derived materials. The analysis was conducted by a specialized external laboratory, and all parameters reported above were extracted directly from the official technical documentation.

### 2.4. Bioassays

#### 2.4.1. Phytotoxicity

For phytotoxicity tests, maize (Al Bandeirante) and cowpea (BRS-Guariba) cultivars were sown in plastic pots containing a mixture of bovine manure, soil and commercial substrate Plantmax^®^ (Marafon Industria Metal Plástica Ltd., Cascavel, Parana, Brazil), in a ratio of 1:2:1. The experiment was conducted in a completely randomized design, in a factorial scheme with five concentrations of essential oil (1.0; 2.5; 5.0; 7.5; and 10.0 µL mL^−1^), in addition to the control with sterile water added with Tween 80 (20 µg mL^−1^), totaling six treatments, with three repetitions each. After 200 mL way foliar application was performed 20 days after sowing using a manual spray bottle, and the plants were transferred to controlled environmental conditions (27 °C ± 2; 12:12 light/dark; 80% relative humidity). After 24 h, phytotoxicity assessments were performed based on the adapted scale of Freitas et al. [39] and Cogliatti et al. [40], considering the following ranges: 0% (absence of phytotoxicity); 1–25% (mild leaf necrosis or mild chlorosis); 26–50% (moderate necrosis or moderate chlorosis); 51–75% (intense necrosis or marked chlorosis); and 76–100% (wilting and drying of the plant).

#### 2.4.2. Inhibition of Mycelial Growth

Bioassays were conducted in 90 mm Petri dishes using a completely randomized design with three replicates and five evaluation intervals (2, 4, 6, 8, and 10 days). Each dish received 200 µL of solution of essential oils (2.5 µL mL^−1^) diluted in Tween 80 (20 µg mL^−1^), or chemical fungicide (methyl thiophanate) in 20 µg mL^−1^ uniformly distributed over the surface of PDA medium using a Drigalsky loop. A 4 mm PDA disk containing fungal mycelium was placed at the center of each plate. Plates were sealed, labeled, and incubated at 25 °C for ten days.

#### 2.4.3. Disease Control

Based on the results of the phytotoxicity assays, preventive control tests of Curvularia leaf spot and web blight diseases were installed, in order to evaluate disease severity by calculating the area below the disease progress curve (AUDPC). For these tests, only one concentration of the essential oil (2.5 µL mL^−1^) was used, in addition to the controls: sterile water added with Tween 80 (20 µg mL^−1^), the commercial synthetic fungicide methyl thiophanate in field recommended dose (20 µg mL^−1^) and plants inoculated with the respective pathogens (*Curvularia lunata* for maize and *Rhizoctonia solani* for cowpeas), with three repetitions each. Healthy plants previously received 5 mL of essential oil solution, applied with manual spray bottle. After one hour, the maize plants were inoculated with 5 mL of conidia suspension of *C. lunata* (1 × 10^4^ conidia mL^−1^), while cowpea plants were inoculated with mycelium disks of *R. solani* positioned at the base of the stem. After inoculation, all plants were kept in a humid chamber for 48 h to favor the development of pathogens. The severity of the disease was evaluated at five points in time, with intervals of two days between each evaluation, using the scale proposed by Santos et al. [41]: 0 (healthy plant); 1 (less than 1% of affected leaf area); 3 (1 to 5%); 5 (6 to 25%); 7 (26 to 50%); and 9 (more than 50% of compromised leaf area).

In addition, fresh leaf tissue samples were collected 96 h after inoculation, in order to perform biochemical analysis, including the quantification of organic compounds (chlorophyll) and the evaluation of antioxidant enzyme activities: superoxide dismutase (SOD), catalase (CAT), ascorbate peroxidase (APx) and chitinase (CHIT).

### 2.5. Quantification of Organic Compounds

Samples of fresh leaf tissue, sufficient for the determination of organic compounds (chlorophyll a and b, and total chlorophyll), were collected and kept under refrigeration at −20 °C until the time of analysis.

The contents of chlorophyll a (Equation (2)) and b (Equation (3)), and total chlorophyll (Equation (4)), were determined, according to the method proposed by [42]. For pigment extraction, leaf sample fragments were left overnight in darkened tubes containing 80% ethanol. After this period, the samples were filtered, and the supernatant collected was used to determine the absorbance in a spectrophotometer at wavelengths of 663 nm and 647 nm. To quantify chlorophyll a and b, the following calculations were used:

Chlorophyll a = [(12.25 × A_663_)] − (2.79 × A_647_)] × V, (2)

Chlorophyll b = [(21.50 × A_647_) − (5.10 × A_663_)] × V, (3)

Total chlorophylls = [(18.71 × A_647_) − (7.15 × A_663_)] × V, (4)

where A corresponds to the absorbance of the extract at the given wavelength (nm) and V is the final volume of the chlorophyll extract in ethanol (mL). Chlorophyll data were expressed in milligrams per gram of fresh mass (mg g^−1^ MF).

### 2.6. Enzymatic Activities in Maize and Cowpea Plants Exposed to Xylopia frutescens Essential Oil

The determination of enzymatic activity was performed on fresh leaves of maize and cowpeas. Individually, samples of three different plants of each species were prepared by weighing 200 mg of fresh leaves, macerated in liquid nitrogen with 20% polyvinylpyrrolidone (PVPP). When forming the powder, 1.5 mL of extraction buffer (100 mM potassium phosphate buffer; pH 7.0, added 1 mM EDTA and 1 mM ascorbate to the volume of buffer used) was added. Maceration occurred for another 3 min, and the extract was centrifuged at 14,000× *g* for 25 min at 4 ± 1 °C. The supernatant was collected (protein extract) and stored at −20 °C for analysis.

#### 2.6.1. Superoxide Dismutase (SOD)

For SOD activity, inhibition of blue formazan production was determined by photoreduction of NBT (Nitroblue Tetrazolium). SOD was measured by adding in test tubes: 0.1 mL of protein extract; 0.1 mL of 50 mM potassium phosphate buffer, pH 7.8; 0.02 mL of 0.1 mM EDTA; 0.4 mL of 70 mM L-methionine and 0.2 mL of 1 mM NBT. The reaction was initiated by the addition of 2 mM riboflavin and rapid transfer of the tubes, with light protection, to a chamber illuminated by a 30-watt lamp (30 µmol of photons m^−2^ s^−1^), for 5 min. Absorbance was measured at 540 nm (BioSpectro model SP-220) [43]. One unit of SOD activity was defined as the amount of enzyme required to inhibit 50% of NBT reduction, and the activity was expressed in unit per gram of mass per extract per minute (U g^−1^ E^−1^ min^−1^), according to Beauchamp and Fridovich [44].

#### 2.6.2. Catalase (CAT)

To determine the activity of CAT, in a test tube, 0.05 mL of protein extract was added; 2.95 mL of 50 mM potassium phosphate buffer, pH 7.8, added 20 mM hydrogen peroxide. The activity was measured at 240 nm (BioSpectro model SP-220) for 300 seconds, with readings taken every 30 seconds, verifying the absorbance decay at each reading. For determination of the unit, the readings were calculated based on the molar extinction coefficient of 35 M^−1^ cm^−1^ (240 nm). The activity was expressed in micromol of hydrogen peroxide per gram of extract per minute (µmol H_2_O_2_ g^−1^ E^−1^ min^−1^) [45].

#### 2.6.3. Ascorbate Peroxidase (APX)

APX enzyme activity was performed by mixing 0.1 mL of protein extract, 2.7 mL of 0.5 mM ascorbate buffer (ASA) and 0.2 mL of hydrogen peroxide at 30 mM. Absorbance was measured at 290 nm (BioSpectro model SP-220), at 25 °C, by hydrogen peroxide degradation. APX activity was expressed in micromol of ascorbate per gram of extract per minute (µmol ASA g^−1^ E^−1^ min^−1^) [46].

#### 2.6.4. Chitinase (CHIT)

To determine the activity of CHIT, the release of soluble fragments of “CM-chitin-RBV^®^” (Sigma-Aldrich, St. Louis, MO, USA) was observed using carboxymethylated chitin labeled with bright violet remazol. For the reaction, 0.2 mL of the protein extract, 0.6 mL of the 0.1 M sodium acetate buffer (pH 5.0) and 0.2 mL of “CM-chitin-RBV^®^” at 2.0 mg mL were added^−1^. Subsequently, the samples were incubated at 40 °C for 20 min. The reactions were stopped with the addition of 0.2 mL of 1 M HCl, cooled on ice and centrifuged at 10,000× *g* for 5 min. The supernatants of the reactions were read at 550 nm in a spectrophotometer (BioSpectro model SP-220). The activity of the CHIT enzyme was expressed in absorbance units per minute (U min^−1^) [47].

### 2.7. Statistical Analysis

The data of response to increasing concentration of essential oil were analyzed by regression analysis with adjustment of the curve that best represented the values. Values of the same variable response when exposed to different treatment were subjected to One-Way ANOVA (*p* < 0.05) using the GraphPad Prism Software, version 8.1. The normality (Shapiro–Wilk) and homogeneity of variance was checked and did not need value transformation.

## 3. Results

### 3.1. Yield and Characterization of the Essential Oil

The Essential Oil Content (EOC), determined via hydrodistillation from the dried material, was 0.10% *±* 0.01 *(v*/*w*). This percentage corresponds to an average yield of 0.200 mL of essential oil per 200 g of plant material. The measured density of the oil was 0.9865 g/mL at 25 °C.

#### 3.1.1. Chromatography of *Xylopia frutescens* Essential Oil

The analysis of essential oil from the leaves of *X. frutescens* identified a total of 20 chemical constituents (Table 1). With four compounds representing the 50.2%; being pinocarveol/isopinocarveol (13.8%), (-)-spathulenol (12.9%), myrtenal (12.5%) and β-pinene (11.0%) (Table 1). The other constituents as the α-pinene, myrtenol, pinocarvone, verbenol, isospatulenol, germacrene D, verbenone, nopinone, α-phellandrene-8-ol, eucalyptol, α-campholenal, ρ-cymene, thuja-2,4(10)-diene, caryophyllene oxide, δ-elemene, and (-)-spatulenol represented the 49.8% of total (Table 1, Supplementary file S1).

#### 3.1.2. Thermogravimetric Analysis (TGA) of *Xylopia frutescens* Essential Oil

**Figure 1:**
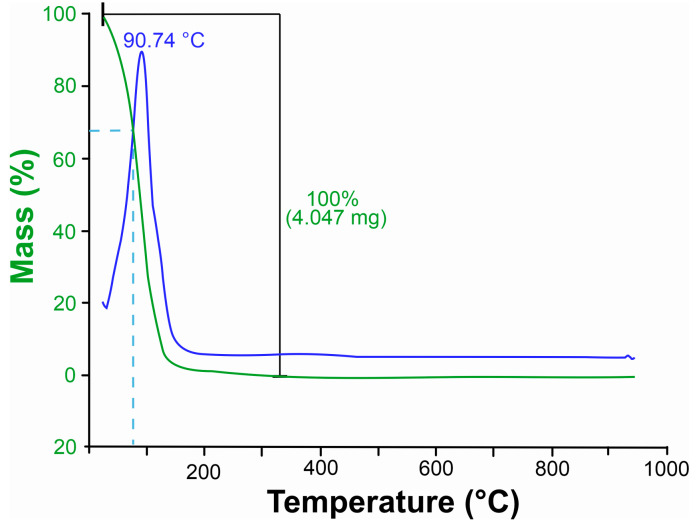
Thermogravimetric curve of leaves essential oil *Xylopia frutescens*. The green curves represent thermal degradation based on weight loss, indicating the mass reduction in the sample as temperature increases. The blue curves correspond to the derivative thermogravimetric (DTG) data, highlighting the rate of weight loss. Peaks in the blue curve indicate the temperatures at which the most significant thermal events occur, such as maximum decomposition or volatilization.

Thermogravimetric analysis (TGA) of *Xylopia frutescens* essential oil revealed mass loss events as a function of temperature increase (Figure 1). Already at room temperature, an initial loss of XEO mass was noted, probably caused by the volatilization of lighter compounds, followed by a sharp decrease at the very beginning of the process. The most significant mass loss occurred at 94 °C, with *c*. 32.5% mass reduction. The main total thermal degradation of the oil happened within the temperature range of 330 and 350 °C.

### 3.2. Mycelial Growth of Curvularia lunata and Rhizoctonia solani

Our results show that in the mean mycelial growth rate in the control treatment for *Curvularia lunata*, which contained only water and Tween, mycelial growth was initially high (12.2 mm/day at 48 h) (Table 2). However, growth progressively declined as the colony approached the edge of the Petri dish, resulting in a final mean mycelial growth rate (MMGR) of 9.0 mm/day. The commercial fungicide exhibited a similar pattern, with peak growth observed on day six (11.4 mm/day), also yielding a final MMGR of 9.0 mm/day. In contrast, the essential oil treatment showed moderate initial growth (6.2 mm/day), followed by a continuous increase, reaching 10.9 mm/day by day ten and a final MMGR of 8.6 mm/day. For *Rhizoctonia solani*, both the control and fungicide treatments induced rapid initial growth (35.8 mm/day on day two), which ceased entirely after day six due to full colonization of the plate surface, resulting in a final MMGR of 9.0 mm/day. The essential oil, however, completely inhibited initial growth (0 mm/day on day two), but this effect was followed by gradual recovery, culminating in 13.2 mm/day on day ten and a final MMGR of 8.5 mm/day.

### 3.3. Phytotoxic and Disease-Suppressing Effects on Maize and Cowpea Plants

#### 3.3.1. Phytotoxicity of *Xylopia frutescens* Essential Oil in Maize and Cowpea Plants

**Figure 2:**
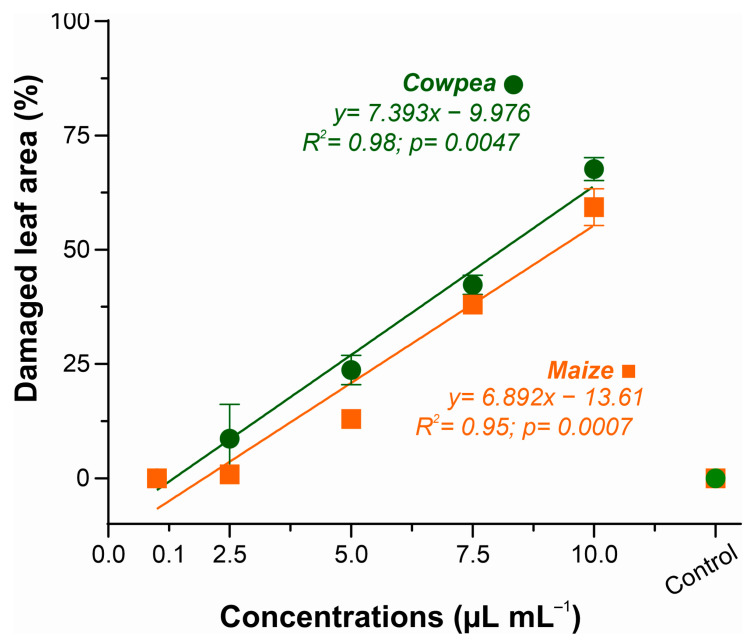
Phytotoxicity of essential oil of *Xylopia frutescens* in maize and cowpea plants after 24 h of treated. Bars show the percentage (mean ± SE) of three replicates about the damaged leaf area as a function of the concentration of essential oil applied.

Figure 2 shows the percentage of damaged leaf area as a function of the increase in XEO concentrations, showing an increasing phytotoxic effect. Cowpea plants showed slightly higher sensitivity than maize plants, with data adjusted to a linear regression model (R^2^ = 0.98), indicating a strong correlation between increased EO concentration and percentage of damaged leaf area.

A similar response was observed in maize (R^2^ = 0.95), with toxicity levels ranging from 0% (control) to 59.8% at the highest concentration (10.0 µL mL^−1^). Both species presented progressive toxic response, with onset of leaf lesions at 2.5 µL mL^−1^. The concentration of 5.0 µL mL^−1^ caused 23.6% leaf necrosis in cowpea and 13% in maize. At the maximum concentration (10.0 µL mL^−1^), leaf damage due to XEO phytotoxicity ranged from 59.8% in maize to 67.6% in cowpeas. These results were employed for the selection of the ideal concentration of the essential oil to be used in subsequent analyses of the disease suppressive effects of XEO.

#### 3.3.2. Disease Estimates by the Area Under the Disease Progress Curve (AUDPC)

**Figure 3:**
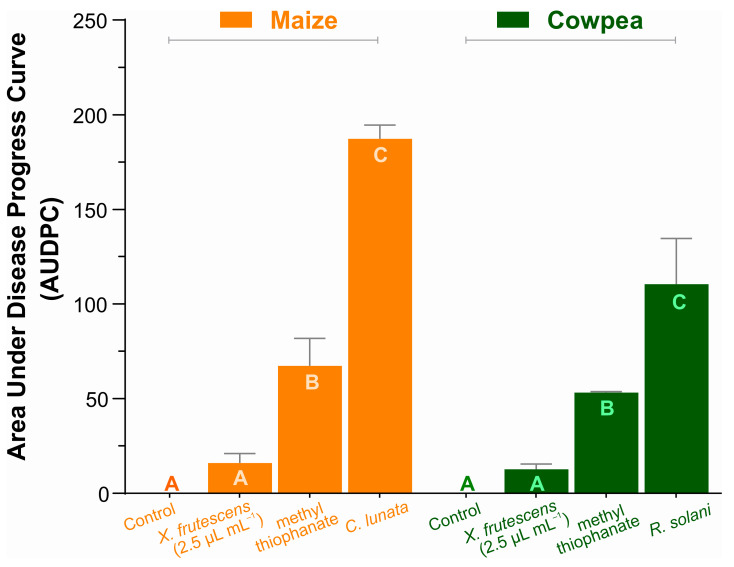
Area under the disease progress curve in maize plants (Curvularia leaf spot), and cowpea (web blight). Bars show the means (±SE) of three replicates; for each plant species, bars with the same letter do not differ significantly according to Tukey’s test (*p* < 0.05).

Two fungal pathosystems were selected for estimating the disease-suppressing effect of XEO in two plant species belonging to the Poaceae and Fabaceae families. Figure 3 demonstrates the XEO potential for the control of maize Curvularia leaf spot and cowpea web blight at the concentration of 2.5 µL mL^−1^, as compared to a commercial fungicide. Disease measurements are represented as the cumulative area under disease progress curves (AUDPC), calculated as described by Shaner and Finney [50].

The AUDPC values in maize and cowpea are not comparable, since they refer to different pathosystems. Still, regardless of the crop or pathogen, the essential oil of *X. frutescens* measurably and significantly reduced severity of both diseases, even more than the commercial fungicide. In fact, AUDPCs of pathogen-inoculated, XEO-treated plants in both the Poaceous and the Fabaceous plant species were so low that they did not differ statistically from the healthy plant (control). Pathogen-inoculated, non-treated plants of each plant species had the larger AUDPCs.

### 3.4. Chlorophyll Content on Healthy and Diseased Maize and Cowpea Plants Exposed to Xylopia frutescens Essential Oil

**Figure 4:**
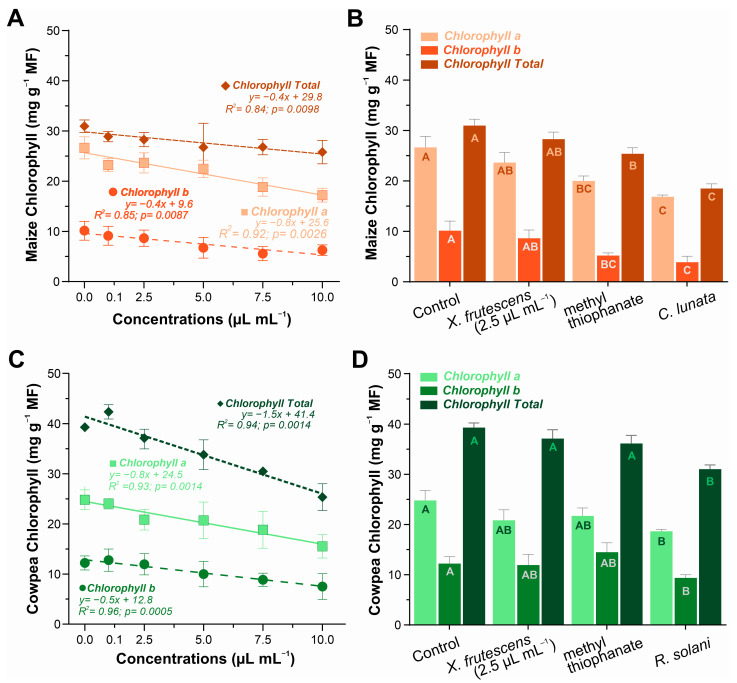
Content of type chlorophyll a, chlorophyll b and chlorophyll total in maize plant (**A**,**B**) and cowpea plants (**C**,**D**) when exposed to different concentrations of *X. frutescens* essential oil (**left**); and response to different treatments, including *X. frutescens* methyl thiophanate and infested plants (**right**). Symbols or bars show the mean (±SE) of three replicates, bar means followed by the same uppercase letters do not differ statistically from each other by the Tukey test (*p* < 0.05).

In maize plants, the contents of chlorophyll a, chlorophyll b, and total chlorophyll showed a smooth linear decrease as the concentration of essential oil increased (Figure 4A). In the comparative analysis involving 2.5 µL mL^−1^ of essential oil and a synthetic fungicide, it was evident that *X. frutescens* did not affect chlorophyll content; however, a reduction was observed in plants treated with methyl thiophanate and those infested with *C. lunata* (Figure 4B). In cowpea plants, a similar linear decreasing trend in chlorophyll content was observed, inversely proportional to the concentration of essential oil, indicating a negative effect at higher concentrations of *X. frutescens* (Figure 4C). When comparing different treatments in cowpea, no significant differences were found, except in plants infested with *R. solani*, where a reduction in chlorophyll a and b, and total chlorophyll was observed (Figure 4D).

### 3.5. Enzymatic Activities in Healthy and Diseased Maize and Cowpea Plants

#### 3.5.1. Superoxide Dismutase (SOD) Expression

**Figure 5:**
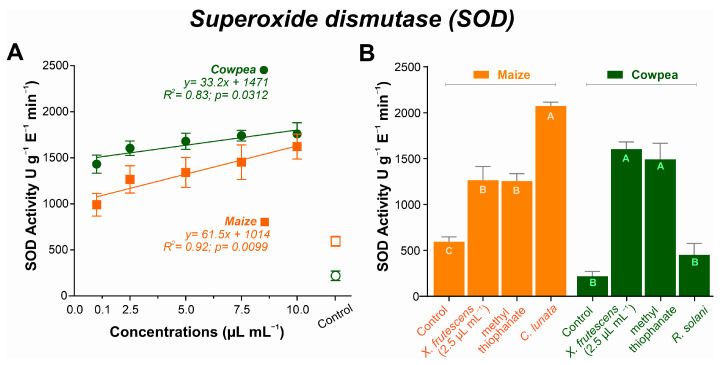
Superoxide dismutase (SOD) activity in maize and cowpea plants exposed to different concentrations of *X. frutescens* essential oil (**A**); and expression of SOD enzyme activity in different treatments (**B**). Symbols and bars show the mean (±SE) of three replicates; bars with the same letter do not differ statistically between treatments of the same plant (Tukey’s test, *p* < 0.05).

In both plant species, SOD activity increased linearly with increasing amounts of the *X. frutescens* essential oil. In maize, SOD activity started to increase at the concentration of 0.1 µL mL^−1^, reaching a maximum of 1621 U g^−1^ E^−1^ min^−1^. A similar response pattern was observed in cowpea plants, ranging from 1431 U g^−1^ E^−1^ min^−1^ at 0.1 µL mL^−1^ up to 1759 U g^−1^ E^−1^ min^−1^ at the highest concentration (Figure 5A). Figure 5B compares SOD activity in healthy plants, or plants inoculated with the respective pathogen, and treated with *X. frutescens* essential oil at 2.5 µL mL^−1^ or a commercial fungicide (methyl thiophanate). In maize, healthy plants presented the lowest SOD activity (594 U g^−1^ E^−1^ min^−1^), while activity increased most in non-treated, diseased plants, reaching 2071 U g^−11^ E^−1^ min^−1^. Diseased maize plants treated with the essential oil or fungicide showed intermediate SOD activities that were not significantly different. In maize plants, treatment with essential oil at 2.5 µL/mL significantly reduced SOD activity compared to the control (mean difference = −671.4; 95% CI: −910.7 to −432.1; *p* < 0.0001). For healthy cowpea plants, SOD activity was the lowest (219 U g^−1^ E^−1^ min^−1^), not significantly different from non-treated, diseased plants (451 U g^−1^ E^−1^ min^−1^). 

#### 3.5.2. Catalase (CAT) Expression

**Figure 6:**
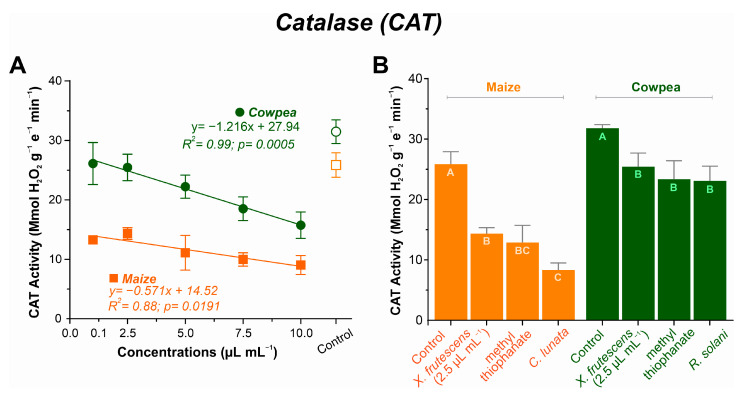
Catalase (CAT) activity in maize and cowpea plants exposed to different concentrations of *X. frutescens* essential oil (**A**); and expression of CAT enzyme activity in different treatments (**B**). Symbols and bars show the mean (±SE) of three replicates; and bars with the same letter do not differ statistically between treatments of the same plant (Tukey test, *p* < 0.05).

As the concentration of the essential oil increased, decreasing linear patterns were observed in both plant species, albeit more pronouncedly in cowpea (Figure 6A). The highest CAT values were recorded in plants treated with 2.5 µL mL^−1^ of the essential oil, at 14.35 µmol H_2_O_2_ g^−1^ E^−1^ min^−1^ for maize and 25.46 µmol H_2_O_2_ g^−1^ E^−1^ min^−1^ for cowpea. Respective minimal values were recorded at 10.0 µL mL^−1^ of the essential oil (9.05 µmol H_2_O_2_ g^−1^ e^−1^ min^−1^ for maize and 15.74 µmol H_2_O_2_ g^−1^ e^−1^ min^−1^ for cowpea).

CAT activity in non-treated healthy plants, non-treated diseased plants or diseased plants treated with *X. frutescens* (at 2.5 µL mL^−1^) or the fungicide is shown in Figure 6B. In maize, CAT activity was the highest in healthy plants (25.85 µmol H_2_O_2_ g^−1^ E^−1^ min^−1^) and lowest in the untreated diseased plants (8.33 µmol H_2_O_2_ g^−1^ E^−1^ min^−1^). Inoculated plants treated with the essential oil or the fungicide showed intermediate CAT activities (14.35 µmol H_2_O_2_ g^−1^ E^−1^ min^−1^ and 12.87 µmol H_2_O_2_ g^−1^ E^−1^ min^−1^ for XEO and the fungicide, respectively), and were not significantly different. Similarly, for cowpea, untreated healthy control plants had the highest CAT activity, whereas diseased plants had their CAT activity significantly depressed.

#### 3.5.3. Ascorbate Peroxidase (APx) Activity

**Figure 7:**
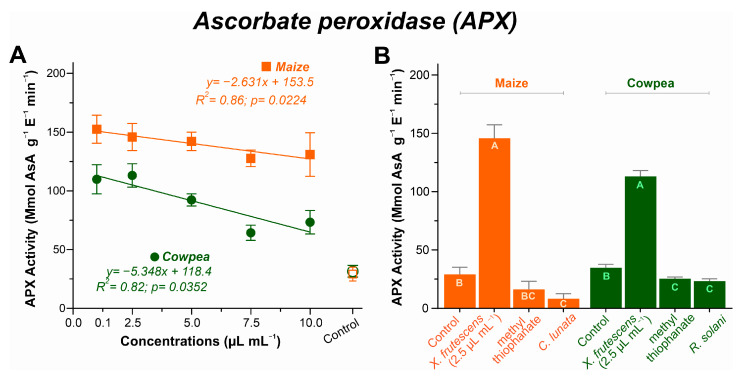
Ascorbate peroxidase (APX) activity in maize and cowpea plants exposed to different concentrations of *X. frutescens* essential oil (**A**); and expression of APX enzyme activity in different treatments (**B**). Symbols and bars show the mean (±SE) of three replicates; and bars with the same letter do not differ statistically between treatments of the same plant (Tukey test, *p* < 0.05).

For both plant species, APx activity decreased linearly as a function of the increase in *X. frutescens* essential oil, with somewhat less pronounced decrease for maize, as depicted in Figure 7A. In maize, the enzyme activity started at 0.1 µL mL^−1^ with 152 µmol ASA g^−1^ E^−1^ min^−1^ and was reduced to 130 µmol ASA g^−1^ E^−1^ min^−1^ at 10 µL mL^−1^. For cowpea, a more pronounced variation in APX activity was observed, ranging from 113.1 µmol ASA g^−1^ E^−1^ min^−1^ at 2.5 µL mL^−1^ of XEO to 73.3 µmol ASA g^−1^ E^−1^ min^−1^ at 10 µL mL^−1^.

The comparison among healthy, diseased and diseased–treated plants is represented in Figure 7B. XEO-treated maize and cowpea plants had remarkably higher APx activities among all treatments (*p* < 0.05). Indeed, the application of 2.5 µL mL^−1^ of XEO caused *c*. 7-fold increase in the enzyme activity in treated maize plants, and *c*. 4-fold increase in treated cowpea plants, as compared to healthy plants. Diseased plants, either untreated or treated with the fungicide, showed the lowest APX activities in both plant species.

#### 3.5.4. Chitinase (CHIT) Activity

**Figure 8:**
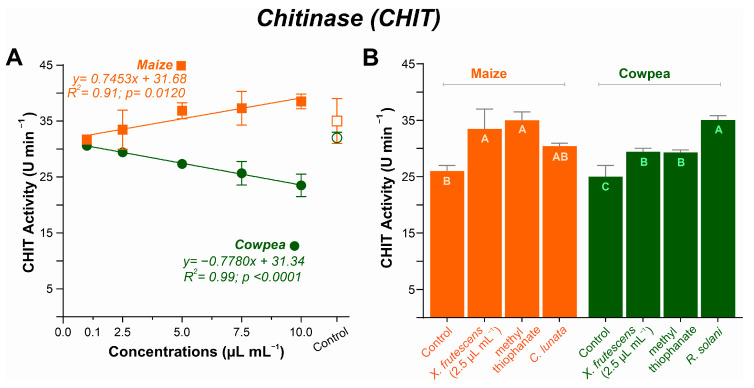
Chitinase (CHIT) activity in maize and cowpea plants exposed to different concentrations of *X. frutescens* essential oil (**A**); and expression of CHIT enzyme activity in different treatments (**B**). Symbols and bars show the mean (±SE) of three replicates; and bars with the same letter do not differ statistically between treatments of the same plant (Tukey test, *p* < 0.05).

Chitinase activity response to *X. frutescens* essential oil differed sharply between maize and cowpea. In maize, increasing concentrations caused a slight increase in enzyme activity compared to the control (from 35 U min^−1^ to a maximum of 38.5 U min^−1^), while in cowpea, increasing concentrations resulted in a slight decrease in CHIT activity, as compared to the control (from 32 U min^−1^ to a minimum of 23.5 U min^−1^) (Figure 8A).

Figure 8B compares CHIT responses among healthy plants, untreated diseased plants, and diseased plants treated with the fungicide or *X. frutescens* essential oil. Healthy plants showed the lowest CHI activity for both maize and cowpea. Compared with healthy plants CHI activity increased for *X. frutescens* essential oil, fungicide-treated or untreated diseased maize plants, with no significant differences among the diseased plant treatments. Similarly, CHI activity increased in *X. frutescens* essential oil, fungicide-treated or untreated diseased cowpea plants. However, for cowpea, diseased plants showed the highest enzyme activity (*p* < 0.05).

## 4. Discussion

### 4.1. Xylopia frutescens Essential Oil

#### 4.1.1. Essential Oil Content

Essential Oil Content EOC of 0.10% (*v*/*w*) obtained from *Xylopia frutescens* is considered relatively low, but remains within the reported range 0.05% to 1.5% for various Amazonian species known to produce sesquiterpene-rich essential oils [51]. The observed yield is influenced by numerous factors, including the specific phenological stage and the local environmental conditions [52,53]. Given the low yield, the primary focus shifts to the chemical composition, which dictates the oil’s biological potential.

#### 4.1.2. Chromatography

The composition of the essential oil of *X. frutescens* in this study determined four major compounds, being pinocarveol/isopinocarveol, (-)-spathulenol, myrtenal and *β*-pinene; representing 50.2% in total. Our results differs from studies conducted by [26,27,31,54], which had indicated *β*-caryophyllene, aromadendrene, *p*-cymene, (*E*)-caryophyllene, bicyclogermacrene, germacrene D, δ-cadinene, viridiflorene and α-copaene as major oil compounds. However, the compounds found in the different studies showed biological activity. Additionally, variability among chemotypes is a relevant aspect of the research of natural products, since the production of secondary metabolites is influenced by abiotic and biotic factors [55]. The predominance of oxygenated monoterpenes, such as nopinone and myrtenal, and sesquiterpenes such as spatulenol, has been suggested as specific plant metabolic responses to the environmental conditions of the collection site [56]. Most compounds found in this study are biologically relevant. *β*-pinene and nopinone, for example, are known for their antifungal and insecticidal properties [57,58], while spatulenol has shown strong antimicrobial, antioxidant [59], and insecticidal properties [60]. The high concentration of these compounds in the essential oil are probably factors contributing to the observed plant responses in this study.

#### 4.1.3. Thermal Analysis

The thermal stability of an essential oil depends on its composition. Thermogravimetric analysis of the essential oil of *X. frutescens* showed that it is highly volatile. The degradation of the oil compounds began at room temperature and at 94.74 °C mass loss reached approximately 32.5%. The loss can be attributed to the volatilization of the lighter compounds and is probably related to the XEO composition, which is rich in monoterpenes (C_10_) such as nopinone, β-pinene and myrtenal, and in the sesquiterpene spatulenol (C_15_).

The high XEO volatility stands out when compared to other oils studied previously [61,62]. Babassu oil, for example, presented a mass loss of 96.46% only at 397.26 °C [63], while the oil of *Siparuna guianenis* showed a degradation of only 54.21% even at 225 °C [64]. Therefore, the essential oil of *X. frutescens* is considerably more volatile than other studied plant essential oils, which can directly influence its biological activity and, consequently, its mode of application. Contrasting with the predominance of volatile compounds of low molecular mass found in the present study, an earlier report of another extraction of *X. frutescens* oil found a higher proportion of sesquiterpenes (72.38%) Mendes [54]. Oils with a majority of sesquiterpenes would likely degrade at higher temperature ranges. Thus, thermogravimetric analysis can be a useful tool for characterizing and differentiating oil chemotypes.

High volatility of the *X. frutescens* essential oil is, in practical terms, a limiting factor for field application, since its fast-thermal degradation suggests a rapid dissipation of the active compounds at the plant surface, resulting in low persistence and compromising the treatment’s effectiveness. To compensate for this disadvantage, frequent applications are required. Furthermore, the effectiveness of the treatment becomes dependent on environmental factors such as temperature, humidity, and other factors. Nevertheless, these limitations can be circumvented. Formulation strategies such as nanoemulsions [65], nanoencapsulation [66] or the use of adjuvants (spreading agents, adhesives and UV protectors) can increase XEO field stability and persistence [67,68,69]. Such approaches are promising for maximizing the biological activity of the oil in the field, enabling its use as a biofungicide.

### 4.2. Mycelial Growth

The present study demonstrates that *Xylopia frutescens* essential oil exerts measurable antifungal activity against *Curvularia lunata* and *Rhizoctonia solani*, as evidenced by its impact on mycelial growth dynamics and final colony diameter. For *C. lunata*, the essential oil treatment resulted in a statistically significant reduction in mycelial diameter compared to the control, despite a gradual increase in growth rate over time. Similarly, *R. solani* exhibited complete inhibition at early stages, followed by partial recovery, suggesting a fungistatic rather than fungicidal effect under the tested conditions. Studies have reported that plants from the Annonaceae family, such as *Xylopia aethiopica*, contain fungitoxic compounds capable of suppressing the growth of various microorganisms, including the phytopathogenic fungus [70,71]. Similarly, the essential oil of *Xylopia nitida* demonstrated antifungal activity at varying concentrations, inhibiting several phytopathogenic fungi such as *Corynespora* sp., *Helminthosporium* sp., *Macrophomina* sp., *Pestalodia dichaeta*, *Rhizoctonia solani*, *Sclerotium rolfsii*, *Colletotrichum gloesporioides, Clinipelis perniciosa*, *Cylindrocadium* sp., and *Phytophthora* spp. [72,73]. These findings underscore the potential of *Xylopia* species as natural sources of antifungal agents, offering a broad-spectrum alternative to synthetic fungicides and contributing to sustainable plant disease management strategies.

### 4.3. Phytotoxicity and Disease-Suppressing Effects on Maize and Cowpea Plants

A dose-dependent phytotoxic effect of *X. frutescens* oil on cowpea and maize plants was found. The observed leaf damage is probably caused by the damage of the bioactive components to the plant leaf cells [74]. The higher sensitivity of cowpeas, as compared to maize, suggests differences in metabolic pathways or cell membrane composition that make cowpeas more sensitive to the XEO secondary metabolites. These metabolites, mainly of terpenes and phenylpropanoids, are known for their stress-inducing capacity in plants [75]. Disease-suppressing effects, as measured by the AUDPCs, were quite relevant. The effectiveness of *X. frutescens* oil in suppressing the progress of maize Curvularia leaf spot and cowpea web blight (even at the low concentration of 2.5 µL mL^−1^), is promising, because at a low concentration, phytotoxicity is also low. The XEO effectiveness at low dosage calls attention to the oil mechanisms of action. The most recent literature points to a “double-mechanism” of action of essential oils in plant protection [24]. They can either act directly, inhibiting the growth of the pathogen and the germination of spores, or indirectly inducing the defenses of the host plant itself. Application of XEO to inoculated plants caused very low disease values, not statistically different from the healthy control plants, and significantly better than the reference chemical fungicide. This powerful disease containment effect was expressed in two pathosystems, including plants in widely different botanical families (one monocot and one dicot). Similar results have been observed for other essential oils at sub-phytotoxic concentrations and pathosystems [76], which are probably related to the activation the plant’s defense system against pathogens, mimicking a stress response. The potential of *X. frutescens* oil as a phytosanitary alternative lies in optimizing this concentration. Therefore, the efficacy demonstrated by XEO at 2.5 µL mL^−1^ implies potential for practical use. One next fundamental research step is to investigate whether there are additional direct antifungal effects to *Curvularia lunata* and *Rhizoctonia solani*, on top of the induction of plant resistance.

### 4.4. Chlorophyll

Chlorophyll content decreased proportionally to increasing XEO concentrations, indicating a direct impact on the photosynthetic process. Bioactive XEO compounds (e.g., terpenes), which can disrupt the integrity of cell membranes and organelles [77], are possibly responsible for the trend observed. Other essential oils, such as *Eucalyptus* oil, were reported to compromise the chloroplast structure, leading to the degradation of membrane-bound photosynthetic pigments [78]. Degradation of photosynthetic pigments was also correlated to oxidative stress, as reported by Li et al. [79], which aligns with the results observed for antioxidant enzymes, such as SOD and CAT, which were induced in response to the XEO treatment.

*X. frutescens* essential oil effects on chlorophyll content differed in pathogen-inoculated maize and cowpea. No measurable effect was noted on maize, indicating low phytotoxicity, significantly less than the treatment with chemical fungicide, and especially lower than in the Curvularia-infected plants. It is noteworthy that methyl thiophanate caused a significant reduction in chlorophyll content in diseased maize plants. Contrastingly, for cowpeas, chlorophyll levels in the XEO treatment and in the chemical fungicide treatment were not as significantly reduced as compared to in the untreated healthy plants. The most severe reduction in chlorophyll was recorded in the untreated cowpea plants infected with *R. solani*. The observed discrepancies in the responses of maize (monocot) and cowpea (dicot) suggests genetic and physiological differences in the ability to metabolize or tolerate essential oil compounds [80]. This distinction underscores the complexity of the interactions between the plant species and the essential oil. However, the initial dark condition of the experiment may have influenced the effects, since the absence of light may have accelerated the degradation of chlorophyll, which should also be considered.

### 4.5. Enzyme Activity

The application of *X. frutescens* essential oil significantly modulated the activity of several defense enzymes in maize and cowpea plants. The results suggest that XEO does not act directly on the stressor (the pathogen) but acts as a plant-defense elicitor, priming the plant’s defensive responses. This enzymatic modulation, which varies among crops and metabolic pathways, may be directly related to the major bioactive compounds of the oil, such as β-pinene, spatulenol and myrtenal.

Increased SOD activity, directly proportional to the increase in XEO concentrations, implies induction of an antioxidant response, which is related to tolerance to oxidative stresses [81]. SOD is the first line of defense against reactive oxygen species by catalyzing the dismutation of the superoxide radical (O_2_^•−^) in hydrogen peroxide (H_2_O_2_) and its higher activity in XEO-treated plants corroborates its role in the defense response [82]. In both maize and cowpea, XEO’s ability to induce SOD was comparable to that of the methyl thiophanate fungicide, evidencing its potential as a trigger of the plant defense system. The activation of SOD in response to stress by essential oils or other elicitors is a phenomenon reported by other authors, such as Dias et al. [83], who observed the same pattern on melon (*Cucumis melo*). Interestingly, the importance of SOD in stress responses is not restricted to plants, but has also been reported in endophytic fungi [20].

The dynamics between SOD and enzymes that neutralize hydrogen peroxide—CAT and APX—are noteworthy. While SOD increased, CAT and APx activity showed a decreasing trend, indicating that XEO concentrations above 5.0 µL mL^−1^ may be phytotoxic, causing biochemical imbalances. However, the enzymes remained functional and may have contributed to the plant’s defense by breaking down the high levels of hydrogen peroxide without the need for an additional reductant [84,85]. This does not represent a failure in the defense system, but an indication that the plant may be selectively modulating its response pathways to different types of stress. It is possible that H_2_O_2_ generated by SOD is being metabolized by other pathways of the ascorbate–glutathione cycle or that XEO acts specifically in the regulation of these enzymes [86,87], as well as the APX [88]. The present results from maize and cowpea indicated that *Xylopia* oil induced a significant increase in the enzyme, suggesting a specific response of the plant’s antioxidant system to the essential oil. The importance of APx in defense systems has been observed earlier in extracts of *Lippia sidoides* [20].

Growing concentrations of XEO resulted in a slight increase in the activity of chitinase in maize plants. However, in cowpeas, a decreasing trend in CHIT activity was observed, a result similar to that found in melon plants [83]. With CHIT activity levels that resemble those of the commercial fungicide in pathogen-challenged plants, the plant response is probably optimized, especially in maize, since chitinases act as a defense against fungi, degrading the chitin of the fungal cell wall [89,90]. The highest activity of CHIT in the diseased cowpea plants infected by *R. solani* highlights the role of this enzyme in the plant response to infection. The absence of a significant increase in CHIT with the application of XEO in cowpeas, in contrast to its induction by other plant extracts [91], suggests that the oil acts indirectly, by eliciting other defense mechanisms. In conclusion, the heretofore scarcity of information about the enzymatic activity elicited by *X. frutescens* essential oil in plant species from widely different families underscores the pertinence of this study.

## 5. Conclusions

The *Xylopia frutescens* essential oil rich in terpenes has promising potential for activating several plant response processes to biotic stressors. Here, it has been demonstrated that *X. frutescens* has the capacity to inhibit the progression of fungal diseases, potentially by inducing physiological defense responses through the activation of the plant’s antioxidant system. In vitro assays confirmed the antifungal activity of *X. frutescens* against phytopathogens, reinforcing its potential for use in integrated disease management strategies. Although, a concentration-dependent phytotoxic effect was observed and could be a challenge for its application in the field; future studies directed to mitigate these effects, and increase its stability and persistence in the field by adaptative formulation strategies are essential. These findings validate the *Xylopia frutescens* as a promising alternative with a potentially low environmental impact, highlighting its potential as an effective biorational for the sustainable management of diseases in field crops.

## Figures and Tables

**Table 1:** *Xylopia frutescens* essential oil components were identified by gas chromatography coupled with mass spectrometry (GC-MS).

Compound	Formula	Classification	* Ri Experimental	* Ri Literature [48,49]	%
α-Pinene	C_10_H_16_	Hydrocarbon ^c^	948	939	9.6
Bicyclo[3.1.0]hex-2-ene, 4-methylene-1-(1-methylethyl)- (dehydrosabinene-type)	C_10_H_14_	Hydrocarbon ^c^	879	879	1.3
β-Pinene	C_10_H_16_	Hydrocarbon ^c^	943	980	11.0
p-Cymene	C_10_H_14_	Hydrocarbon ^e^	1042	1026	1.3
Eucalyptol (1,8-cineole)	C_10_H_18_O	Ether ^a^	1059	1032	1.8
α-Campholenal	C_10_H_16_O	Aldehyde ^a^	1155	1155	1.5
Bicyclo[3.1.1]heptan-2-one, 6,6-dimethyl-, (1R)- (nopinone-type)	C_9_H_14_O	ketone ^a^	1047	1047	2.4
Bicyclo[3.1.1] heptan-3-ol, 6,6-dimethyl-2-methylene- (Pinocarveol/isopinocarveol)	C_10_H_16_O	Alcohol ^a^	1131	1184	13.8
Verbenol	C_10_H_16_O	Alcohol ^a^	1136	1136	4.3
Pinocarvone	C_10_H_14_O	Ketone ^a^	1114	1162	5.8
p-Mentha-1,5-dien-8-ol	C_10_H_16_O	Alcohol ^a^	1125	1125	1.9
Bicyclo[3.1.1] hept-2-ene-2-carboxaldehyde, 6,6-dimethyl- (myrtenal)	C_10_H_14_O	Aldehyde ^a^	1136	1170	12.5
Bicyclo[3.1.1] hept-2-ene-2-methanol, 6,6-dimethyl- (myrtenol)	C_10_H_16_O	Alcohol ^a^	1191	1198	6.0
Bicyclo[3.1.1] hept-3-en-2-one, 4,6,6-trimethyl- (Verbenone/2-pinen-4-one)	C_10_H_14_O	ketone ^a^	1119	1204	3.0
Cyclohexene, 4-ethenyl-4-methyl-3-(1-methylethenyl)-1-(1-methylethyl)- (sesquiterpene hydrocarbon)	C_15_H_24_	Hydrocarbon ^d^	1377	1377	0.9
Germacrene D	C_15_H_24_	Hydrocarbon ^d^	1515	1515	4.0
(-)-Spathulenol	C_15_H_24_O	Alcohol ^b^	1536	1536	12.9
Caryophyllene oxide	C_15_H_24_O	Epoxide ^b^	1507	1507	1.1
Isospathulenol	C_15_H_24_O	Alcohol ^b^	1623	1623	4.1
Total identified (%)					99.2
^a^ Oxygenated monoterpenes (%)					50.0
^b^ Oxigenated sesquiterpenes (%)					20.0
^c^ Monoterpene (%)					15.0
^d^ Sesquiterpene (%)					10.0
^e^ Aromatic monoterpene (%)					5.0

* Retention index; classification compound with the same lower case (columns 1 and 3) belong to the same secondary metabolite group.

**Table 2:** Mycelial Growth Rate (mm/day) of *C. lunata* and *R. solani* over time when treated with essential oil.

Fungi	Treatment	Mycelial Growth Rate (mm/Day ± SE)					MMGR (mm/Day)
		Day 2	Day 4	Day 6	Day 8	Day 10	
*Curvularia lunata*	Control (water and Tween 80)	12.2 ± 0.3	13.2 ± 0.1	9.2 ± 0.4	8.4 ± 0.3	2.2 ± 0	9.0
	*Xylopia frutescens* (2.5 µL mL^−1^)	6.2 ± 0.3	6.4 ± 0.3	9.4 ± 0.5	10.2 ± 1.0	10.9 ± 0.1	8.6
	Methyl thiophanate (20 µg mL^−1^)	10.8 ± 0.9	11.2 ± 0.7	11.4 ± 0.4	6.5 ± 1.3	4.6 ± 0.6	9.0
*Rhizoctonia solani*	Control (water and Tween 80)	35.8 ± 0.3	9.7 ± 0.5	0.0 ± 0	0.0 ± 0	0.0 ± 0	9.0
	*Xylopia frutescens* (2.5 µL mL^−1^)	0 ± 0	5.8 ± 0.3	9.4 ± 0.5	13.2 ± 1.0	13.2 ± 0.8	8.5
	Methyl thiophanate (20 µg mL^−1^)	35.8 ± 0.9	9.2 ± 0.7	0.0 ± 0	0.0 ± 0	0.0 ± 0	9.0

MMGR: mean mycelial growth rate.

## Data Availability

All data generated and/or analyzed during the present study are available in the manuscript, and the corresponding author has no objection to the availability of data and materials upon reasonable request.

## Supplementary Materials

The following supporting information can be downloaded at https://www.mdpi.com/article/10.3390/microorganisms13112571/s1, File S1: Chromatogram.

## Abbreviations

The following abbreviations are used in this manuscript:

SOD	Superoxide dismutase
CAT	Catalase
APx	Ascorbate peroxidase
XEO	*Xylopia frutescens* essential oil
CHIT	Chitinase
ROS	Reactive oxygen species
TGA	Thermogravimetric analysis
GC-MS	Gas chromatography coupled with mass spectrometry
EO	Essential oil
H_2_O_2_	Hydrogen peroxide
