# Supplementary material for: Effect of Xylopia frutescens Essential Oil on the Activation of Defense Mechanisms Against Phytopathogenic Fungi
Source: Microorganisms. 2025 Nov 11;13(11):2571. doi: 10.3390/microorganisms13112571 (PMC12654693; doi:10.3390/microorganisms13112571)

Chromatogram XYLOPSIA SP. C:\GCMSsolution\Data\Project1\29491A.QGD

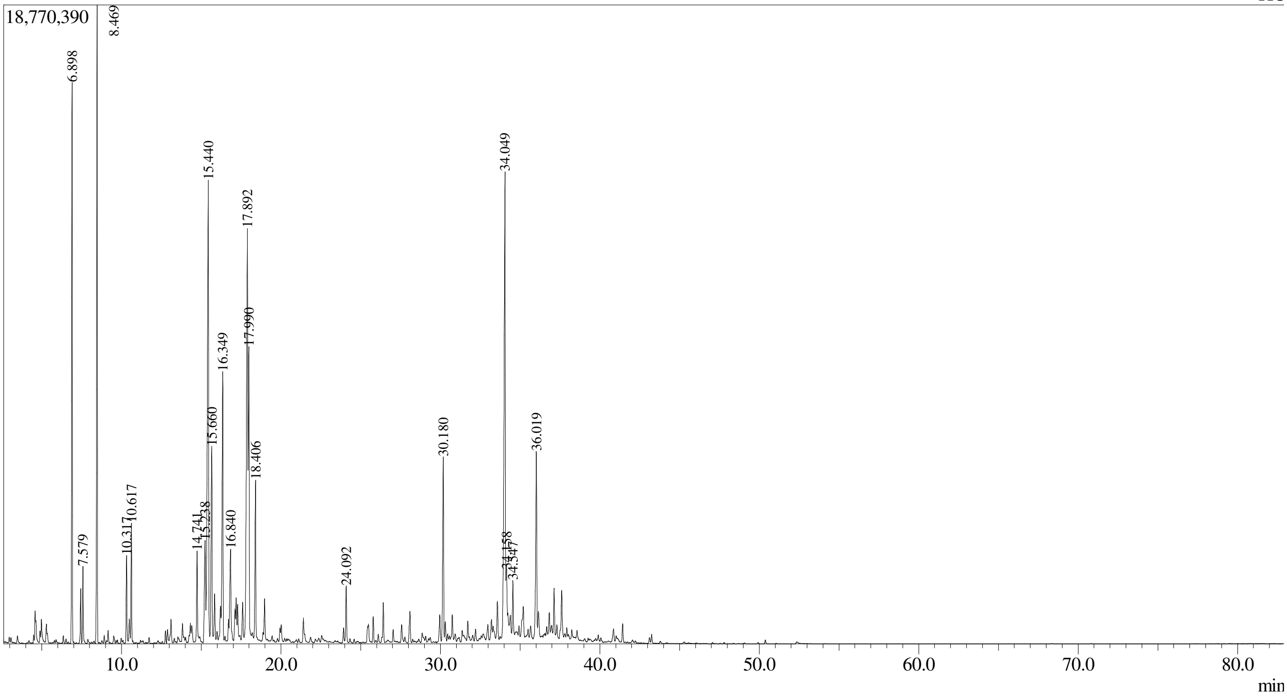

Peak Report TIC

Peak#	R.Time	I.Time	F.Time	Area	Area%	Height	A/H	Name
1	6.898	6.817	6.958	49538369	9.56	16450383	3.01	.alpha.-Pinene
2	7.579	7.525	7.642	6658580	1.28	2182659	3.05	Bicyclo[3.1.0]hex-2-ene, 4-methylene-1-(1-me
3	8.469	8.383	8.525	57131009	11.02	18697154	3.06	.beta.-Pinene
4	10.317	10.267	10.375	6847857	1.32	2402986	2.85	p-Cymene
5	10.617	10.567	10.675	9249462	1.78	3353840	2.76	Eucalyptol
6	14.741	14.683	14.800	7555234	1.46	2414110	3.13	.alpha.-Campholenal
7	15.238	15.158	15.292	12346893	2.38	2685467	4.60	Bicyclo[3.1.1]heptan-2-one, 6,6-dimethyl-, (1I
8	15.440	15.292	15.525	71258142	13.75	13345328	5.34	Bicyclo[3.1.1]heptan-3-ol, 6,6-dimethyl-2-met
9	15.660	15.567	15.717	22238963	4.29	5488105	4.05	Verbenol
10	16.349	16.258	16.408	29810405	5.75	7562576	3.94	Pinocarvone
11	16.840	16.758	16.900	9867269	1.90	2303018	4.28	p-Mentha-1,5-dien-8-ol
12	17.892	17.750	17.933	64617361	12.47	11740414	5.50	Bicyclo[3.1.1]hept-2-ene-2-carboxaldehyde, 6
13	17.990	17.933	18.050	31112761	6.00	8284773	3.76	Bicyclo[3.1.1]hept-2-ene-2-methanol, 6,6-dim
14	18.406	18.325	18.458	15683935	3.03	4476647	3.50	Bicyclo[3.1.1]hept-3-en-2-one, 4,6,6-trimethyl
15	24.092	24.042	24.158	4841235	0.93	1475011	3.28	Cyclohexene, 4-ethenyl-4-methyl-3-(1-methyle
16	30.180	30.092	30.250	20591502	3.97	5255563	3.92	Germacrene D
17	34.049	33.900	34.100	67075970	12.94	13092955	5.12	(-)-Spathulenol
18	34.158	34.100	34.208	5805522	1.12	1348811	4.30	Caryophyllene oxide
19	34.547	34.500	34.617	4593361	0.89	1368495	3.36	(-)-Spathulenol
20	36.019	35.925	36.083	21433986	4.14	5093756	4.21	Isospathulenol
				518257816	100.00	129022051		

Spectrum

Peak#:1 R.Time:6.898(Scan#:517)
MassPeaks:77
RawMode:Averaged 6.892-6.908(516-518)
BG Mode:Calc. from Peak Group 1 - Event 1 Scan

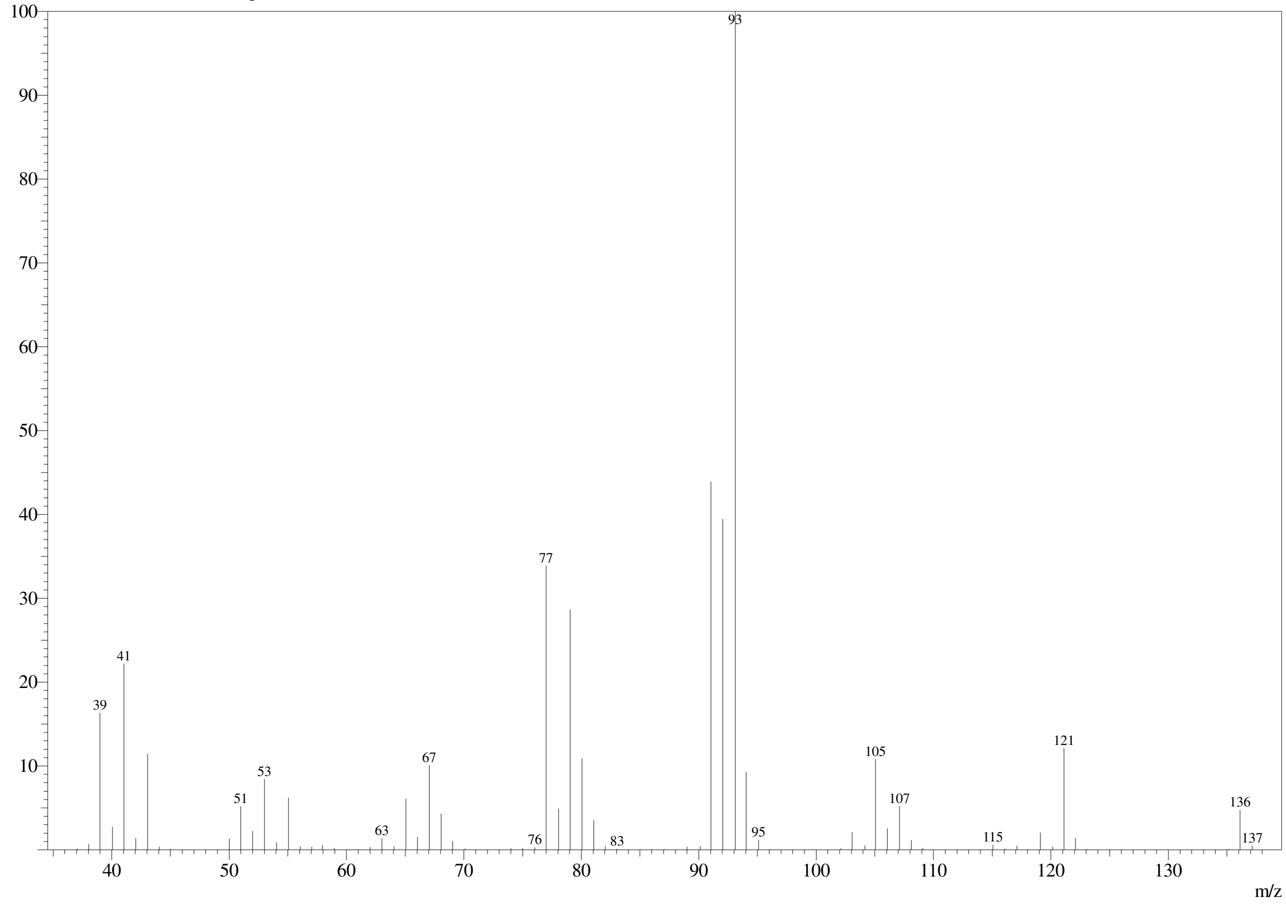

Mass Table
Peak#:1 R.Time:6.900(Scan#:517)
MassPeaks:77
Group 1 - Event 1 Scan

#	m/z	Rel. Int.	#	m/z	Rel. Int.	#	m/z	Rel. Int.	#	m/z	Rel. Int.
1	37.05	0.14	21	58.95	0.22	41	81.05	3.56	61	106.05	2.55
2	38.05	0.69	22	59.95	0.04	42	82.05	0.43	62	107.10	5.21
3	39.00	16.32	23	61.00	0.06	43	83.05	0.11	63	108.10	1.13
4	40.05	2.76	24	62.00	0.35	44	83.95	0.02	64	109.10	0.23
5	41.05	22.23	25	63.00	1.39	45	84.95	0.03	65	110.10	0.01
6	42.05	1.42	26	64.05	0.41	46	85.95	0.07	66	115.05	0.60
7	43.05	11.45	27	65.05	6.10	47	87.05	0.06	67	116.10	0.13
8	44.05	0.37	28	66.05	1.53	48	89.00	0.37	68	117.10	0.48
9	45.00	0.13	29	67.05	10.13	49	90.15	0.47	69	118.15	0.08
10	45.95	0.04	30	68.05	4.30	50	91.05	43.94	70	119.10	2.06
11	49.00	0.05	31	69.05	1.08	51	92.05	39.47	71	120.15	0.39
12	50.00	1.32	32	70.10	0.15	52	93.10	100.00	72	121.10	12.13
13	51.00	5.19	33	71.05	0.05	53	94.05	9.27	73	122.10	1.40
14	52.00	2.25	34	74.00	0.18	54	95.10	1.19	74	123.10	0.08
15	53.00	8.42	35	75.00	0.20	55	96.10	0.08	75	135.15	0.12
16	54.05	0.93	36	76.05	0.32	56	101.05	0.02	76	136.10	4.75
17	55.05	6.21	37	77.00	33.88	57	102.10	0.20	77	137.15	0.51
18	56.05	0.41	38	78.05	4.92	58	103.05	2.08			
19	57.05	0.38	39	79.05	28.67	59	104.15	0.53			
20	57.95	0.58	40	80.05	10.90	60	105.05	10.80			

Spectrum

Peak#:2   R.Time:7.579(Scan#:598)
MassPeaks:70
RawMode:Averaged 7.567-7.583(597-599)
BG Mode:Calc. from Peak Group 1 - Event 1 Scan

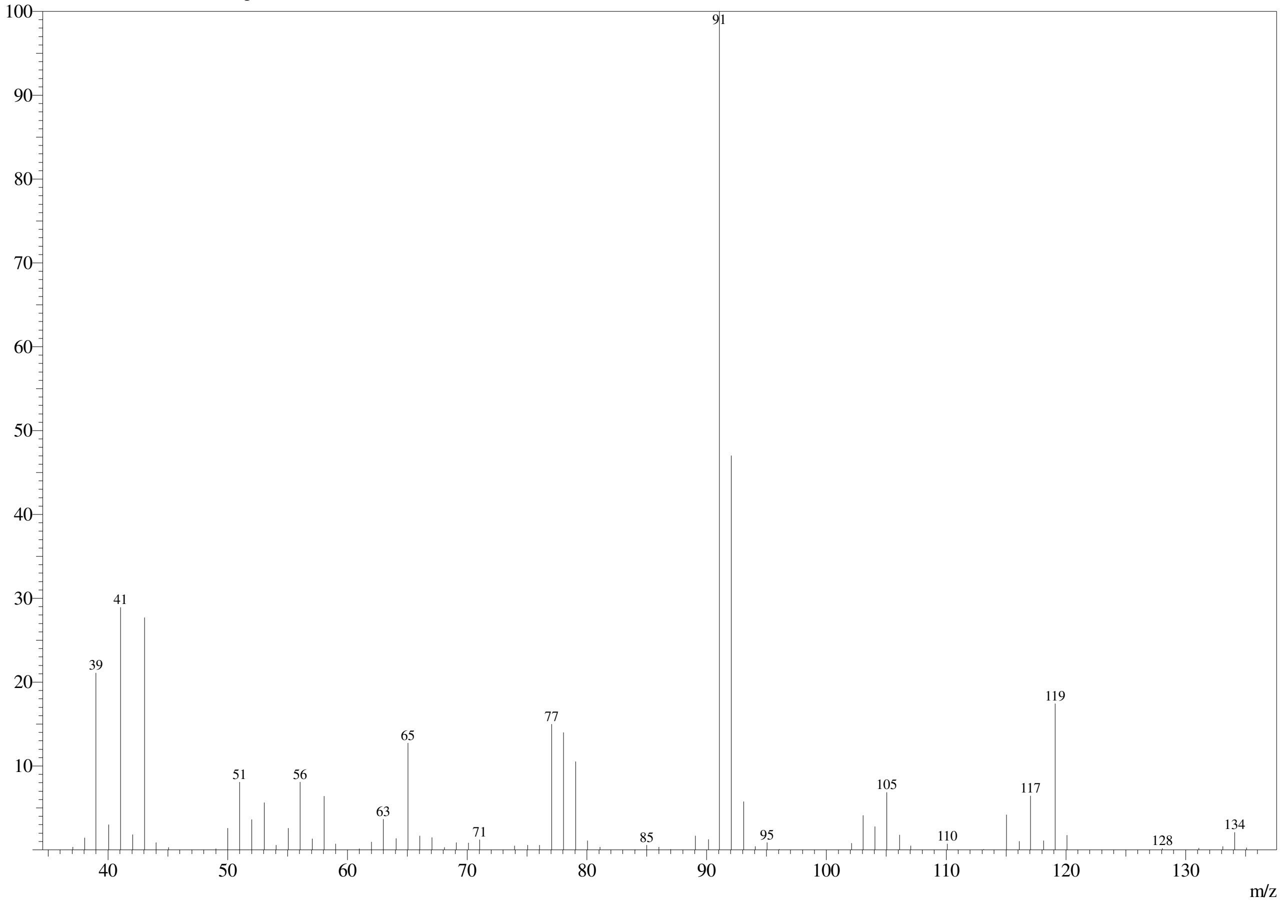

Mass Table
Peak#:2   R.Time:7.575(Scan#:598)
MassPeaks:70
Group 1 - Event 1 Scan

#	m/z	Rel. Int.	#	m/z	Rel. Int.	#	m/z	Rel. Int.	#	m/z	Rel. Int.
1	37.05	0.35	19	58.05	6.41	37	79.05	10.52	55	105.05	6.87
2	38.05	1.44	20	59.00	0.74	38	80.05	1.11	56	106.10	1.78
3	39.00	21.11	21	61.00	0.13	39	81.10	0.34	57	107.05	0.49
4	40.05	3.00	22	62.00	0.94	40	84.00	0.00	58	110.10	0.72
5	41.05	28.92	23	63.00	3.65	41	85.00	0.58	59	113.15	0.07
6	42.05	1.82	24	64.05	1.37	42	86.00	0.33	60	115.05	4.20
7	43.05	27.72	25	65.05	12.74	43	87.00	0.07	61	116.10	1.04
8	44.00	0.90	26	66.05	1.69	44	89.05	1.68	62	117.05	6.47
9	45.05	0.30	27	67.05	1.47	45	90.15	1.27	63	118.15	1.09
10	49.00	0.15	28	68.10	0.29	46	91.05	100.00	64	119.10	17.45
11	50.00	2.58	29	69.10	0.87	47	92.05	47.04	65	120.10	1.75
12	51.00	8.11	30	70.10	0.83	48	93.10	5.77	66	128.05	0.18
13	52.00	3.61	31	71.05	1.22	49	94.05	0.43	67	131.10	0.23
14	53.05	5.64	32	73.95	0.49	50	95.05	0.89	68	133.10	0.42
15	54.05	0.58	33	75.05	0.59	51	96.05	0.07	69	134.10	2.10
16	55.05	2.61	34	76.05	0.57	52	102.10	0.82	70	135.10	0.25
17	56.05	8.11	35	77.05	14.99	53	103.05	4.13			
18	57.05	1.33	36	78.05	13.99	54	104.05	2.79			

Spectrum

Peak#:3 R.Time:8.469(Scan#:705)
MassPeaks:76
RawMode:Averaged 8.458-8.475(704-706)
BG Mode:Calc. from Peak Group 1 - Event 1 Scan

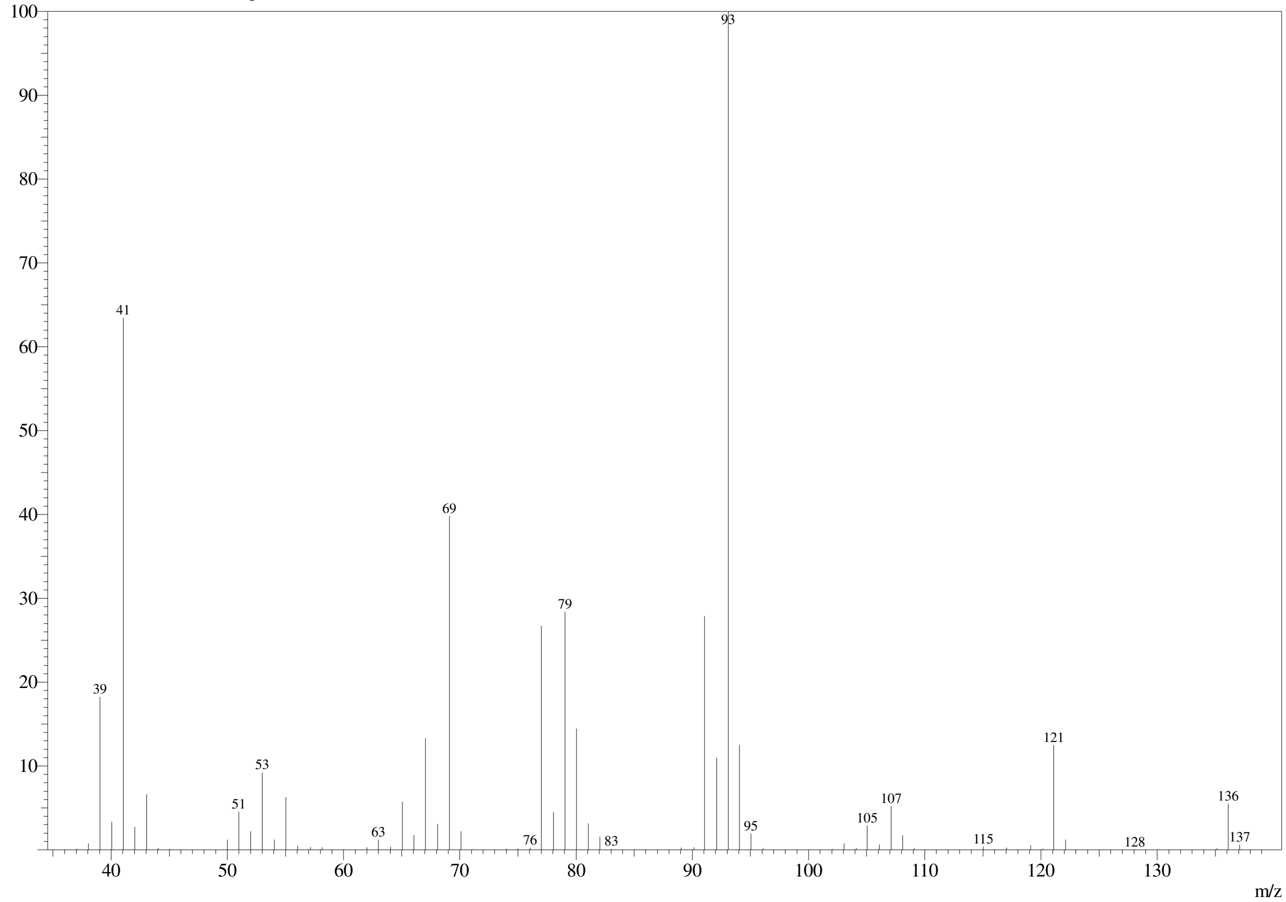

Mass Table
Peak#:3 R.Time:8.467(Scan#:705)
MassPeaks:76
Group 1 - Event 1 Scan

#	m/z	Rel. Int.	#	m/z	Rel. Int.	#	m/z	Rel. Int.	#	m/z	Rel. Int.
1	37.05	0.15	20	58.15	0.32	39	80.05	14.45	58	105.05	2.89
2	38.05	0.78	21	59.15	0.05	40	81.05	3.18	59	106.10	0.66
3	39.05	18.26	22	61.00	0.06	41	82.05	1.55	60	107.10	5.23
4	40.05	3.35	23	62.00	0.30	42	83.05	0.13	61	108.10	1.74
5	41.05	63.46	24	63.00	1.22	43	84.00	0.03	62	109.10	0.20
6	42.05	2.76	25	64.05	0.36	44	86.00	0.06	63	115.05	0.36
7	43.05	6.66	26	65.05	5.73	45	87.00	0.04	64	116.05	0.08
8	44.05	0.25	27	66.05	1.77	46	89.05	0.28	65	117.05	0.25
9	45.05	0.11	28	67.05	13.30	47	90.15	0.29	66	118.10	0.04
10	45.95	0.04	29	68.10	3.11	48	91.05	27.87	67	119.10	0.52
11	49.00	0.05	30	69.10	39.79	49	92.10	11.01	68	120.15	0.16
12	50.00	1.23	31	70.10	2.21	50	93.10	100.00	69	121.10	12.48
13	51.00	4.56	32	71.05	0.07	51	94.05	12.50	70	122.10	1.21
14	52.00	2.21	33	74.00	0.14	52	95.05	1.93	71	123.10	0.05
15	53.00	9.24	34	75.00	0.15	53	96.10	0.20	72	128.05	0.01
16	54.05	1.22	35	76.05	0.27	54	97.15	0.03	73	135.15	0.17
17	55.05	6.29	36	77.00	26.70	55	102.10	0.11	74	136.10	5.50
18	56.05	0.51	37	78.05	4.53	56	103.05	0.76	75	137.10	0.59
19	57.15	0.36	38	79.05	28.38	57	104.10	0.23	76	138.20	0.02

Spectrum

Peak#:4 R.Time:10.317(Scan#:927)
MassPeaks:65
RawMode:Averaged 10.308-10.325(926-928)
BG Mode:Calc. from Peak Group 1 - Event 1 Scan

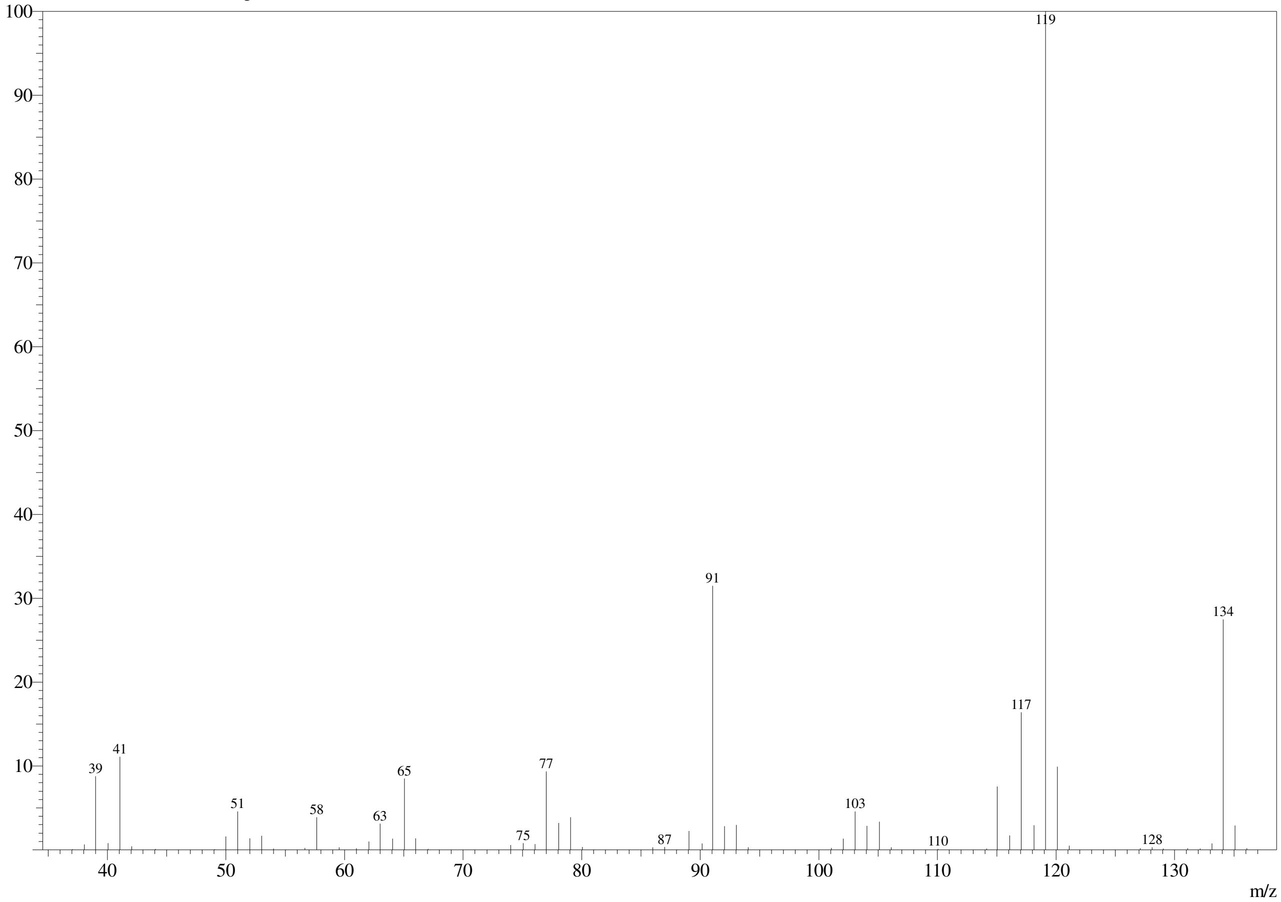

Mass Table
Peak#:4 R.Time:10.317(Scan#:927)
MassPeaks:65
Group 1 - Event 1 Scan

#	m/z	Rel. Int.	#	m/z	Rel. Int.	#	m/z	Rel. Int.	#	m/z	Rel. Int.
1	37.05	0.09	18	61.00	0.17	35	88.05	0.05	52	117.05	16.42
2	38.05	0.67	19	62.05	0.98	36	89.05	2.26	53	118.15	2.96
3	39.00	8.78	20	63.00	3.13	37	90.15	0.75	54	119.10	100.00
4	40.05	0.82	21	64.05	1.35	38	91.05	31.48	55	120.10	9.92
5	41.05	11.11	22	65.05	8.51	39	92.05	2.83	56	121.10	0.48
6	42.05	0.44	23	66.00	1.36	40	93.05	2.97	57	127.10	0.18
7	44.05	0.11	24	67.05	0.11	41	94.05	0.30	58	128.10	0.31
8	49.05	0.08	25	74.00	0.57	42	101.05	0.22	59	129.05	0.15
9	50.00	1.61	26	75.05	0.79	43	102.05	1.32	60	131.10	0.18
10	51.00	4.57	27	76.05	0.70	44	103.05	4.57	61	132.15	0.14
11	52.00	1.36	28	77.00	9.35	45	104.05	2.85	62	133.15	0.77
12	53.00	1.67	29	78.05	3.19	46	105.10	3.38	63	134.10	27.49
13	54.00	0.16	30	79.05	3.89	47	106.10	0.32	64	135.10	2.89
14	55.05	0.04	31	80.05	0.34	48	110.05	0.12	65	136.10	0.19
15	56.65	0.23	32	81.10	0.04	49	114.15	0.15			
16	57.65	3.90	33	86.00	0.32	50	115.05	7.56			
17	59.55	0.32	34	87.00	0.33	51	116.10	1.72			

Spectrum

Peak#:5   R.Time:10.617(Scan#:963)
MassPeaks:69
RawMode:Averaged 10.608-10.625(962-964)
BG Mode:Calc. from Peak Group 1 - Event 1 Scan

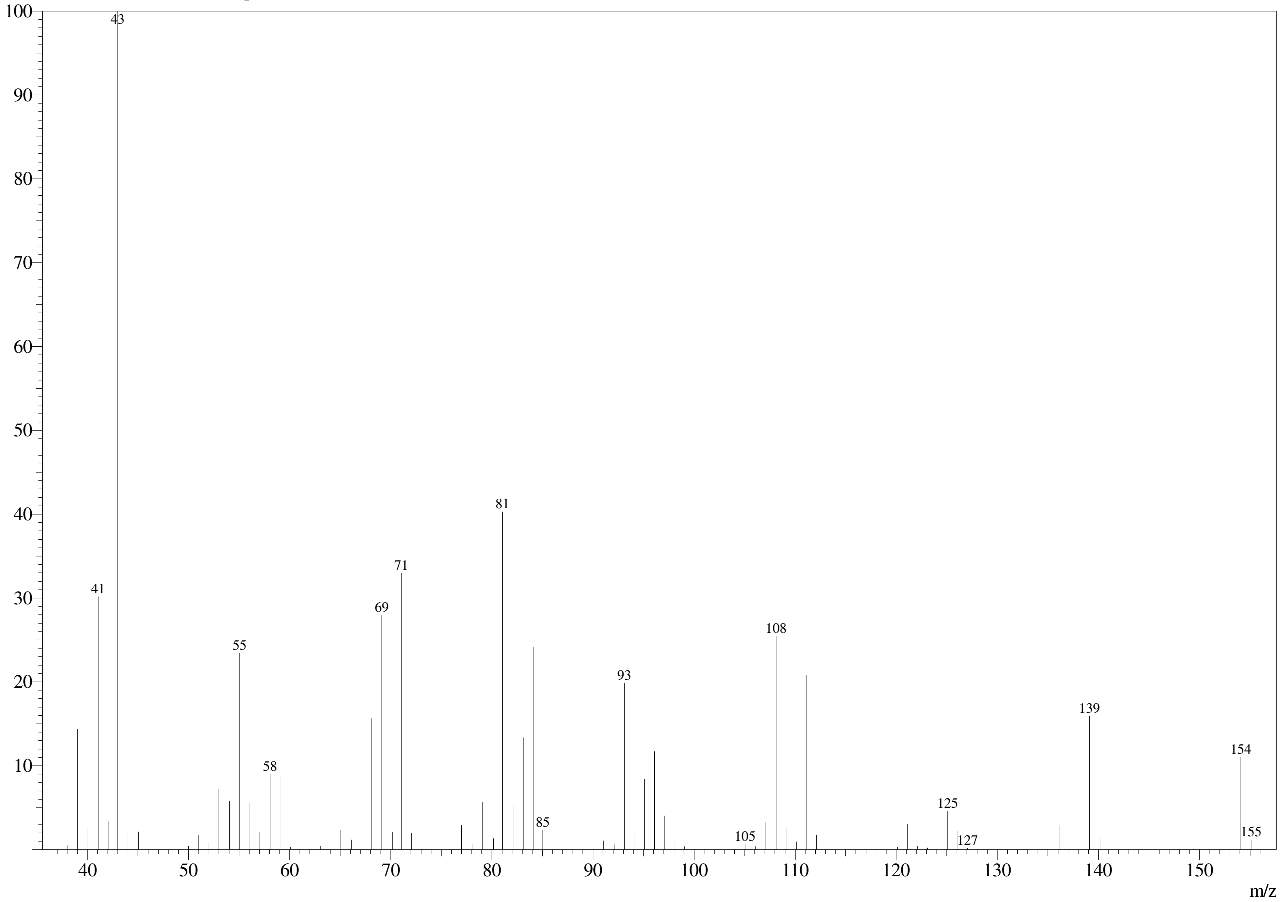

Mass Table
Peak#:5   R.Time:10.617(Scan#:963)
MassPeaks:69
Group 1 - Event 1 Scan

#	m/z	Rel. Int.	#	m/z	Rel. Int.	#	m/z	Rel. Int.	#	m/z	Rel. Int.
1	38.05	0.49	19	59.05	8.74	37	83.10	13.34	55	111.10	20.80
2	39.00	14.35	20	60.10	0.34	38	84.10	24.17	56	112.10	1.73
3	40.05	2.72	21	63.05	0.41	39	85.05	2.33	57	120.10	0.29
4	41.05	30.20	22	65.05	2.32	40	91.05	1.09	58	121.10	3.07
5	42.05	3.36	23	66.10	1.20	41	92.15	0.60	59	122.10	0.40
6	43.00	100.00	24	67.05	14.78	42	93.10	19.88	60	123.10	0.24
7	44.00	2.35	25	68.05	15.66	43	94.05	2.18	61	125.10	4.61
8	45.05	2.14	26	69.10	27.97	44	95.10	8.42	62	126.10	2.24
9	48.95	0.02	27	70.15	2.10	45	96.10	11.73	63	127.05	0.19
10	50.00	0.47	28	71.05	33.02	46	97.10	4.04	64	136.10	2.94
11	51.00	1.75	29	72.05	1.94	47	98.10	1.04	65	137.10	0.46
12	52.00	0.82	30	73.10	0.13	48	99.05	0.37	66	139.10	15.90
13	53.00	7.21	31	77.00	2.89	49	105.05	0.64	67	140.15	1.49
14	54.05	5.75	32	78.05	0.69	50	106.10	0.40	68	154.10	11.05
15	55.05	23.42	33	79.05	5.70	51	107.10	3.24	69	155.10	1.18
16	56.05	5.56	34	80.15	1.34	52	108.10	25.50			
17	57.05	2.09	35	81.05	40.32	53	109.10	2.57			
18	58.05	9.02	36	82.10	5.29	54	110.15	0.96			

Peak#:6 R.Time:14.741(Scan#:1458)
MassPeaks:62
RawMode:Averaged 14.733-14.750(1457-1459)
BG Mode:Calc. from Peak Group 1 - Event 1 Scan

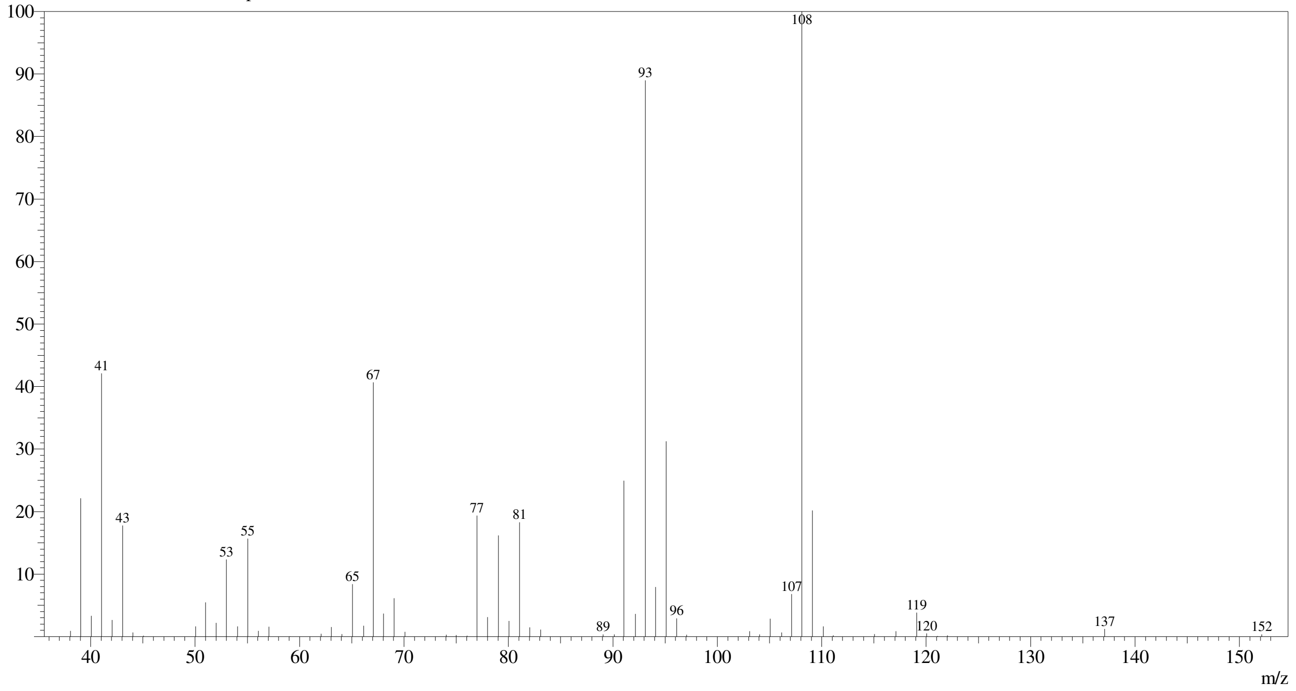

Mass Table
Peak#:6 R.Time:14.742(Scan#:1458)
MassPeaks:62
Group 1 - Event 1 Scan

#	m/z	Rel. Int.	#	m/z	Rel. Int.	#	m/z	Rel. Int.	#	m/z	Rel. Int.
1	38.05	0.90	17	59.05	0.02	33	80.05	2.49	49	106.15	0.66
2	39.05	22.12	18	62.05	0.45	34	81.05	18.30	50	107.10	6.82
3	40.05	3.31	19	63.05	1.53	35	82.05	1.49	51	108.10	100.00
4	41.05	42.10	20	64.05	0.39	36	83.10	1.13	52	109.10	20.20
5	42.05	2.66	21	65.05	8.39	37	89.05	0.34	53	110.15	1.66
6	43.05	17.76	22	66.15	1.72	38	90.15	0.36	54	111.10	0.20
7	44.05	0.68	23	67.05	40.68	39	91.05	24.94	55	115.05	0.40
8	45.05	0.15	24	68.05	3.70	40	92.15	3.66	56	117.10	0.87
9	50.05	1.65	25	69.05	6.14	41	93.10	88.99	57	119.10	3.87
10	51.00	5.50	26	70.10	0.75	42	94.10	7.94	58	120.05	0.49
11	52.00	2.19	27	74.05	0.29	43	95.10	31.23	59	121.10	0.08
12	53.00	12.32	28	75.00	0.26	44	96.10	2.90	60	122.05	0.18
13	54.05	1.64	29	76.05	0.17	45	97.05	0.33	61	137.10	1.24
14	55.05	15.70	30	77.00	19.37	46	103.10	0.86	62	152.15	0.35
15	56.05	0.95	31	78.00	3.13	47	104.05	0.34			
16	57.05	1.58	32	79.05	16.21	48	105.05	2.89			

Peak#:7 R.Time:15.238(Scan#:1518)
MassPeaks:60
RawMode:Averaged 15.233-15.250(1517-1519)
BG Mode:Calc. from Peak Group 1 - Event 1 Scan

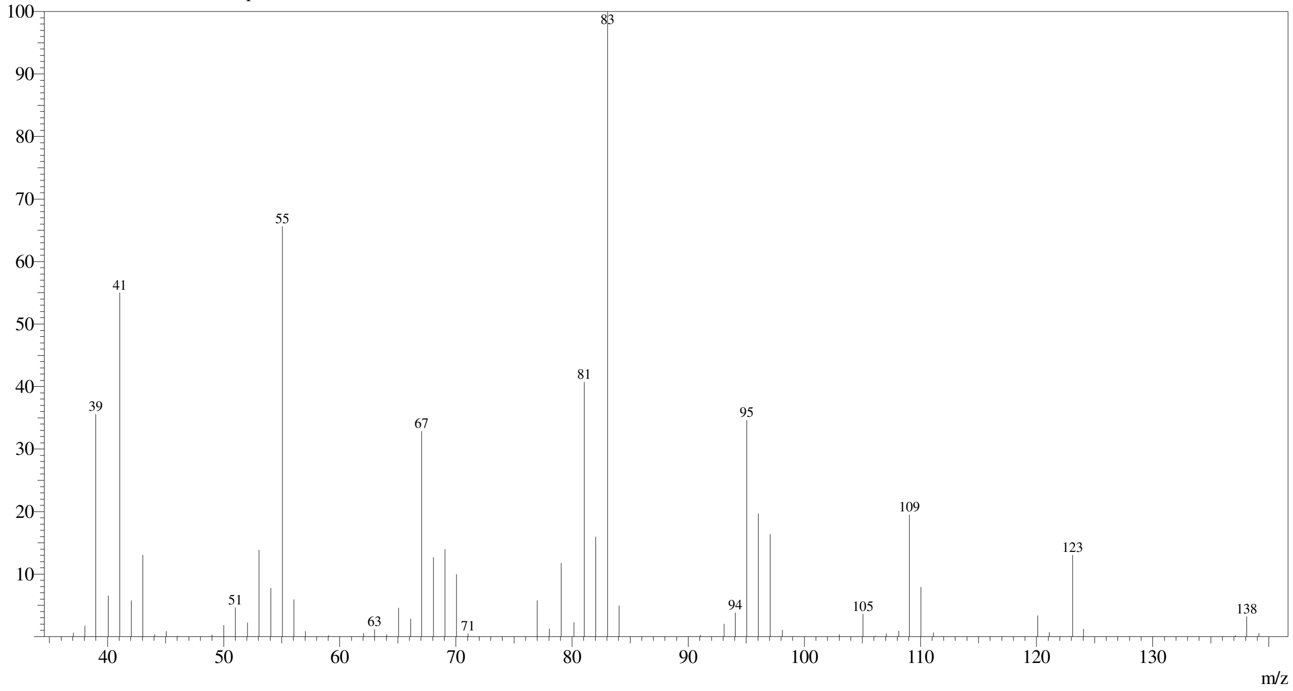

Mass Table
Peak#:7 R.Time:15.242(Scan#:1518)
MassPeaks:60
Group 1 - Event 1 Scan

#	m/z	Rel. Int.	#	m/z	Rel. Int.	#	m/z	Rel. Int.	#	m/z	Rel. Int.
1	37.05	0.60	16	54.05	7.78	31	77.00	5.79	46	103.00	0.33
2	38.05	1.74	17	55.05	65.63	32	78.05	1.31	47	105.05	3.62
3	39.00	35.63	18	56.05	5.93	33	79.05	11.76	48	106.10	0.13
4	40.05	6.55	19	57.05	0.85	34	80.15	2.32	49	107.05	0.47
5	41.05	55.00	20	59.05	0.20	35	81.05	40.72	50	108.15	0.94
6	42.05	5.78	21	62.05	0.56	36	82.05	15.98	51	109.05	19.50
7	43.05	13.08	22	63.00	1.17	37	83.05	100.00	52	110.05	7.92
8	44.05	0.33	23	64.05	0.38	38	84.05	4.96	53	111.10	0.67
9	45.05	0.85	24	65.05	4.62	39	91.05	0.26	54	120.10	3.41
10	46.05	0.10	25	66.10	2.88	40	93.10	2.04	55	121.10	0.74
11	49.00	0.32	26	67.05	32.91	41	94.05	3.86	56	123.10	13.04
12	50.00	1.83	27	68.05	12.68	42	95.05	34.63	57	124.05	1.22
13	51.00	4.65	28	69.05	13.96	43	96.05	19.69	58	137.10	0.18
14	52.05	2.27	29	70.05	9.97	44	97.05	16.40	59	138.10	3.24
15	53.05	13.91	30	71.05	0.49	45	98.10	1.08	60	139.15	0.54

Peak#:8 R.Time:15.440(Scan#:1542)
MassPeaks:94
RawMode:Averaged 15.433-15.450(1541-1543)
BG Mode:Calc. from Peak Group 1 - Event 1 Scan

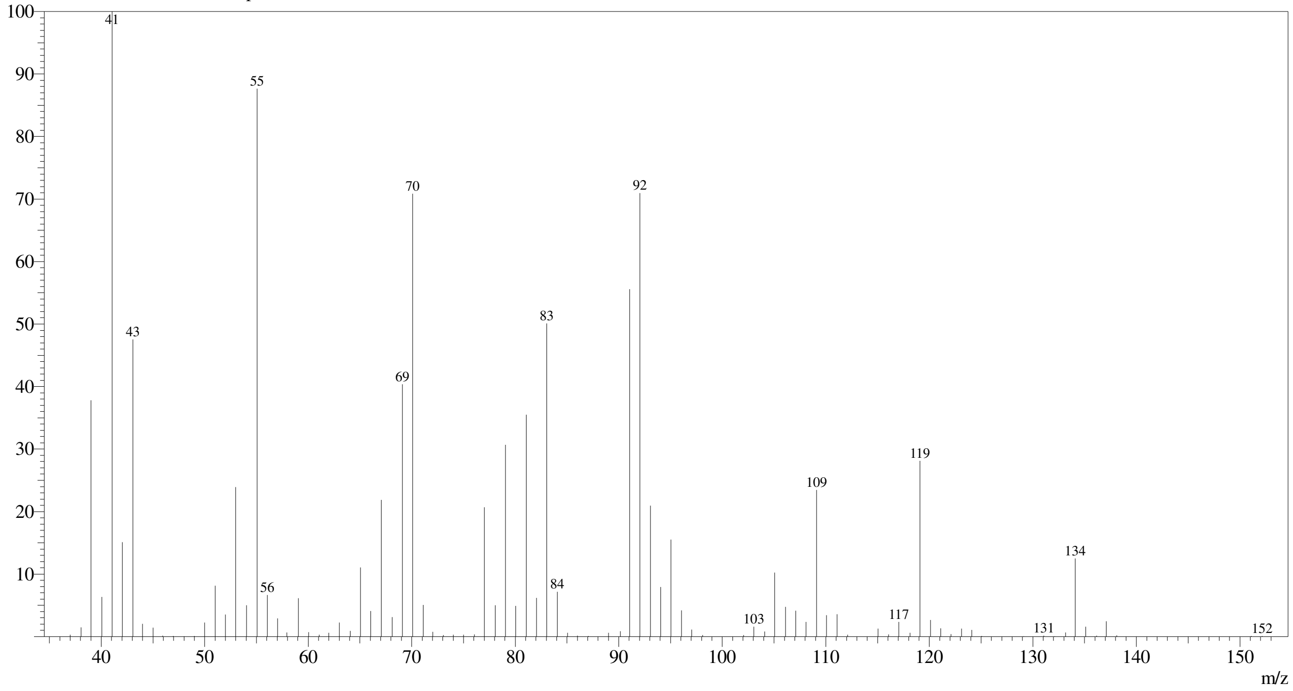

Mass Table
Peak#:8 R.Time:15.442(Scan#:1542)
MassPeaks:94
Group 1 - Event 1 Scan

#	m/z	Rel. Int.	#	m/z	Rel. Int.	#	m/z	Rel. Int.	#	m/z	Rel. Int.
1	37.00	0.32	25	61.05	0.32	49	85.05	0.64	73	115.05	1.30
2	38.05	1.48	26	62.00	0.60	50	86.00	0.13	74	116.05	0.36
3	39.00	37.80	27	63.00	2.23	51	87.05	0.08	75	117.05	2.38
4	40.05	6.37	28	64.05	0.92	52	89.00	0.63	76	118.15	0.59
5	41.05	100.00	29	65.05	11.05	53	90.15	0.87	77	119.10	28.14
6	42.05	15.09	30	66.05	4.07	54	91.05	55.59	78	120.10	2.64
7	43.05	47.55	31	67.05	21.86	55	92.05	70.93	79	121.10	1.34
8	44.00	2.07	32	68.10	3.13	56	93.05	20.97	80	122.10	0.43
9	45.00	1.45	33	69.10	40.36	57	94.05	7.94	81	123.10	1.27
10	45.95	0.15	34	70.10	70.86	58	95.05	15.51	82	124.10	1.07
11	46.95	0.11	35	71.10	5.06	59	96.05	4.22	83	125.05	0.13
12	48.00	0.03	36	72.05	0.77	60	97.05	1.15	84	129.10	0.03
13	49.00	0.12	37	73.05	0.23	61	98.10	0.27	85	131.05	0.14
14	50.00	2.25	38	74.00	0.29	62	102.05	0.24	86	132.10	0.03
15	51.00	8.15	39	75.00	0.29	63	103.05	1.56	87	133.15	0.66
16	52.00	3.54	40	76.05	0.34	64	104.10	0.83	88	134.10	12.48
17	53.00	23.95	41	77.00	20.68	65	105.05	10.26	89	135.10	1.58
18	54.05	5.04	42	78.05	5.04	66	106.10	4.75	90	136.15	0.17
19	55.05	87.67	43	79.05	30.68	67	107.10	4.13	91	137.10	2.44
20	56.05	6.66	44	80.05	4.94	68	108.10	2.36	92	138.10	0.19
21	57.05	2.94	45	81.05	35.52	69	109.10	23.44	93	151.10	0.10
22	57.95	0.68	46	82.05	6.22	70	110.05	3.45	94	152.15	0.11
23	59.05	6.15	47	83.05	50.13	71	111.10	3.59			
24	60.05	0.73	48	84.05	7.16	72	112.10	0.30			

Peak#:9 R.Time:15.660(Scan#:1568)
MassPeaks:88
RawMode:Averaged 15.650-15.667(1567-1569)
BG Mode:Calc. from Peak Group 1 - Event 1 Scan

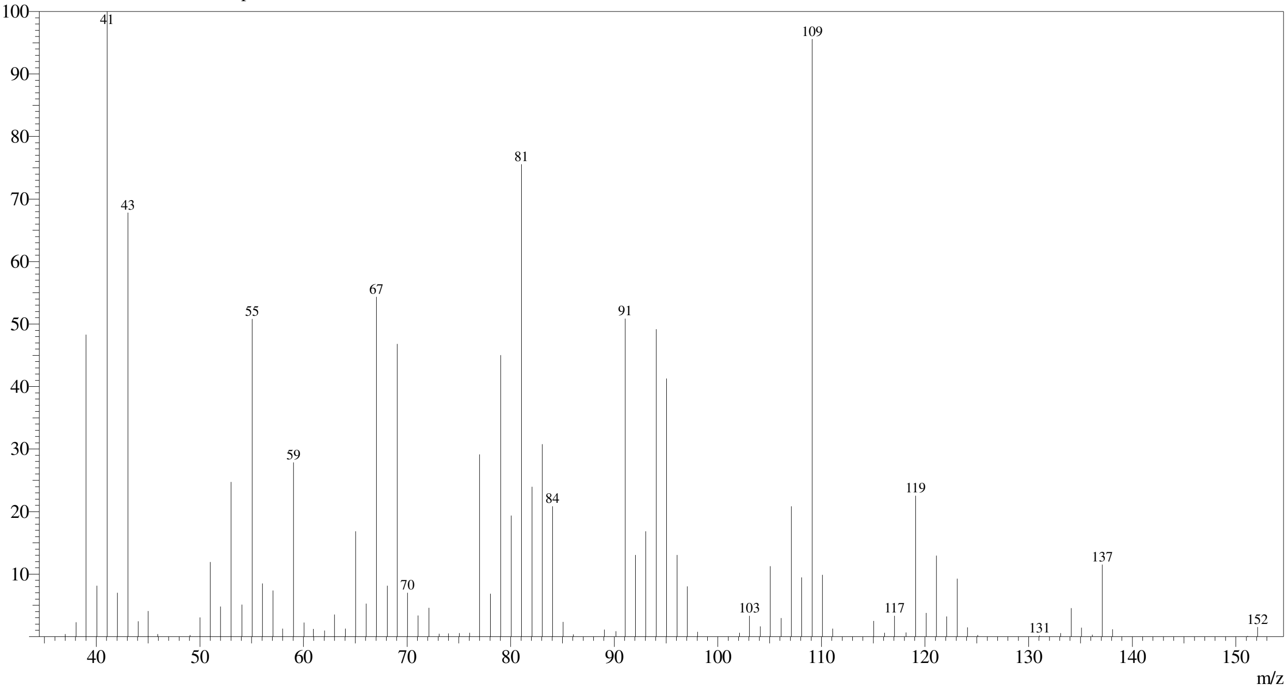

Mass Table
Peak#:9 R.Time:15.658(Scan#:1568)
MassPeaks:88
Group 1 - Event 1 Scan

#	m/z	Rel. Int.	#	m/z	Rel. Int.	#	m/z	Rel. Int.	#	m/z	Rel. Int.
1	37.00	0.42	23	60.95	1.24	45	83.05	30.77	67	110.10	9.88
2	38.05	2.29	24	62.05	0.95	46	84.05	20.87	68	111.10	1.26
3	39.00	48.31	25	63.00	3.55	47	85.05	2.36	69	115.05	2.52
4	40.05	8.15	26	64.05	1.31	48	86.05	0.34	70	116.10	0.61
5	41.05	100.00	27	65.05	16.84	49	89.05	1.11	71	117.05	3.35
6	42.05	6.99	28	66.05	5.27	50	90.15	0.85	72	118.15	0.68
7	43.05	67.83	29	67.05	54.36	51	91.05	50.88	73	119.10	22.54
8	44.05	2.46	30	68.10	8.15	52	92.05	13.08	74	120.10	3.78
9	45.00	4.11	31	69.05	46.81	53	93.05	16.85	75	121.10	12.96
10	45.95	0.40	32	70.05	7.03	54	94.05	49.17	76	122.10	3.21
11	49.05	0.24	33	71.05	3.40	55	95.05	41.27	77	123.10	9.30
12	50.00	3.09	34	72.10	4.62	56	96.05	13.06	78	124.10	1.50
13	51.00	11.93	35	73.10	0.48	57	97.05	8.02	79	125.10	0.26
14	52.00	4.83	36	74.00	0.52	58	98.05	0.74	80	131.05	0.16
15	53.00	24.76	37	75.05	0.58	59	102.10	0.60	81	133.10	0.59
16	54.05	5.10	38	76.05	0.61	60	103.05	3.31	82	134.10	4.57
17	55.05	50.84	39	77.00	29.15	61	104.10	1.65	83	135.10	1.44
18	56.05	8.51	40	78.05	6.84	62	105.05	11.28	84	136.15	0.32
19	57.05	7.36	41	79.05	45.02	63	106.10	2.95	85	137.10	11.51
20	58.00	1.30	42	80.05	19.38	64	107.10	20.86	86	138.10	1.16
21	59.05	27.89	43	81.05	75.57	65	108.10	9.50	87	151.15	0.08
22	60.05	2.26	44	82.05	24.00	66	109.10	95.59	88	152.10	1.53

Peak#:10 R.Time:16.349(Scan#:1651)
MassPeaks:83
RawMode:Averaged 16.342-16.358(1650-1652)
BG Mode:Calc. from Peak Group 1 - Event 1 Scan

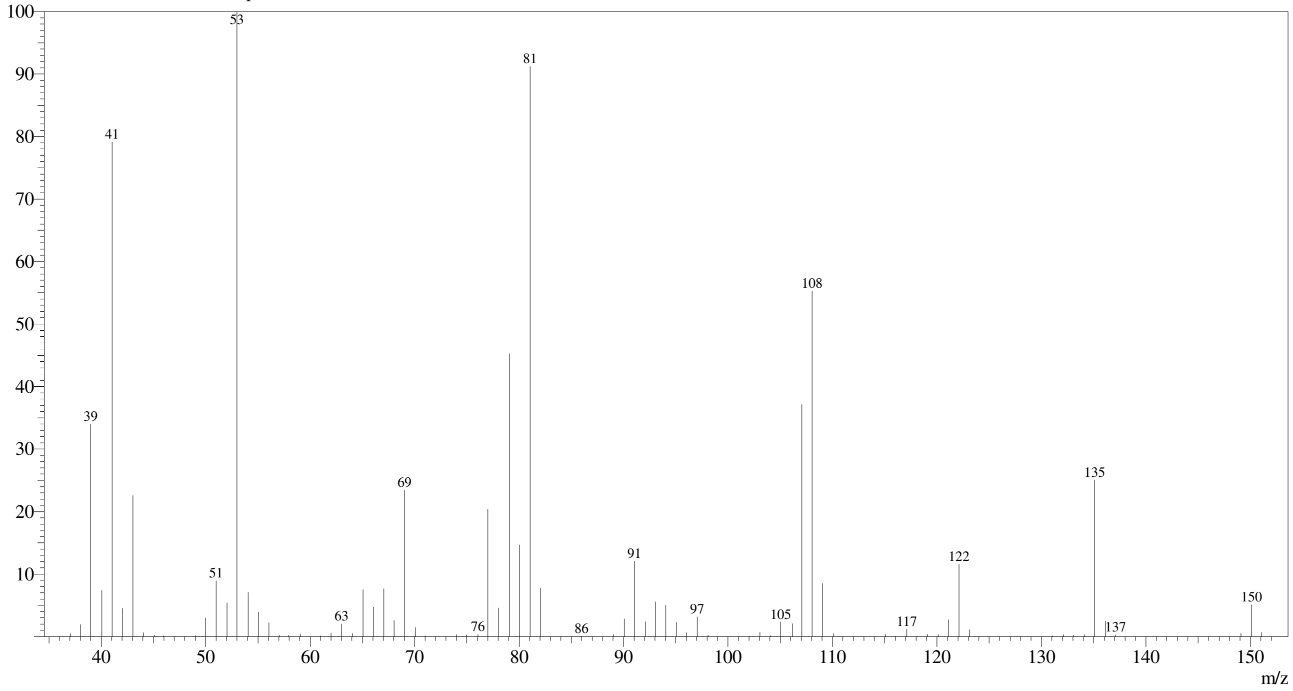

Mass Table
Peak#:10 R.Time:16.350(Scan#:1651)
MassPeaks:83
Group 1 - Event 1 Scan

#	m/z	Rel. Int.	#	m/z	Rel. Int.	#	m/z	Rel. Int.	#	m/z	Rel. Int.
1	37.05	0.49	22	61.00	0.15	43	86.00	0.13	64	115.05	0.39
2	38.05	1.94	23	62.00	0.62	44	89.05	0.37	65	116.05	0.11
3	39.00	34.03	24	63.00	2.03	45	90.05	2.86	66	117.10	1.24
4	40.05	7.43	25	64.05	0.58	46	91.05	12.09	67	118.10	0.13
5	41.05	79.16	26	65.05	7.51	47	92.10	2.40	68	119.10	0.42
6	42.05	4.58	27	66.05	4.75	48	93.05	5.61	69	120.10	0.36
7	43.05	22.59	28	67.05	7.66	49	94.05	5.10	70	121.10	2.70
8	44.05	0.66	29	68.05	2.63	50	95.05	2.29	71	122.10	11.59
9	45.10	0.26	30	69.05	23.42	51	96.05	0.65	72	123.10	1.10
10	45.95	0.13	31	70.10	1.50	52	97.05	3.18	73	124.10	0.07
11	49.05	0.20	32	71.00	0.13	53	98.10	0.26	74	131.05	0.14
12	50.00	3.03	33	74.00	0.36	54	102.05	0.11	75	132.10	0.37
13	51.00	8.95	34	75.00	0.29	55	103.05	0.70	76	133.05	0.24
14	52.05	5.43	35	76.05	0.36	56	104.10	0.19	77	134.15	0.34
15	53.00	100.00	36	77.00	20.40	57	105.05	2.37	78	135.10	25.03
16	54.05	7.11	37	78.05	4.62	58	106.15	2.12	79	136.10	2.51
17	55.05	3.93	38	79.05	45.27	59	107.05	37.15	80	137.10	0.31
18	56.05	2.25	39	80.05	14.73	60	108.05	55.33	81	149.10	0.55
19	57.05	0.27	40	81.05	91.24	61	109.05	8.49	82	150.10	5.15
20	57.95	0.25	41	82.05	7.81	62	110.10	0.52	83	151.10	0.71
21	59.05	0.45	42	84.05	0.04	63	111.10	0.05			

Peak#:11 R.Time:16.840(Scan#:1710)
MassPeaks:63
RawMode:Averaged 16.833-16.850(1709-1711)
BG Mode:Calc. from Peak Group 1 - Event 1 Scan

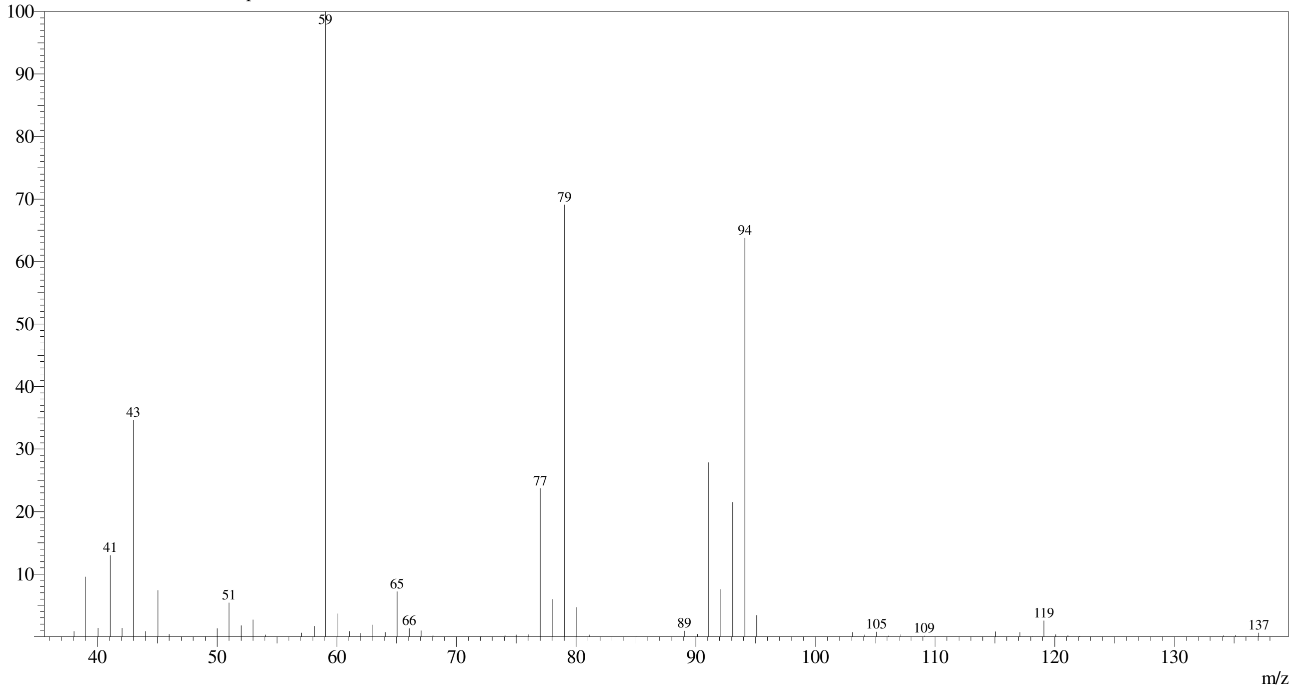

Mass Table
Peak#:11 R.Time:16.842(Scan#:1710)
MassPeaks:63
Group 1 - Event 1 Scan

#	m/z	Rel. Int.	#	m/z	Rel. Int.	#	m/z	Rel. Int.	#	m/z	Rel. Int.
1	38.05	0.88	17	58.15	1.68	33	77.00	23.73	49	105.10	0.76
2	39.00	9.60	18	59.05	100.00	34	78.05	6.01	50	106.10	0.13
3	40.05	1.39	19	60.10	3.70	35	79.05	69.09	51	107.10	0.37
4	41.05	13.01	20	61.05	0.85	36	80.05	4.70	52	109.10	0.14
5	42.05	1.39	21	62.00	0.57	37	81.10	0.33	53	115.05	0.82
6	43.00	34.69	22	63.00	1.88	38	85.05	0.01	54	116.10	0.07
7	44.00	0.88	23	64.05	0.70	39	89.05	0.92	55	117.10	0.73
8	45.05	7.45	24	65.05	7.23	40	90.15	0.43	56	118.15	0.07
9	46.00	0.42	25	66.05	1.31	41	91.05	27.88	57	119.10	2.55
10	49.00	0.07	26	67.05	0.97	42	92.05	7.56	58	120.10	0.37
11	50.00	1.31	27	68.05	0.18	43	93.10	21.50	59	121.10	0.19
12	51.00	5.42	28	71.05	0.03	44	94.10	63.80	60	122.10	0.11
13	52.00	1.79	29	72.05	0.12	45	95.10	3.43	61	134.10	0.21
14	53.00	2.74	30	74.05	0.24	46	102.05	0.07	62	135.10	0.23
15	54.05	0.29	31	75.00	0.30	47	103.10	0.71	63	137.05	0.62
16	57.05	0.60	32	76.05	0.38	48	104.10	0.33			

Peak#:12 R.Time:17.892(Scan#:1836)
MassPeaks:82
RawMode:Averaged 17.883-17.900(1835-1837)
BG Mode:Calc. from Peak Group 1 - Event 1 Scan

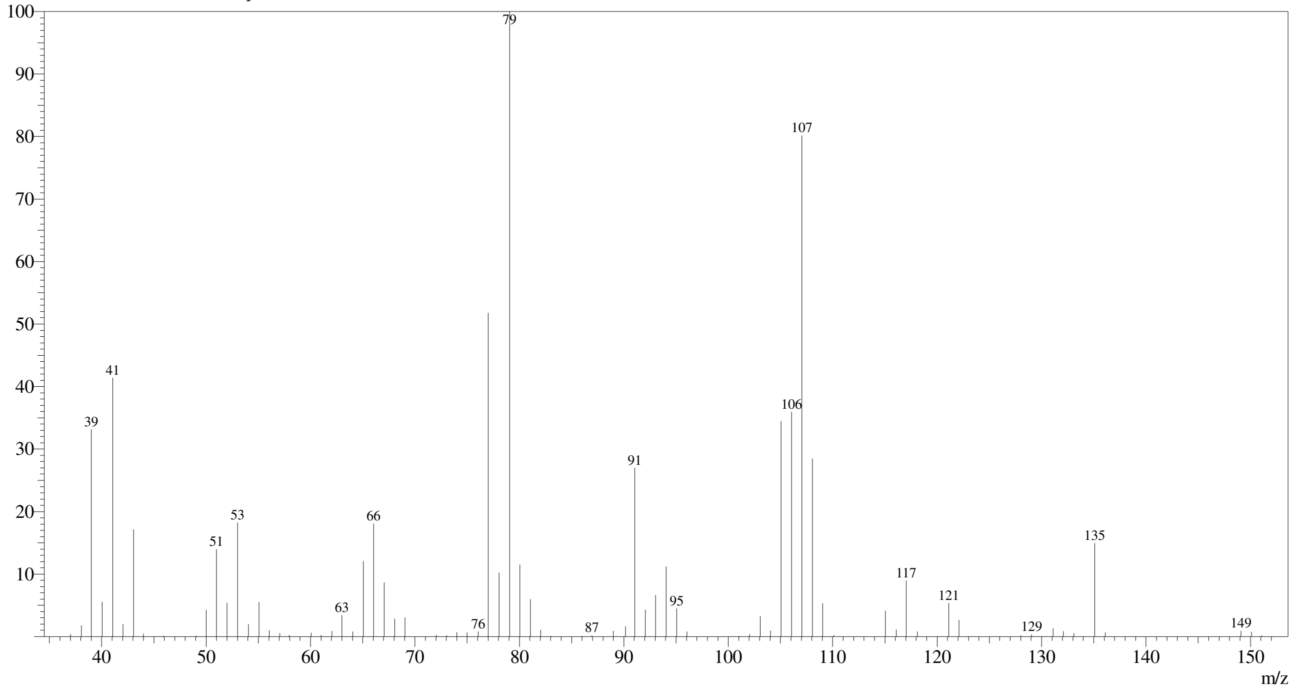

Mass Table
Peak#:12 R.Time:17.892(Scan#:1836)
MassPeaks:82
Group 1 - Event 1 Scan

#	m/z	Rel. Int.	#	m/z	Rel. Int.	#	m/z	Rel. Int.	#	m/z	Rel. Int.
1	37.00	0.42	22	61.00	0.23	43	86.00	0.11	64	116.10	1.11
2	38.05	1.79	23	62.05	0.92	44	86.95	0.20	65	117.05	8.98
3	39.00	33.16	24	63.00	3.45	45	89.00	0.92	66	118.10	0.83
4	40.05	5.59	25	64.05	0.84	46	90.15	1.66	67	120.10	0.06
5	41.05	41.37	26	65.05	12.08	47	91.05	27.01	68	121.10	5.39
6	42.05	2.00	27	66.05	18.10	48	92.05	4.31	69	122.10	2.65
7	43.05	17.19	28	67.05	8.67	49	93.05	6.68	70	123.10	0.03
8	44.00	0.46	29	68.05	2.85	50	94.05	11.22	71	128.05	0.19
9	45.00	0.07	30	69.05	3.09	51	95.05	4.51	72	129.05	0.35
10	45.90	0.15	31	72.05	0.29	52	96.05	0.81	73	130.10	0.07
11	49.05	0.16	32	73.05	0.19	53	101.05	0.11	74	131.10	1.33
12	50.00	4.30	33	74.00	0.73	54	102.05	0.43	75	132.10	0.85
13	51.00	13.99	34	75.00	0.68	55	103.05	3.26	76	133.10	0.55
14	52.00	5.44	35	76.05	0.82	56	104.05	1.00	77	134.15	0.02
15	53.00	18.22	36	77.00	51.81	57	105.05	34.46	78	135.10	14.97
16	54.05	1.99	37	78.05	10.24	58	106.05	35.97	79	136.10	0.67
17	55.05	5.54	38	79.05	100.00	59	107.05	80.19	80	149.10	0.94
18	56.05	1.00	39	80.05	11.50	60	108.05	28.50	81	150.10	0.76
19	57.05	0.54	40	81.05	5.99	61	109.05	5.34	82	151.10	0.23
20	57.95	0.23	41	82.05	1.09	62	110.10	0.24			
21	60.05	0.61	42	83.05	0.10	63	115.05	4.13			

Peak#:13 R.Time:17.990(Scan#:1848)
MassPeaks:91
RawMode:Averaged 17.983-18.000(1847-1849)
BG Mode:Calc. from Peak Group 1 - Event 1 Scan

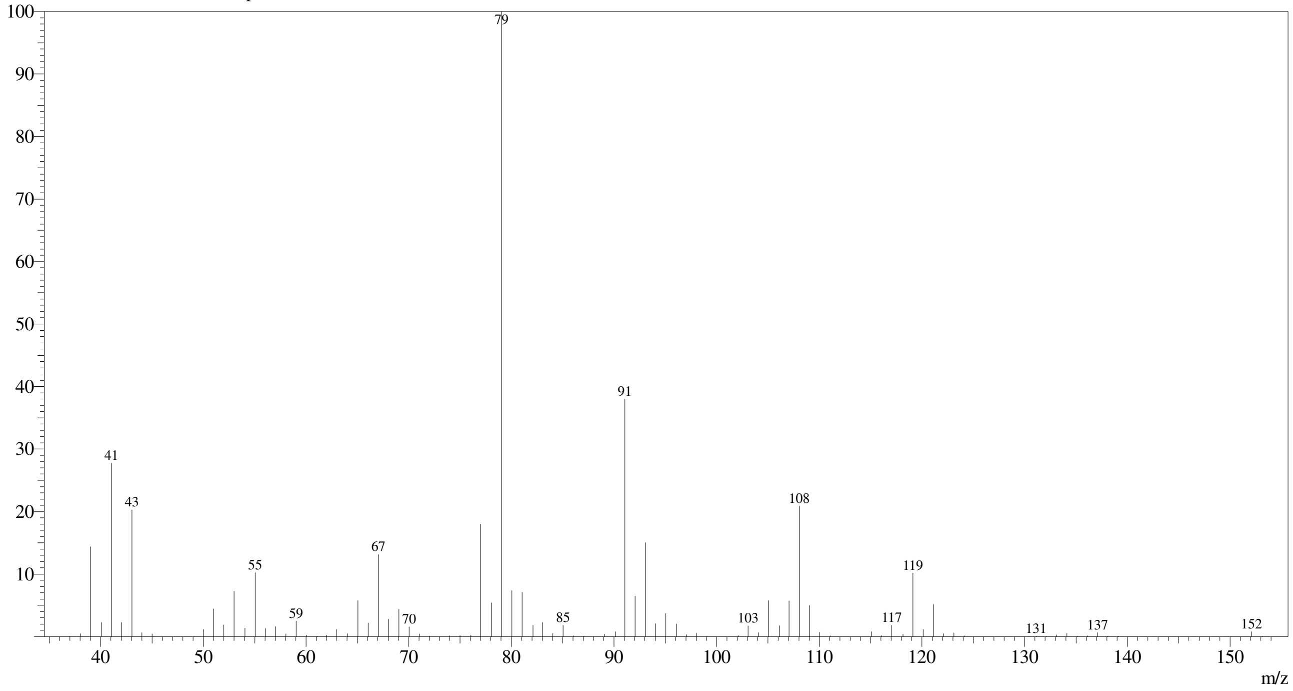

Mass Table
Peak#:13 R.Time:17.992(Scan#:1848)
MassPeaks:91
Group 1 - Event 1 Scan

#	m/z	Rel. Int.	#	m/z	Rel. Int.	#	m/z	Rel. Int.	#	m/z	Rel. Int.
1	37.00	0.12	24	62.00	0.28	47	86.05	0.15	70	115.05	0.81
2	38.05	0.52	25	63.00	1.16	48	87.05	0.08	71	116.05	0.20
3	39.00	14.37	26	64.05	0.52	49	89.05	0.40	72	117.05	1.83
4	40.05	2.30	27	65.05	5.78	50	90.15	0.83	73	118.15	0.39
5	41.05	27.76	28	66.05	2.22	51	91.05	37.99	74	119.10	10.18
6	42.05	2.32	29	67.05	13.18	52	92.05	6.51	75	120.10	1.19
7	43.05	20.27	30	68.05	2.84	53	93.05	15.05	76	121.10	5.15
8	44.00	0.67	31	69.05	4.39	54	94.05	2.10	77	122.10	0.51
9	45.00	0.44	32	70.05	1.57	55	95.05	3.76	78	123.10	0.61
10	45.90	0.06	33	71.05	0.48	56	96.10	2.03	79	124.10	0.13
11	49.05	0.05	34	72.05	0.10	57	97.05	0.35	80	131.10	0.09
12	50.00	1.17	35	74.00	0.17	58	98.05	0.54	81	132.10	0.04
13	51.00	4.43	36	75.00	0.20	59	99.05	0.03	82	133.10	0.30
14	52.00	1.88	37	76.05	0.28	60	102.10	0.19	83	134.10	0.56
15	53.00	7.30	38	77.00	18.05	61	103.05	1.76	84	135.10	0.11
16	54.05	1.36	39	78.05	5.46	62	104.05	0.69	85	136.10	0.16
17	55.05	10.24	40	79.05	100.00	63	105.05	5.81	86	137.10	0.65
18	56.05	1.31	41	80.05	7.38	64	106.10	1.82	87	138.10	0.12
19	57.05	1.63	42	81.05	7.10	65	107.05	5.74	88	139.10	0.09
20	58.05	0.44	43	82.10	1.84	66	108.05	20.90	89	150.05	0.02
21	59.05	2.49	44	83.05	2.30	67	109.05	5.01	90	152.10	0.82
22	60.05	0.25	45	84.05	0.57	68	110.05	0.74	91	153.15	0.11
23	61.05	0.09	46	85.05	1.84	69	111.10	0.14			

Peak#:14 R.Time:18.406(Scan#:1898)
MassPeaks:85
RawMode:Averaged 18.400-18.417(1897-1899)
BG Mode:Calc. from Peak Group 1 - Event 1 Scan

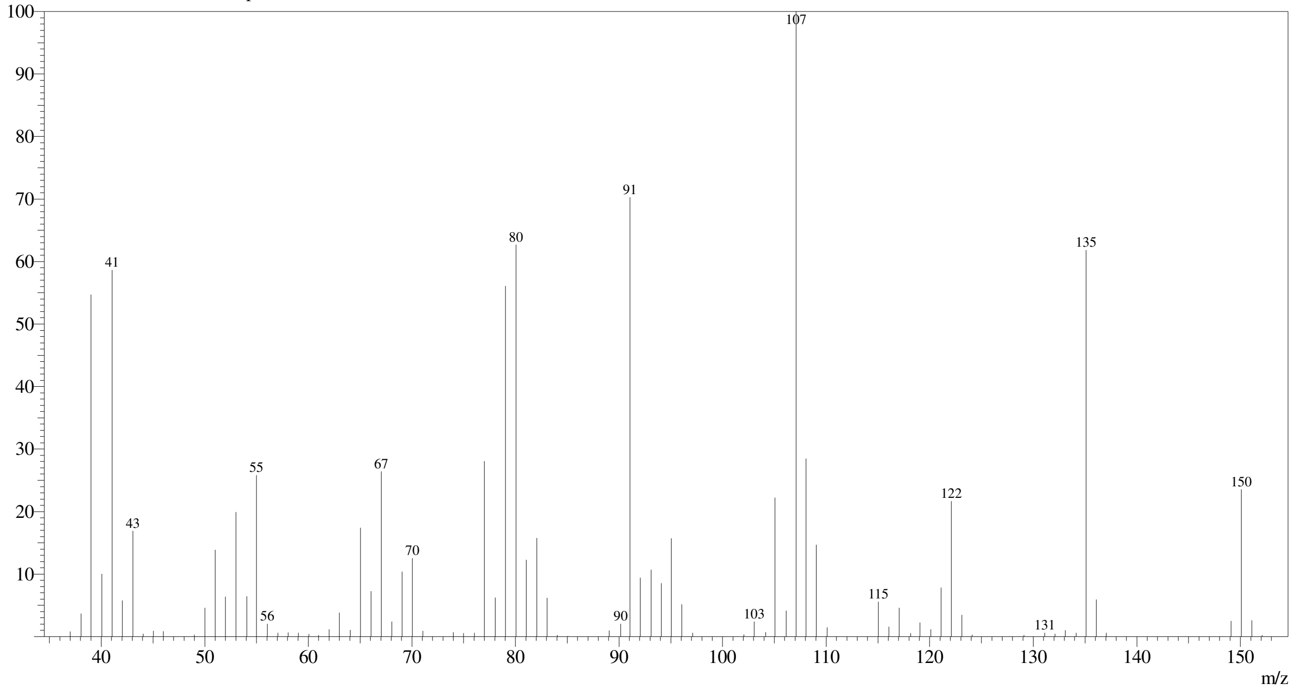

Mass Table
Peak#:14 R.Time:18.408(Scan#:1898)
MassPeaks:85
Group 1 - Event 1 Scan

#	m/z	Rel. Int.	#	m/z	Rel. Int.	#	m/z	Rel. Int.	#	m/z	Rel. Int.
1	37.00	0.80	23	61.00	0.28	45	85.05	0.08	67	118.15	0.55
2	38.05	3.69	24	62.00	1.17	46	89.05	1.00	68	119.05	2.26
3	39.00	54.71	25	63.00	3.82	47	90.15	2.02	69	120.10	1.20
4	40.05	10.06	26	64.05	1.08	48	91.05	70.27	70	121.10	7.83
5	41.05	58.66	27	65.05	17.42	49	92.05	9.42	71	122.10	21.67
6	42.05	5.79	28	66.05	7.28	50	93.10	10.73	72	123.10	3.47
7	43.05	16.90	29	67.05	26.44	51	94.10	8.55	73	124.10	0.32
8	44.05	0.46	30	68.05	2.43	52	95.05	15.75	74	129.10	0.24
9	45.05	0.91	31	69.05	10.41	53	96.05	5.20	75	131.10	0.62
10	46.00	0.89	32	70.05	12.56	54	97.10	0.60	76	132.10	0.48
11	49.00	0.32	33	71.05	0.91	55	102.05	0.33	77	133.10	1.04
12	50.00	4.61	34	74.00	0.69	56	103.05	2.43	78	134.15	0.62
13	51.00	13.86	35	75.00	0.55	57	104.15	0.70	79	135.10	61.84
14	52.00	6.42	36	76.05	0.60	58	105.05	22.22	80	136.10	5.93
15	53.00	19.93	37	77.00	28.09	59	106.15	4.15	81	137.05	0.60
16	54.05	6.44	38	78.05	6.27	60	107.10	100.00	82	149.10	2.51
17	55.00	25.84	39	79.05	56.07	61	108.05	28.51	83	150.10	23.58
18	56.05	2.07	40	80.05	62.70	62	109.05	14.68	84	151.10	2.64
19	57.05	0.63	41	81.05	12.31	63	110.10	1.50	85	152.10	0.26
20	58.05	0.66	42	82.05	15.76	64	115.05	5.59			
21	59.05	0.63	43	83.05	6.18	65	116.05	1.60			
22	60.05	0.40	44	84.05	0.25	66	117.05	4.60			

Peak#:15 R.Time:24.092(Scan#:2580)
MassPeaks:80
RawMode:Averaged 24.083-24.100(2579-2581)
BG Mode:Calc. from Peak Group 1 - Event 1 Scan

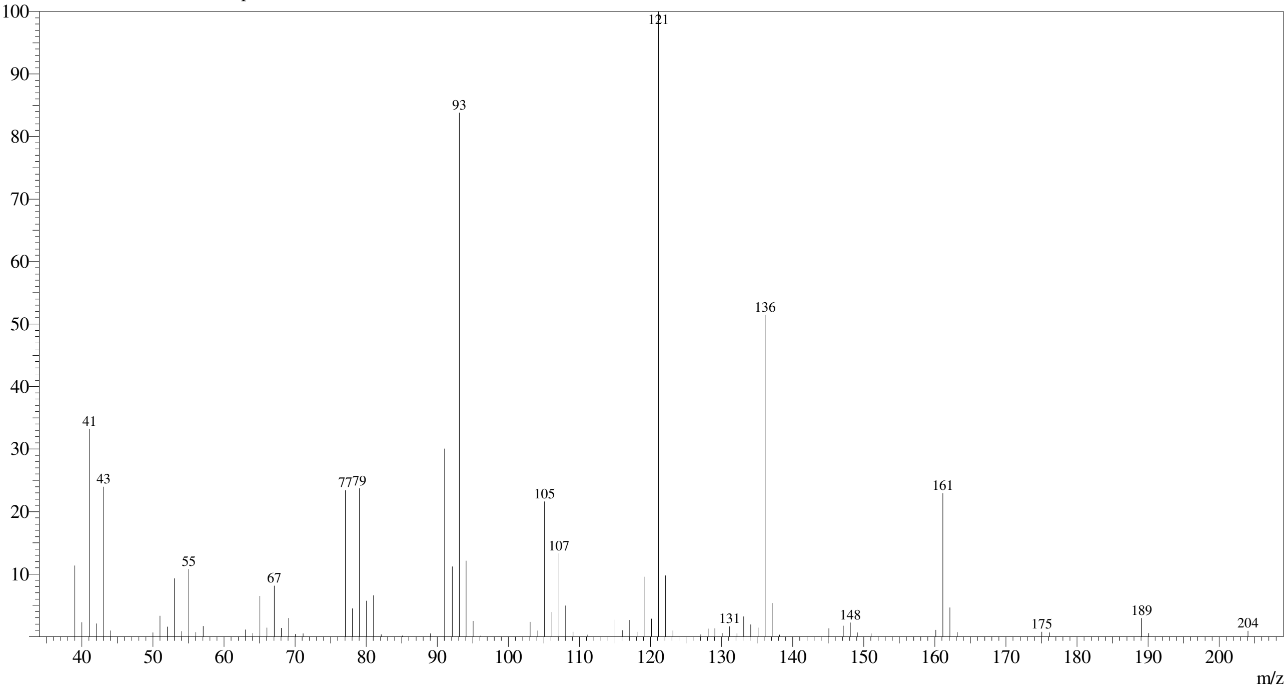

Mass Table
Peak#:15 R.Time:24.092(Scan#:2580)
MassPeaks:80
Group 1 - Event 1 Scan

#	m/z	Rel. Int.	#	m/z	Rel. Int.	#	m/z	Rel. Int.	#	m/z	Rel. Int.
1	39.00	11.38	21	69.10	2.95	41	106.10	3.92	61	133.10	3.25
2	40.00	2.29	22	70.05	0.42	42	107.10	13.33	62	134.10	1.95
3	41.05	33.23	23	71.10	0.53	43	108.05	4.98	63	135.15	1.45
4	42.05	2.10	24	77.05	23.40	44	109.10	0.77	64	136.10	51.49
5	43.05	23.99	25	78.05	4.53	45	111.15	0.32	65	137.10	5.39
6	44.05	0.99	26	79.05	23.72	46	115.00	2.73	66	138.15	0.31
7	50.00	0.66	27	80.05	5.71	47	116.05	1.02	67	145.10	1.32
8	51.00	3.35	28	81.05	6.59	48	117.05	2.67	68	147.10	1.76
9	52.00	1.59	29	82.10	0.38	49	118.10	0.77	69	148.10	2.23
10	53.00	9.31	30	85.10	0.15	50	119.10	9.59	70	149.10	0.66
11	54.05	0.85	31	89.05	0.53	51	120.15	2.88	71	151.05	0.53
12	55.05	10.80	32	91.05	30.10	52	121.10	100.00	72	160.15	1.07
13	56.05	0.70	33	92.10	11.24	53	122.10	9.79	73	161.10	22.96
14	57.05	1.69	34	93.10	83.80	54	123.15	0.98	74	162.10	4.64
15	63.00	1.11	35	94.05	12.16	55	127.05	0.34	75	163.15	0.71
16	64.05	0.56	36	95.05	2.52	56	128.10	1.28	76	175.05	0.77
17	65.05	6.52	37	96.10	0.16	57	129.05	1.36	77	176.10	0.65
18	66.05	1.44	38	103.05	2.35	58	130.10	0.58	78	189.10	2.98
19	67.05	8.13	39	104.15	0.97	59	131.10	1.63	79	190.10	0.58
20	68.05	1.38	40	105.10	21.62	60	132.15	0.53	80	204.05	0.93

Peak#:16 R.Time:30.180(Scan#:3311)
MassPeaks:97
RawMode:Averaged 30.175-30.192(3310-3312)
BG Mode:Calc. from Peak Group 1 - Event 1 Scan

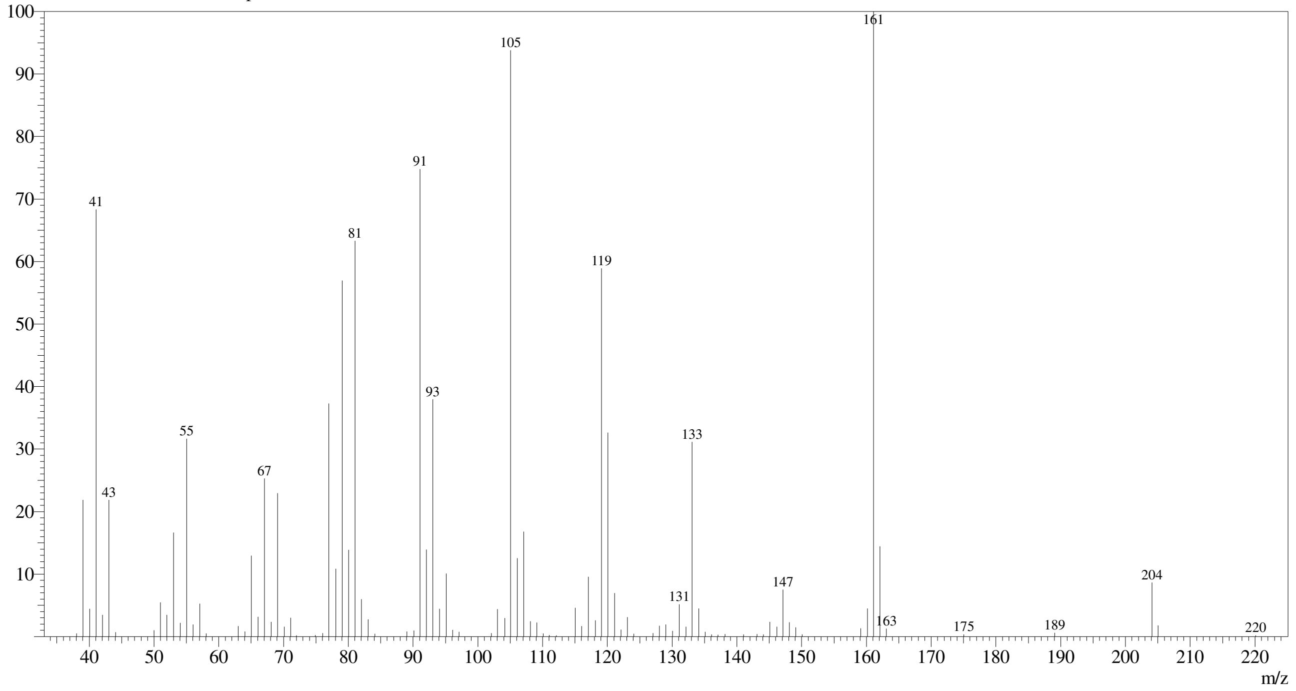

Mass Table
Peak#:16 R.Time:30.183(Scan#:3311)
MassPeaks:97
Group 1 - Event 1 Scan

#	m/z	Rel. Int.	#	m/z	Rel. Int.	#	m/z	Rel. Int.	#	m/z	Rel. Int.
1	38.05	0.50	26	72.00	0.23	51	107.10	16.78	76	136.10	0.37
2	39.05	21.88	27	74.90	0.26	52	108.10	2.46	77	137.10	0.32
3	40.05	4.46	28	76.05	0.56	53	109.10	2.23	78	138.15	0.41
4	41.05	68.33	29	77.00	37.32	54	110.10	0.51	79	141.05	0.38
5	42.05	3.49	30	78.05	10.86	55	111.05	0.26	80	143.10	0.42
6	43.05	21.89	31	79.05	56.95	56	112.10	0.23	81	144.10	0.37
7	44.05	0.72	32	80.05	13.88	57	115.05	4.62	82	145.10	2.34
8	50.00	1.04	33	81.05	63.34	58	116.05	1.68	83	146.15	1.59
9	51.00	5.47	34	82.05	6.00	59	117.05	9.60	84	147.10	7.53
10	52.00	3.46	35	83.05	2.79	60	118.15	2.61	85	148.10	2.30
11	53.00	16.65	36	84.10	0.46	61	119.10	58.89	86	149.10	1.47
12	54.05	2.19	37	89.05	0.83	62	120.10	32.66	87	150.10	0.36
13	55.05	31.66	38	90.15	0.96	63	121.10	6.96	88	159.10	1.34
14	56.05	1.93	39	91.05	74.78	64	122.10	1.10	89	160.15	4.52
15	57.05	5.30	40	92.05	13.96	65	123.10	3.13	90	161.10	100.00
16	58.05	0.50	41	93.05	37.98	66	124.10	0.45	91	162.10	14.43
17	63.00	1.67	42	94.10	4.47	67	127.05	0.56	92	163.10	1.26
18	64.05	0.83	43	95.10	10.09	68	128.05	1.77	93	175.05	0.33
19	65.05	12.98	44	96.10	1.06	69	129.05	1.94	94	189.05	0.60
20	66.05	3.20	45	97.10	0.79	70	130.05	0.93	95	204.10	8.64
21	67.05	25.30	46	102.10	0.56	71	131.10	5.17	96	205.05	1.80
22	68.10	2.34	47	103.05	4.42	72	132.15	1.59	97	220.10	0.18
23	69.10	22.97	48	104.15	3.00	73	133.10	31.15			
24	70.10	1.59	49	105.05	93.81	74	134.10	4.51			
25	71.10	3.05	50	106.10	12.57	75	135.10	0.77			

Peak#:17   R.Time:34.049(Scan#:3775)
MassPeaks:147
RawMode:Averaged 34.042-34.058(3774-3776)
BG Mode:Calc. from Peak Group 1 - Event 1 Scan

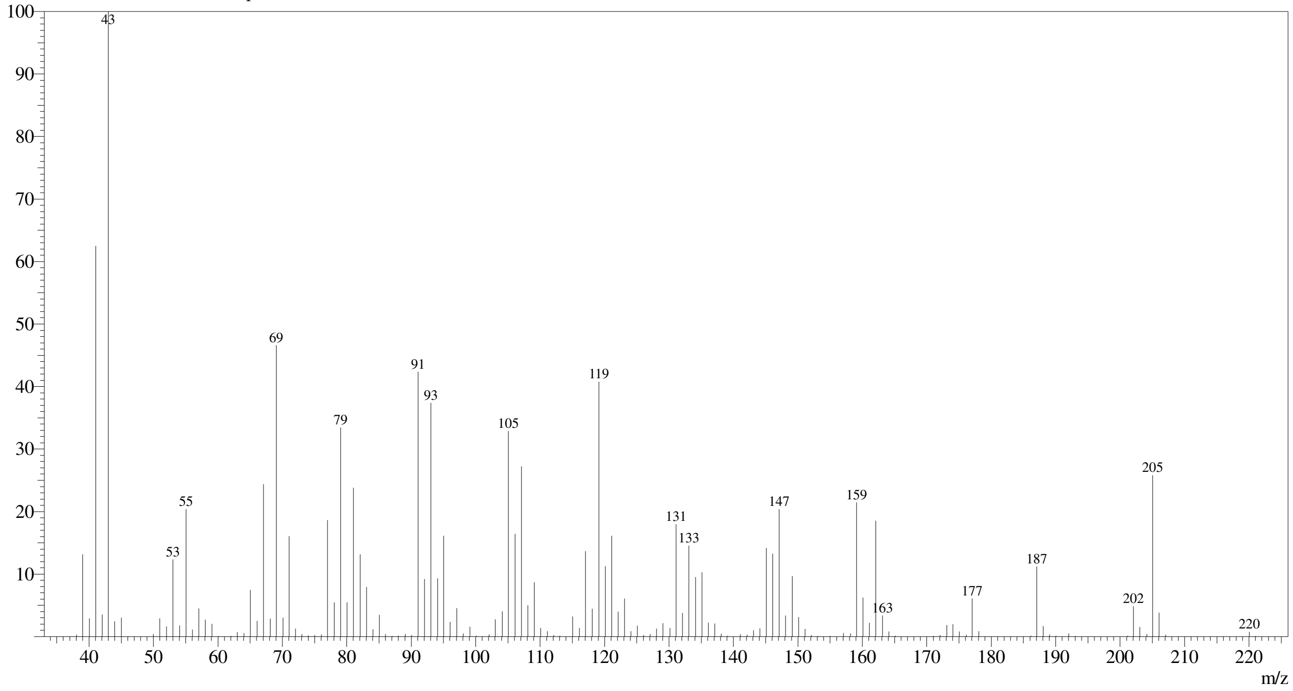

Mass Table
Peak#:17   R.Time:34.050(Scan#:3775)
MassPeaks:147
Group 1 - Event 1 Scan

#	m/z	Rel. Int.	#	m/z	Rel. Int.	#	m/z	Rel. Int.	#	m/z	Rel. Int.
1	38.05	0.33	38	79.05	33.47	75	117.05	13.67	112	157.05	0.54
2	39.00	13.15	39	80.05	5.47	76	118.10	4.45	113	158.15	0.53
3	40.05	2.90	40	81.05	23.83	77	119.10	40.78	114	159.10	21.45
4	41.05	62.49	41	82.05	13.17	78	120.10	11.28	115	160.10	6.27
5	42.05	3.51	42	83.05	7.96	79	121.10	16.12	116	161.10	2.20
6	43.00	100.00	43	84.05	1.17	80	122.10	3.99	117	162.10	18.55
7	44.00	2.47	44	85.05	3.50	81	123.10	6.10	118	163.10	3.38
8	45.00	3.04	45	86.00	0.40	82	124.10	0.89	119	164.10	0.79
9	46.10	0.03	46	87.05	0.12	83	125.10	1.72	120	165.10	0.16
10	50.00	0.40	47	88.00	0.14	84	126.05	0.26	121	171.05	0.11
11	51.00	2.94	48	89.05	0.42	85	127.05	0.42	122	172.05	0.23
12	52.00	1.66	49	90.05	0.28	86	128.05	1.29	123	173.10	1.83
13	53.00	12.33	50	91.05	42.36	87	129.05	2.14	124	174.05	1.99
14	54.05	1.77	51	92.05	9.21	88	130.15	1.39	125	175.05	0.84
15	55.05	20.41	52	93.05	37.39	89	131.10	17.96	126	176.15	0.29
16	56.05	1.12	53	94.10	9.35	90	132.10	3.79	127	177.05	6.07
17	57.05	4.52	54	95.05	16.13	91	133.10	14.57	128	178.05	0.86
18	58.05	2.74	55	96.05	2.34	92	134.10	9.54	129	179.10	0.05
19	59.05	2.03	56	97.05	4.53	93	135.10	10.30	130	185.05	0.03
20	60.10	0.12	57	98.05	0.49	94	136.10	2.27	131	186.15	0.15
21	62.05	0.11	58	99.10	1.57	95	137.10	2.10	132	187.05	11.24
22	63.00	0.71	59	100.10	0.14	96	138.10	0.48	133	188.05	1.68
23	64.05	0.55	60	101.10	0.03	97	139.10	0.16	134	189.05	0.35
24	65.05	7.46	61	102.10	0.37	98	141.05	0.33	135	190.05	0.16
25	66.10	2.50	62	103.05	2.76	99	142.10	0.33	136	192.05	0.49
26	67.05	24.37	63	104.10	4.05	100	143.10	1.05	137	193.00	0.09
27	68.10	2.87	64	105.05	32.89	101	144.10	1.32	138	201.15	0.14
28	69.05	46.61	65	106.10	16.46	102	145.10	14.19	139	202.05	4.85
29	70.10	3.02	66	107.10	27.25	103	146.10	13.24	140	203.05	1.52
30	71.05	16.08	67	108.10	5.04	104	147.10	20.38	141	204.15	0.42
31	72.05	1.30	68	109.10	8.69	105	148.10	3.36	142	205.05	25.80
32	73.05	0.40	69	110.10	1.40	106	149.10	9.67	143	206.05	3.82
33	74.00	0.18	70	111.10	0.85	107	150.10	3.15	144	207.10	0.32
34	75.00	0.28	71	112.10	0.28	108	151.10	1.22	145	216.05	0.03
35	76.05	0.35	72	113.05	0.17	109	152.10	0.26	146	220.05	0.78
36	77.00	18.63	73	115.05	3.24	110	153.15	0.10	147	221.05	0.12
37	78.05	5.50	74	116.10	1.40	111	155.00	0.10			

Peak#:18 R.Time:34.158(Scan#:3788)
MassPeaks:80
RawMode:Averaged 34.150-34.167(3787-3789)
BG Mode:Calc. from Peak Group 1 - Event 1 Scan

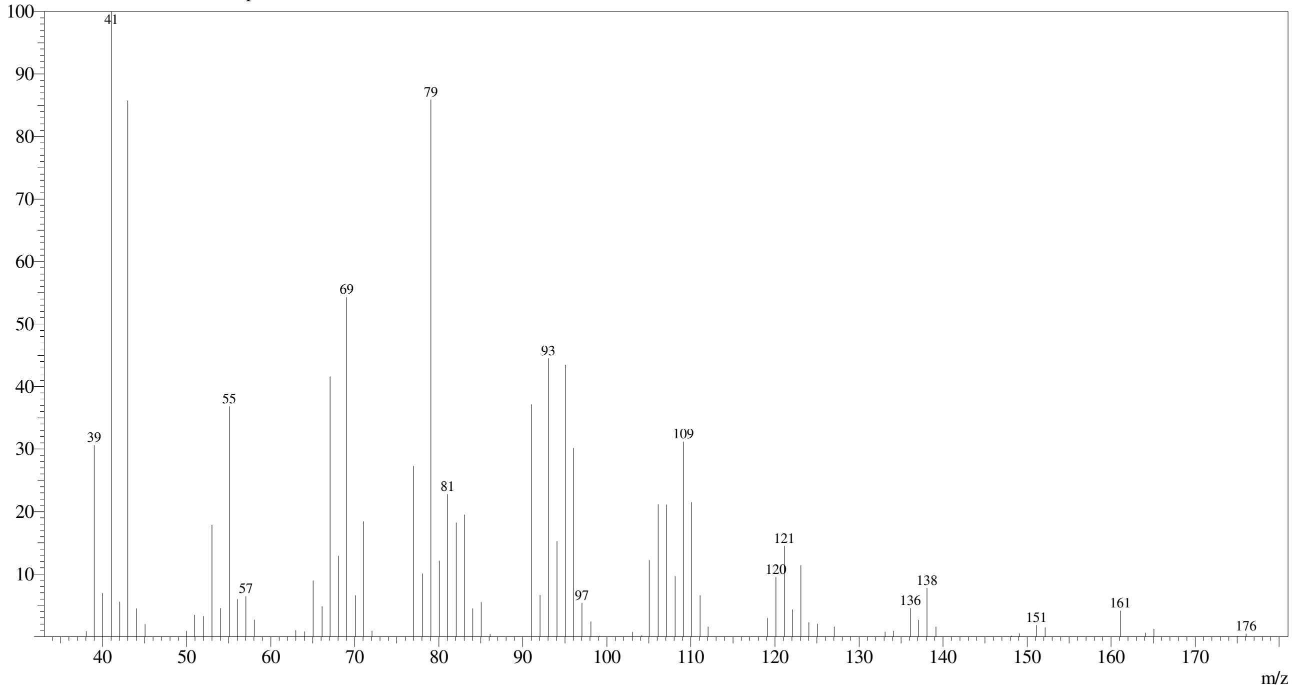

Mass Table
Peak#:18 R.Time:34.158(Scan#:3788)
MassPeaks:80
Group 1 - Event 1 Scan

#	m/z	Rel. Int.	#	m/z	Rel. Int.	#	m/z	Rel. Int.	#	m/z	Rel. Int.
1	38.05	0.86	21	66.10	4.87	41	94.05	15.24	61	122.10	4.38
2	39.00	30.63	22	67.05	41.61	42	95.05	43.47	62	123.10	11.43
3	40.00	6.95	23	68.05	12.89	43	96.05	30.16	63	124.05	2.32
4	41.05	100.00	24	69.05	54.32	44	97.05	5.40	64	125.05	2.07
5	42.05	5.58	25	70.10	6.61	45	98.10	2.41	65	127.05	1.60
6	43.00	85.75	26	71.05	18.46	46	99.10	0.09	66	133.10	0.78
7	44.00	4.52	27	72.05	0.95	47	103.05	0.79	67	134.10	0.95
8	45.05	2.00	28	77.00	27.29	48	104.15	0.21	68	136.10	4.55
9	49.95	0.91	29	78.05	10.07	49	105.05	12.23	69	137.10	2.66
10	50.95	3.51	30	79.05	85.93	50	106.10	21.15	70	138.10	7.79
11	52.00	3.29	31	80.05	12.14	51	107.10	21.13	71	139.15	1.61
12	53.00	17.86	32	81.05	22.81	52	108.10	9.69	72	148.15	0.13
13	54.05	4.54	33	82.05	18.23	53	109.10	31.22	73	149.10	0.50
14	55.05	36.84	34	83.05	19.54	54	110.10	21.52	74	151.10	1.87
15	56.05	5.99	35	84.05	4.53	55	111.10	6.59	75	152.15	1.50
16	57.05	6.44	36	85.05	5.54	56	112.05	1.61	76	161.10	4.14
17	58.05	2.72	37	86.10	0.42	57	116.10	0.01	77	164.05	0.59
18	63.00	1.04	38	91.05	37.15	58	119.10	2.98	78	165.10	1.24
19	64.05	0.83	39	92.05	6.64	59	120.10	9.55	79	175.05	0.11
20	65.05	8.95	40	93.05	44.52	60	121.10	14.50	80	176.05	0.46

Peak#:19   R.Time:34.547(Scan#:3835)
MassPeaks:110
RawMode:Averaged 34.542-34.558(3834-3836)
BG Mode:Calc. from Peak Group 1 - Event 1 Scan

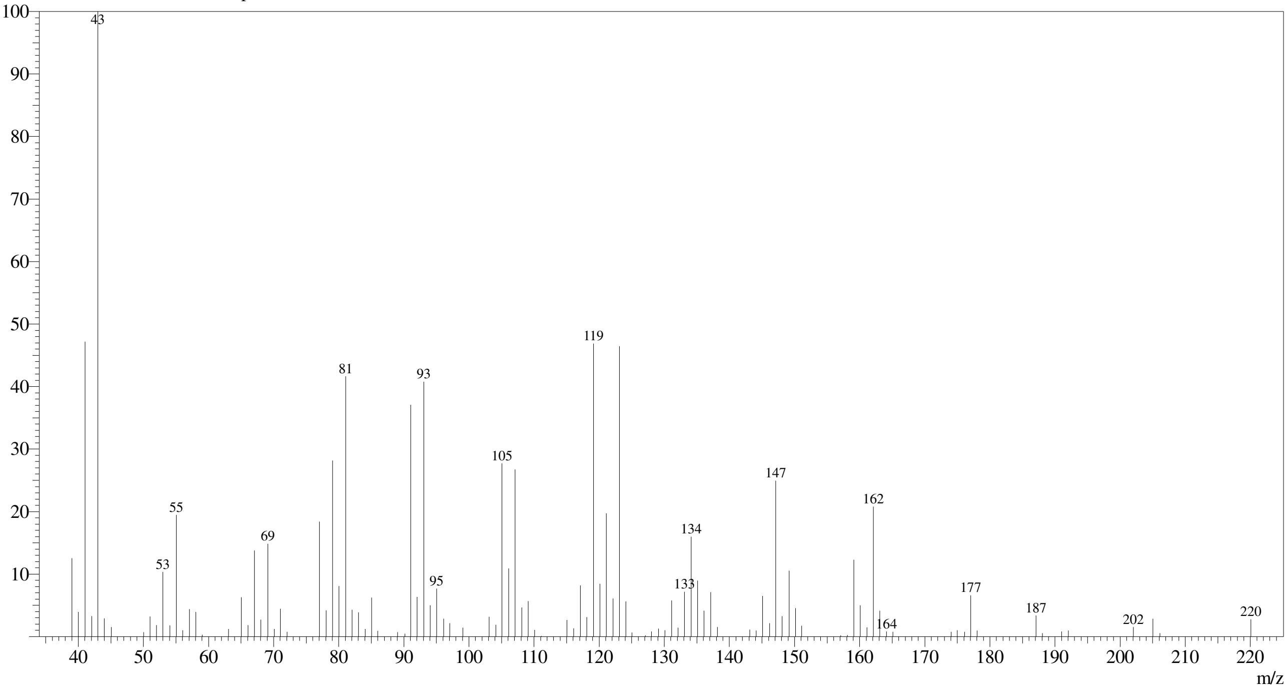

Mass Table
Peak#:19   R.Time:34.550(Scan#:3835)
MassPeaks:110
Group 1 - Event 1 Scan

#	m/z	Rel. Int.	#	m/z	Rel. Int.	#	m/z	Rel. Int.	#	m/z	Rel. Int.
1	39.00	12.55	29	79.05	28.16	57	116.10	1.33	85	149.15	10.57
2	40.00	3.97	30	80.05	8.11	58	117.10	8.19	86	150.15	4.56
3	41.05	47.16	31	81.05	41.65	59	118.10	3.13	87	151.10	1.76
4	42.05	3.30	32	82.05	4.31	60	119.10	46.86	88	157.10	0.23
5	43.00	100.00	33	83.05	3.89	61	120.10	8.47	89	158.10	0.23
6	44.00	2.91	34	84.05	1.25	62	121.10	19.72	90	159.10	12.29
7	45.05	1.54	35	85.05	6.24	63	122.10	6.11	91	160.10	5.03
8	50.05	0.73	36	86.00	0.95	64	123.10	46.46	92	161.10	1.49
9	51.00	3.21	37	89.05	0.74	65	124.10	5.64	93	162.10	20.79
10	52.00	1.85	38	90.15	0.47	66	125.05	0.65	94	163.10	4.14
11	53.00	10.34	39	91.05	37.08	67	127.10	0.23	95	164.10	0.84
12	54.05	1.79	40	92.05	6.37	68	128.05	0.82	96	165.10	0.78
13	55.05	19.45	41	93.05	40.80	69	129.10	1.27	97	174.05	0.79
14	56.05	1.01	42	94.05	5.02	70	130.10	1.02	98	175.00	1.01
15	57.05	4.41	43	95.05	7.69	71	131.10	5.81	99	176.10	0.79
16	58.05	3.93	44	96.10	2.89	72	132.10	1.43	100	177.05	6.63
17	59.05	0.31	45	97.05	2.14	73	133.10	7.19	101	178.05	0.95
18	63.05	1.23	46	99.05	1.45	74	134.10	16.00	102	187.10	3.37
19	65.05	6.29	47	103.10	3.16	75	135.10	8.98	103	188.05	0.58
20	66.05	1.85	48	104.15	1.90	76	136.10	4.16	104	189.10	0.01
21	67.05	13.78	49	105.05	27.72	77	137.10	7.12	105	191.00	0.81
22	68.05	2.72	50	106.10	10.92	78	138.15	1.55	106	192.05	0.97
23	69.10	14.85	51	107.10	26.73	79	143.10	1.11	107	202.05	1.55
24	70.10	1.22	52	108.10	4.65	80	144.10	0.98	108	205.05	2.87
25	71.05	4.48	53	109.10	5.70	81	145.10	6.50	109	206.10	0.56
26	72.05	0.76	54	110.10	1.08	82	146.15	2.15	110	220.10	2.78
27	77.05	18.40	55	111.10	0.09	83	147.10	24.95			
28	78.05	4.18	56	115.05	2.64	84	148.10	3.29			

Peak#:20 R.Time:36.019(Scan#:4011)
MassPeaks:131
RawMode:Averaged 36.008-36.025(4010-4012)
BG Mode:Calc. from Peak Group 1 - Event 1 Scan

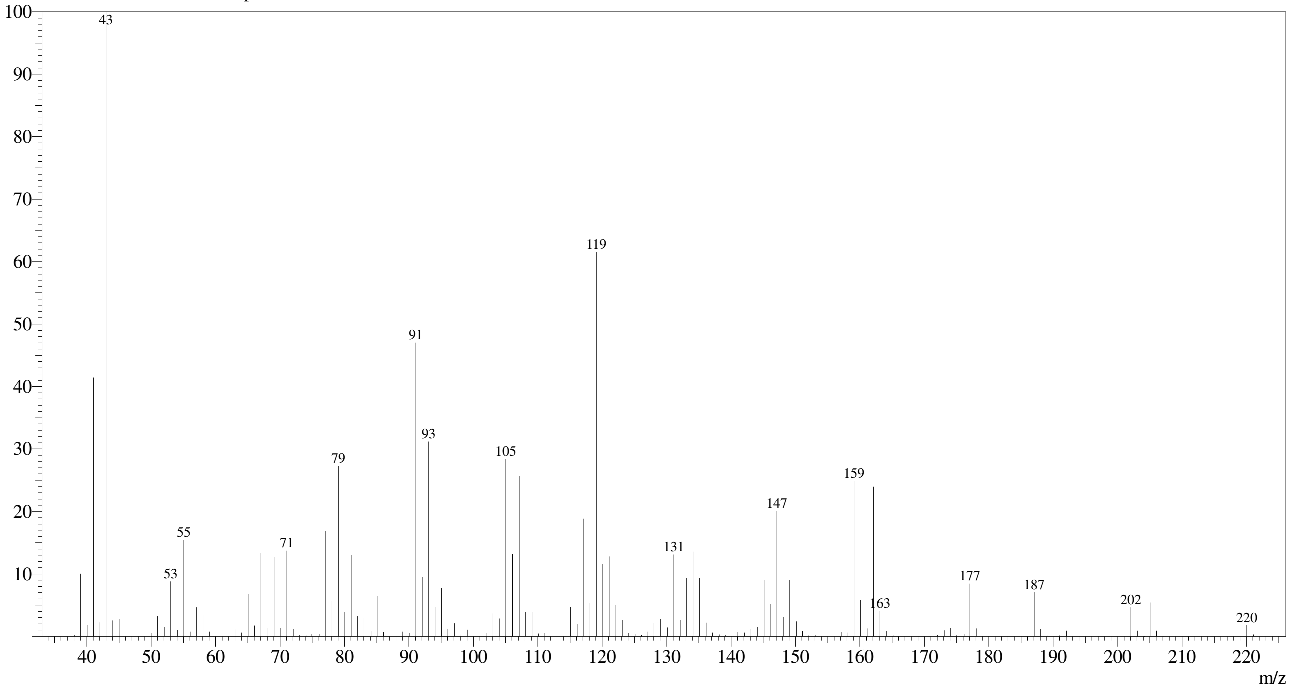

Mass Table
Peak#:20 R.Time:36.017(Scan#:4011)
MassPeaks:131
Group 1 - Event 1 Scan

#	m/z	Rel. Int.	#	m/z	Rel. Int.	#	m/z	Rel. Int.	#	m/z	Rel. Int.
1	38.05	0.26	34	78.05	5.69	67	118.10	5.33	100	152.10	0.27
2	39.00	10.05	35	79.05	27.23	68	119.10	61.55	101	153.10	0.13
3	40.05	1.86	36	80.05	3.88	69	120.10	11.59	102	157.10	0.65
4	41.05	41.43	37	81.05	13.02	70	121.10	12.82	103	158.15	0.59
5	42.05	2.27	38	82.05	3.24	71	122.10	5.09	104	159.10	24.91
6	43.00	100.00	39	83.05	3.02	72	123.10	2.67	105	160.10	5.86
7	44.05	2.56	40	84.10	0.80	73	124.10	0.52	106	161.10	1.26
8	45.00	2.74	41	85.05	6.44	74	125.10	0.33	107	162.10	23.97
9	50.00	0.56	42	86.05	0.69	75	126.10	0.26	108	163.10	4.09
10	51.00	3.21	43	89.05	0.74	76	127.10	0.77	109	164.10	0.88
11	52.05	1.47	44	90.15	0.52	77	128.05	2.18	110	165.15	0.20
12	53.00	8.80	45	91.05	47.03	78	129.05	2.84	111	172.05	0.18
13	54.05	1.00	46	92.05	9.46	79	130.10	1.44	112	173.10	0.97
14	55.05	15.40	47	93.05	31.18	80	131.10	13.09	113	174.05	1.40
15	56.05	0.79	48	94.05	4.72	81	132.10	2.61	114	175.05	0.28
16	57.05	4.68	49	95.05	7.73	82	133.10	9.35	115	176.15	0.39
17	58.05	3.56	50	96.05	1.23	83	134.10	13.59	116	177.05	8.44
18	59.05	0.78	51	97.10	2.08	84	135.10	9.32	117	178.05	1.27
19	63.00	1.13	52	98.10	0.32	85	136.15	2.19	118	179.00	0.08
20	64.00	0.61	53	99.10	1.10	86	137.15	0.63	119	187.05	7.07
21	65.05	6.80	54	102.10	0.49	87	138.15	0.32	120	188.05	1.20
22	66.05	1.73	55	103.05	3.68	88	139.15	0.23	121	189.05	0.24
23	67.05	13.39	56	104.10	2.85	89	141.05	0.68	122	191.05	0.25
24	68.10	1.38	57	105.05	28.36	90	142.10	0.60	123	192.05	0.91
25	69.05	12.68	58	106.10	13.23	91	143.10	1.16	124	202.05	4.64
26	70.10	1.32	59	107.10	25.68	92	144.10	1.50	125	203.10	0.92
27	71.05	13.71	60	108.10	3.93	93	145.10	9.09	126	204.15	0.02
28	72.05	1.19	61	109.10	3.92	94	146.15	5.16	127	205.05	5.45
29	73.00	0.28	62	110.10	0.44	95	147.10	20.10	128	206.05	0.94
30	74.05	0.16	63	111.10	0.53	96	148.10	3.05	129	207.05	0.06
31	74.95	0.37	64	115.05	4.71	97	149.10	9.05	130	220.05	1.77
32	76.05	0.43	65	116.10	1.93	98	150.15	2.43	131	221.10	0.30
33	77.00	16.88	66	117.05	18.86	99	151.10	0.87			

Library

<< Target >>
Line#:1   R.Time:6.900(Scan#:517) MassPeaks:77
RawMode:Averaged 6.892-6.908(516-518) BasePeak:93.10(3575953)
BG Mode:Calc. from Peak Group 1 - Event 1 Scan

Target Spectrum

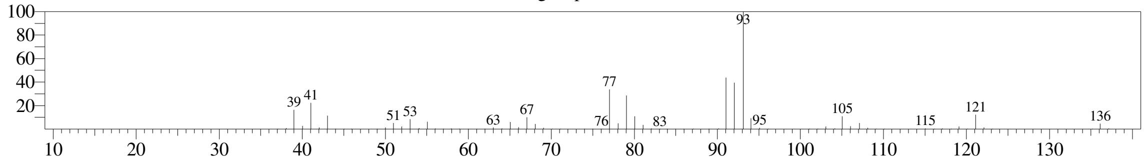

Hit#:1 Entry:6850 Library:NIST14s.lib
SI:97 Formula:C10H16 CAS:80-56-8 MolWeight:136 RetIndex:948
CompName:.alpha.-Pinene $$ Bicyclo[3.1.1]hept-2-ene, 2,6,6-trimethyl- $$ 2-Pinene $$ 2,6,6-Trimethylbicyclo[3.1.1]hept-2-ene $$ Pinene, .alpha. $$ 2,6,6

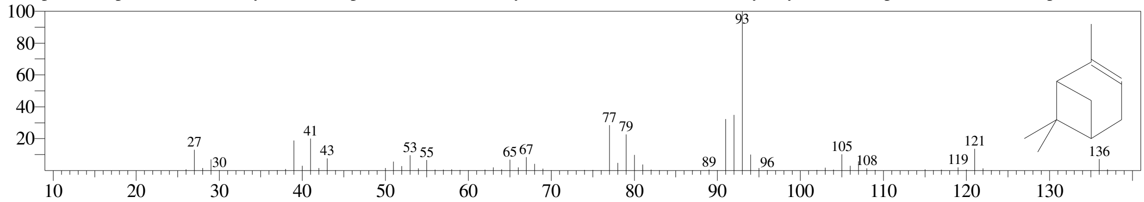

Hit#:2 Entry:6844 Library:NIST14s.lib
SI:96 Formula:C10H16 CAS:3779-61-1 MolWeight:136 RetIndex:976
CompName:trans-.beta.-Ocimene $$ 1,3,6-Octatriene, 3,7-dimethyl-, (E)- $$ .beta.-trans-Ocimene $$ trans-3,7-Dimethyl-1,3,6-Octatriene $$ Ocimene, trans

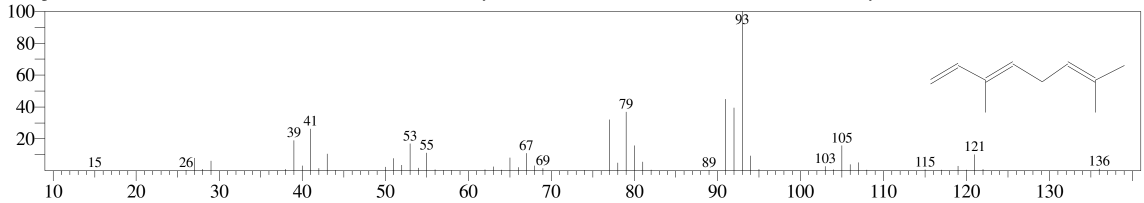

Hit#:3 Entry:10017 Library:NIST14.lib
SI:95 Formula:C10H16 CAS:80-56-8 MolWeight:136 RetIndex:948
CompName:.alpha.-Pinene $$ Bicyclo[3.1.1]hept-2-ene, 2,6,6-trimethyl- $$ 2-Pinene $$ 2,6,6-Trimethylbicyclo[3.1.1]hept-2-ene $$ Pinene, .alpha. $$ 2,6,6

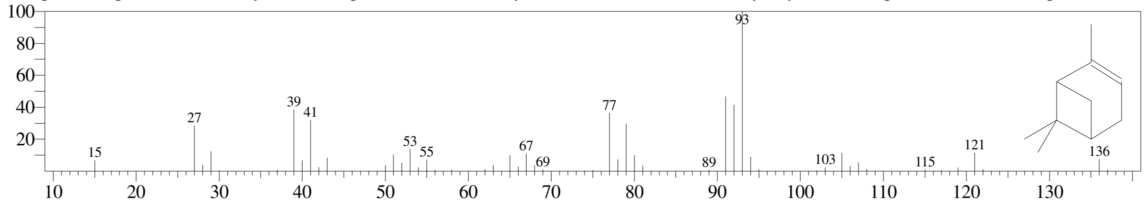

Hit#:4 Entry:6849 Library:NIST14s.lib
SI:95 Formula:C10H16 CAS:80-56-8 MolWeight:136 RetIndex:948
CompName:.alpha.-Pinene $$ Bicyclo[3.1.1]hept-2-ene, 2,6,6-trimethyl- $$ 2-Pinene $$ 2,6,6-Trimethylbicyclo[3.1.1]hept-2-ene $$ Pinene, .alpha. $$ 2,6,6

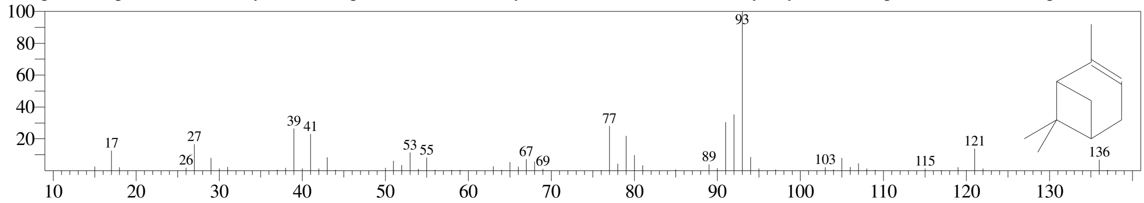

Hit#:5 Entry:6848 Library:NIST14s.lib
SI:94 Formula:C10H16 CAS:488-97-1 MolWeight:136 RetIndex:729
CompName:Tricyclo[2.2.1.0(2,6)]heptane, 1,3,3-trimethyl- $$ Cyclofenchene $$ Tricyclo[2.2.1.02,6]heptane, 1,3,3-trimethyl- $$

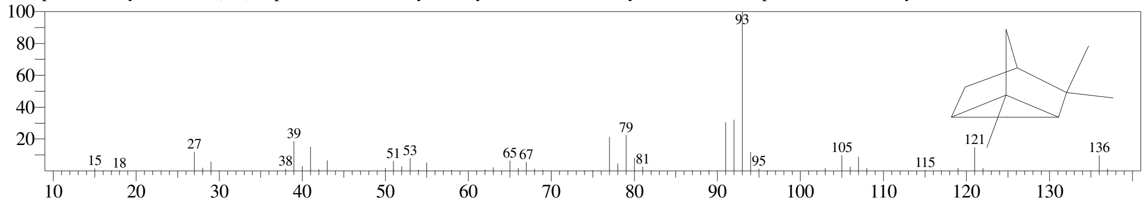

<< Target >>
Line#:2   R.Time:7.575(Scan#:598) MassPeaks:70
RawMode:Averaged 7.567-7.583(597-599) BasePeak:91.05(513477)
BG Mode:Calc. from Peak Group 1 - Event 1 Scan

Target Spectrum

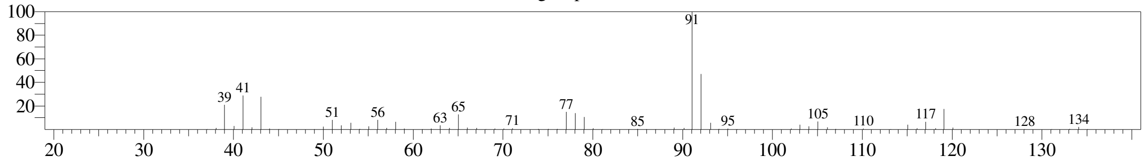

Hit#:1 Entry:6334 Library:NIST14s.lib
SI:92 Formula:C10H14 CAS:36262-09-6 MolWeight:134 RetIndex:879
CompName:Bicyclo[3.1.0]hex-2-ene, 4-methylene-1-(1-methylethyl)- $$ 1-Isopropyl-4-methylenebicyclo[3.1.0]hex-2-ene $$ 2,4(10)-Thujadiene $$ 4-Meth

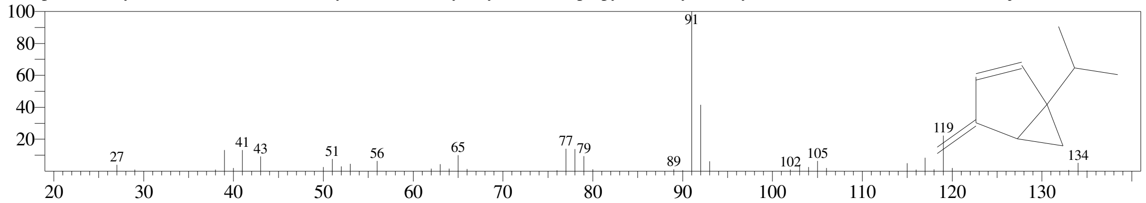

Hit#:2 Entry:6333 Library:NIST14s.lib
SI:88 Formula:C10H14 CAS:36262-09-6 MolWeight:134 RetIndex:879
CompName:Bicyclo[3.1.0]hex-2-ene, 4-methylene-1-(1-methylethyl)- $$ 1-Isopropyl-4-methylenebicyclo[3.1.0]hex-2-ene $$ 2,4(10)-Thujadiene $$ 4-Meth

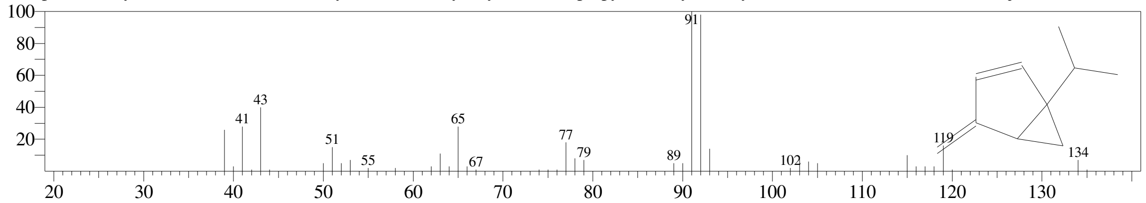

Hit#:3 Entry:16873 Library:NIST14.lib
SI:88 Formula:C10H16O CAS:97631-68-0 MolWeight:152 RetIndex:1023
CompName:Bicyclo[3.1.0]hex-3-en-2-ol, 2-methyl-5-(1-methylethyl)-, (1.alpha.,2.alpha.,5.alpha.)- $$ 5-Isopropyl-2-methylbicyclo[3.1.0]hex-3-en-2-ol # $$

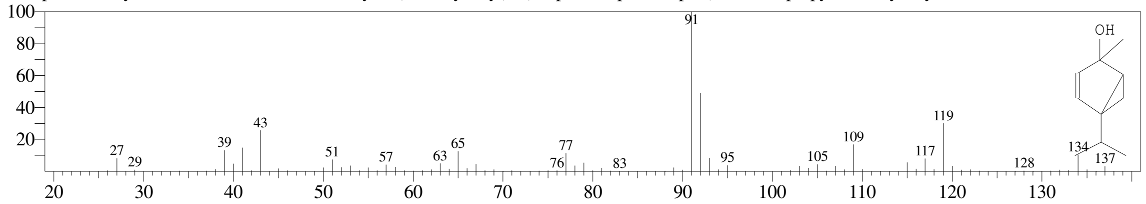

Hit#:4 Entry:9273 Library:NIST14.lib
SI:87 Formula:C10H14 CAS:36262-09-6 MolWeight:134 RetIndex:879
CompName:Bicyclo[3.1.0]hex-2-ene, 4-methylene-1-(1-methylethyl)- $$ 1-Isopropyl-4-methylenebicyclo[3.1.0]hex-2-ene $$ 2,4(10)-Thujadiene $$ 4-Meth

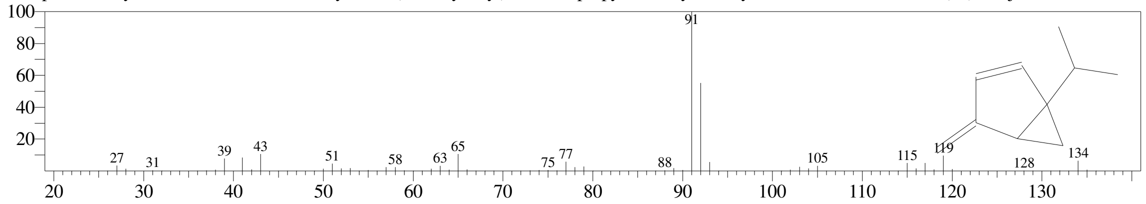

Hit#:5 Entry:9274 Library:NIST14.lib
SI:86 Formula:C10H14 CAS:0-00-0 MolWeight:134 RetIndex:1023
CompName:Bicyclo[4.2.0]oct-1-ene, 7-exo-ethenyl- $$ 7-Vinylbicyclo[4.2.0]oct-1-ene # $$

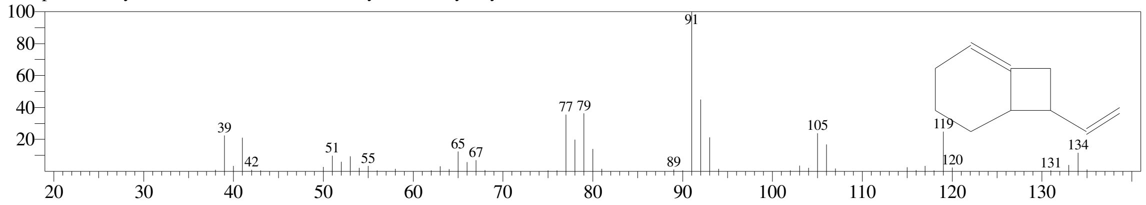

<< Target >>
Line#:3 R.Time:8.467(Scan#:705) MassPeaks:76
RawMode:Averaged 8.458-8.475(704-706) BasePeak:93.10(3875095)
BG Mode:Calc. from Peak Group 1 - Event 1 Scan

Target Spectrum

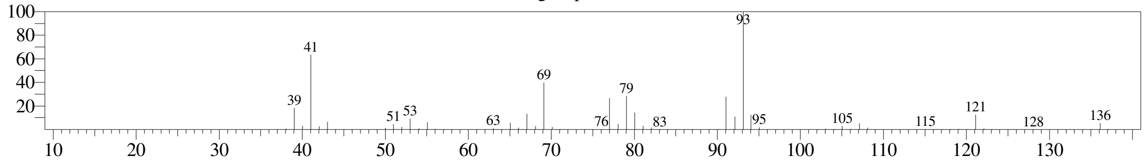

Hit#:1 Entry:9984 Library:NIST14.lib
SI:96 Formula:C10H16 CAS:127-91-3 MolWeight:136 RetIndex:943
CompName:.beta.-Pinene $$ Bicyclo[3.1.1]heptane, 6,6-dimethyl-2-methylene- $$ 2(10)-Pinene $$ Nopinen $$ Nopinene $$ Pseudopinen $$ Pseudopinene

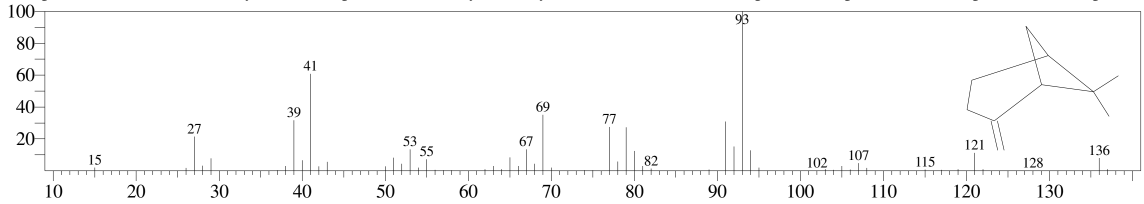

Hit#:2 Entry:6809 Library:NIST14s.lib
SI:96 Formula:C10H16 CAS:127-91-3 MolWeight:136 RetIndex:943
CompName:.beta.-Pinene $$ Bicyclo[3.1.1]heptane, 6,6-dimethyl-2-methylene- $$ 2(10)-Pinene $$ Nopinen $$ Nopinene $$ Pseudopinen $$ Pseudopinene

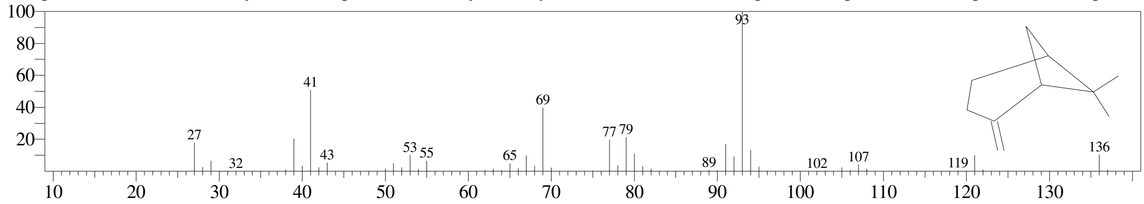

Hit#:3 Entry:6816 Library:NIST14s.lib
SI:95 Formula:C10H16 CAS:18172-67-3 MolWeight:136 RetIndex:943
CompName:Bicyclo[3.1.1]heptane, 6,6-dimethyl-2-methylene-, (1S)- $$ 2(10)-Pinene, (1S,5S)-(-)- $$ (-)-.beta.-Pinene $$ (-)-2(10)-Pinene $$ L-.beta.-Piner

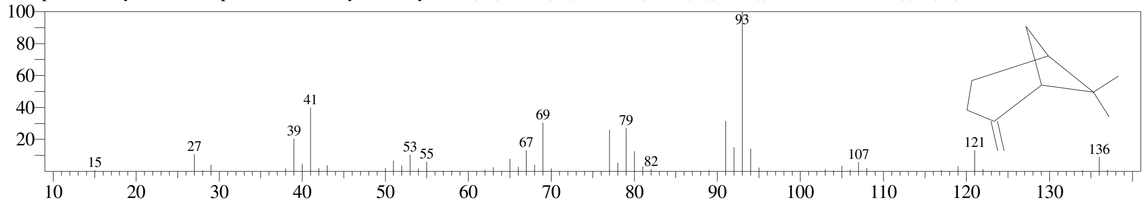

Hit#:4 Entry:6808 Library:NIST14s.lib
SI:95 Formula:C10H16 CAS:127-91-3 MolWeight:136 RetIndex:943
CompName:.beta.-Pinene $$ Bicyclo[3.1.1]heptane, 6,6-dimethyl-2-methylene- $$ 2(10)-Pinene $$ Nopinen $$ Nopinene $$ Pseudopinen $$ Pseudopinene

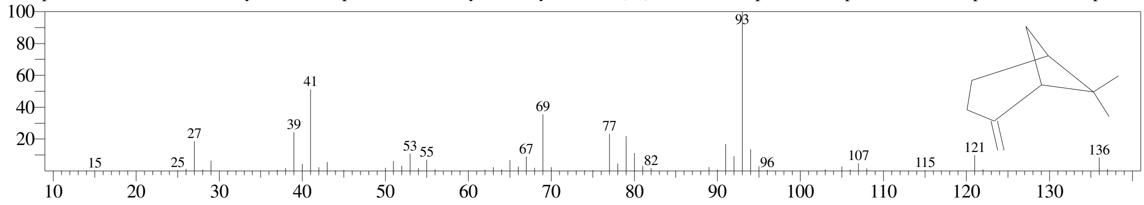

Hit#:5 Entry:6807 Library:NIST14s.lib
SI:94 Formula:C10H16 CAS:127-91-3 MolWeight:136 RetIndex:943
CompName:.beta.-Pinene $$ Bicyclo[3.1.1]heptane, 6,6-dimethyl-2-methylene- $$ 2(10)-Pinene $$ Nopinen $$ Nopinene $$ Pseudopinen $$ Pseudopinene

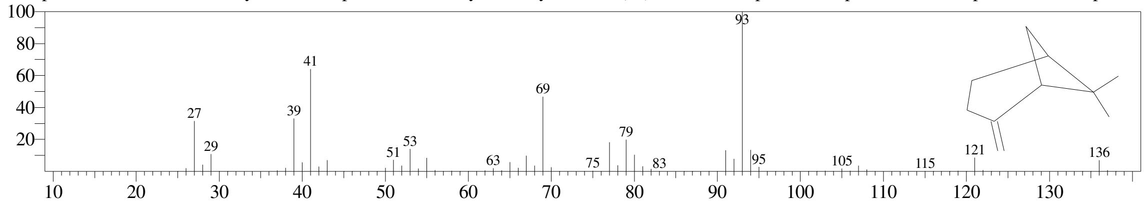

<< Target >>
Line#:4   R.Time:10.317(Scan#:927) MassPeaks:65
RawMode:Averaged 10.308-10.325(926-928) BasePeak:119.10(768601)
BG Mode:Calc. from Peak Group 1 - Event 1 Scan

Target Spectrum

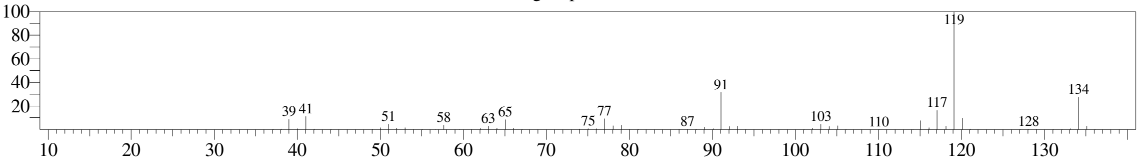

Hit#:1 Entry:6359 Library:NIST14s.lib
SI:96 Formula:C10H14 CAS:99-87-6 MolWeight:134 RetIndex:1042
CompName:p-Cymene $$ Benzene, 1-methyl-4-(1-methylethyl)- $$ p-Cimene $$ p-Cymol $$ p-Isopropyltoluene $$ p-Methylisopropylbenzene $$ Campho

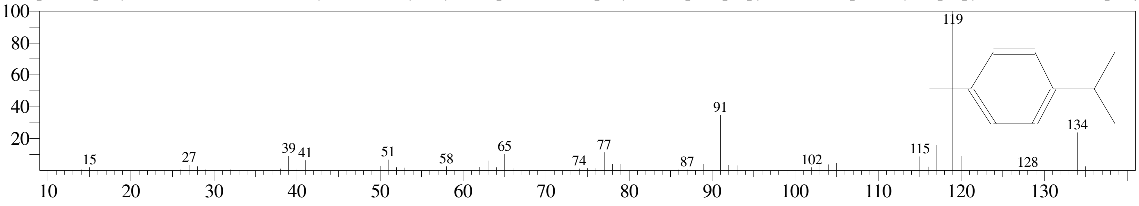

Hit#:2 Entry:6390 Library:NIST14s.lib
SI:95 Formula:C10H14 CAS:527-84-4 MolWeight:134 RetIndex:1042
CompName:o-Cymene $$ Benzene, 1-methyl-2-(1-methylethyl)- $$ o-Cymol $$ o-Isopropyltoluene $$ 1-Isopropyl-2-methylbenzene $$ 1-Methyl-2-isoprop

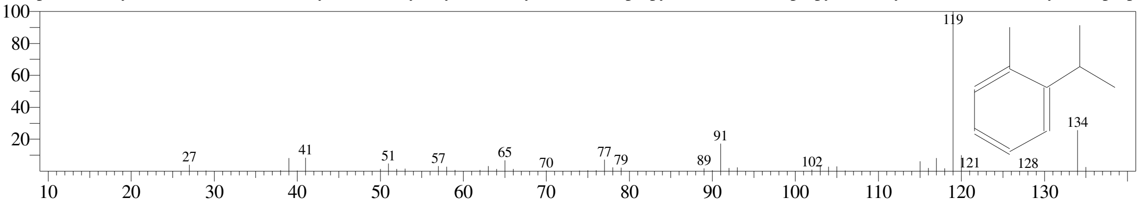

Hit#:3 Entry:6363 Library:NIST14s.lib
SI:95 Formula:C10H14 CAS:535-77-3 MolWeight:134 RetIndex:1042
CompName:Benzene, 1-methyl-3-(1-methylethyl)- $$ m-Cymene $$ .beta.-Cymene $$ m-Cymol $$ m-Isopropyltoluene $$ m-Methylisopropylbenzene $$ 1-

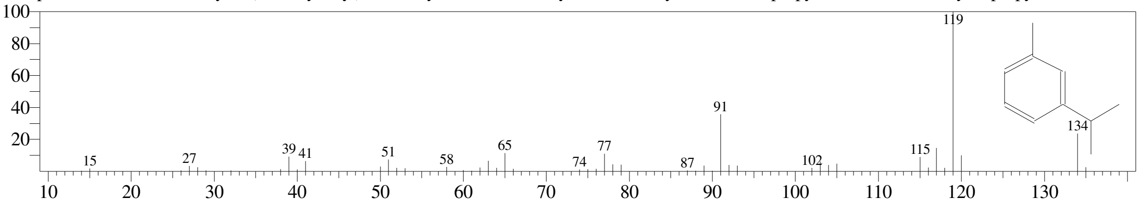

Hit#:4 Entry:6371 Library:NIST14s.lib
SI:95 Formula:C10H14 CAS:99-87-6 MolWeight:134 RetIndex:1042
CompName:p-Cymene $$ Benzene, 1-methyl-4-(1-methylethyl)- $$ p-Cimene $$ p-Cymol $$ p-Isopropyltoluene $$ p-Methylisopropylbenzene $$ Campho

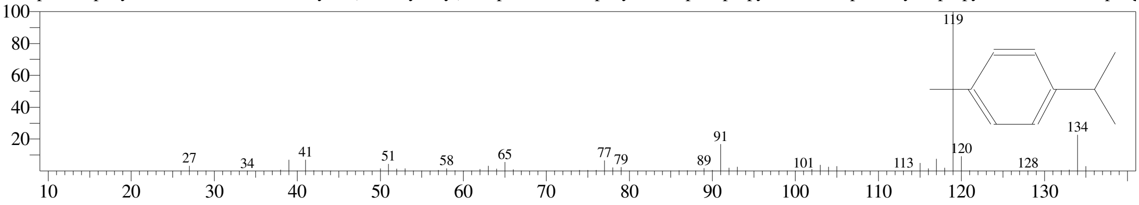

Hit#:5 Entry:9307 Library:NIST14.lib
SI:95 Formula:C10H14 CAS:3479-89-8 MolWeight:134 RetIndex:1010
CompName:1,3,5-Cycloheptatriene, 3,7,7-trimethyl- $$ 3,7,7-Trimethyl-1,3,5-cycloheptatriene # $$ 3,7,7-Trimethylcyclohepta-1,3,5-triene $$

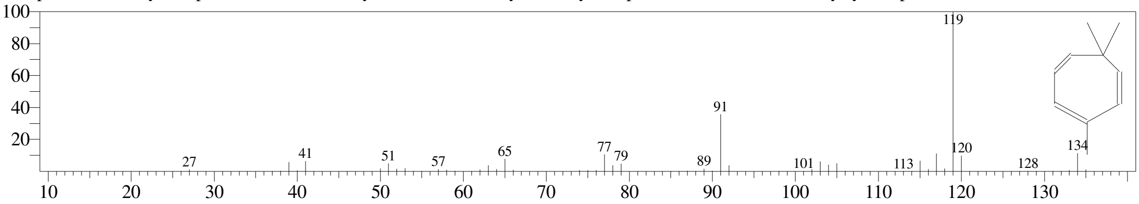

<< Target >>
Line#:5 R.Time:10.617(Scan#:963) MassPeaks:69
RawMode:Averaged 10.608-10.625(962-964) BasePeak:43.00(556209)
BG Mode:Calc. from Peak Group 1 - Event 1 Scan

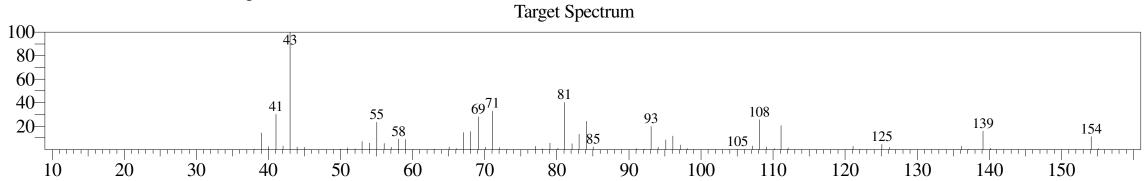

Hit#:1 Entry:10305 Library:NIST14s.lib
SI:88 Formula:C10H18O CAS:470-82-6 MolWeight:154 RetIndex:1059
CompName:Eucalyptol $$ Cineole $$ 2-Oxabicyclo[2.2.2]octane, 1,3,3-trimethyl- $$ p-Menthane, 1,8-epoxy- $$ p-Cineole $$ Cajeputol $$ Cucalyptol $$ I

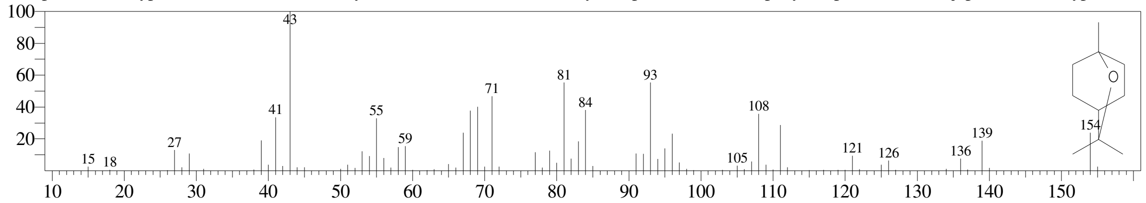

Hit#:2 Entry:17977 Library:NIST14.lib
SI:88 Formula:C10H18O CAS:470-82-6 MolWeight:154 RetIndex:1059
CompName:Eucalyptol $$ Cineole $$ 2-Oxabicyclo[2.2.2]octane, 1,3,3-trimethyl- $$ p-Menthane, 1,8-epoxy- $$ p-Cineole $$ Cajeputol $$ Cucalyptol $$ I

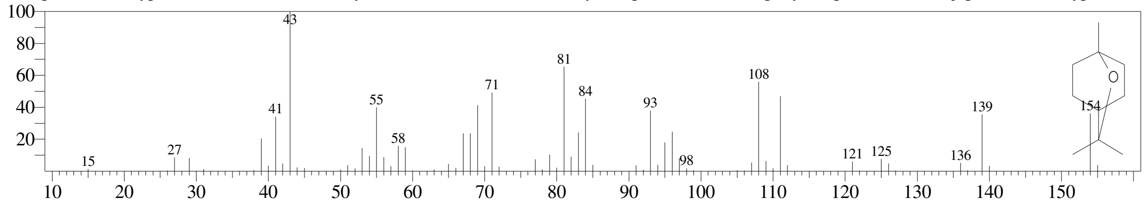

Hit#:3 Entry:10306 Library:NIST14s.lib
SI:88 Formula:C10H18O CAS:470-82-6 MolWeight:154 RetIndex:1059
CompName:Eucalyptol $$ Cineole $$ 2-Oxabicyclo[2.2.2]octane, 1,3,3-trimethyl- $$ p-Menthane, 1,8-epoxy- $$ p-Cineole $$ Cajeputol $$ Cucalyptol $$ I

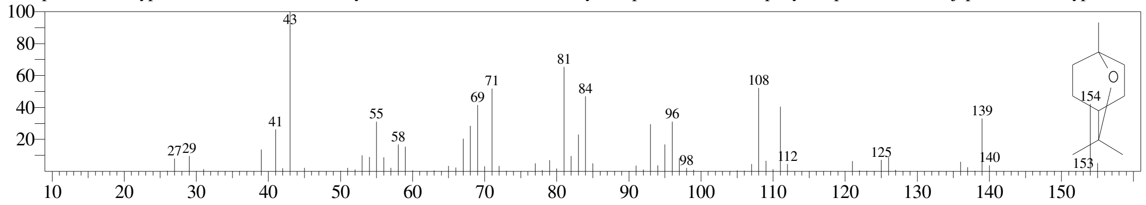

Hit#:4 Entry:10304 Library:NIST14s.lib
SI:87 Formula:C10H18O CAS:470-82-6 MolWeight:154 RetIndex:1059
CompName:Eucalyptol $$ Cineole $$ 2-Oxabicyclo[2.2.2]octane, 1,3,3-trimethyl- $$ p-Menthane, 1,8-epoxy- $$ p-Cineole $$ Cajeputol $$ Cucalyptol $$ I

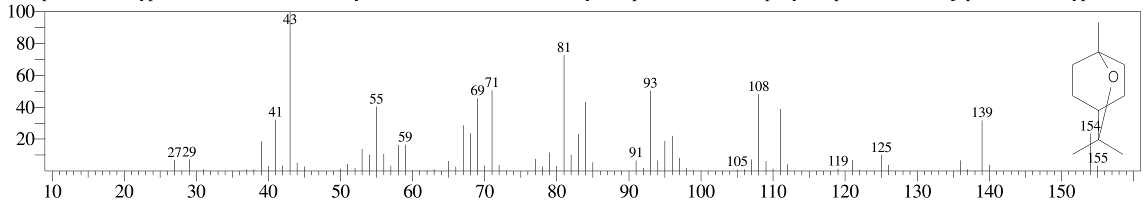

Hit#:5 Entry:10307 Library:NIST14s.lib
SI:86 Formula:C10H18O CAS:470-82-6 MolWeight:154 RetIndex:1059
CompName:Eucalyptol $$ Cineole $$ 2-Oxabicyclo[2.2.2]octane, 1,3,3-trimethyl- $$ p-Menthane, 1,8-epoxy- $$ p-Cineole $$ Cajeputol $$ Cucalyptol $$ I

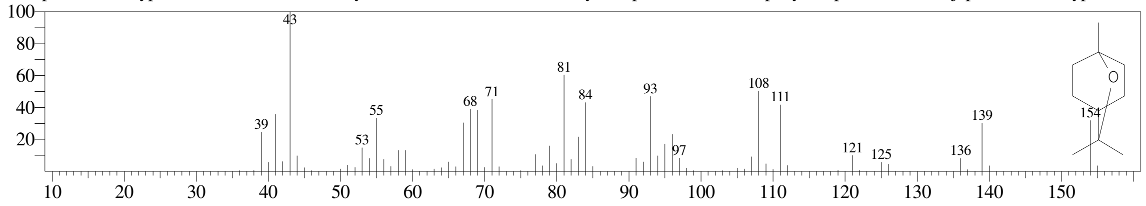

<< Target >>
Line#:6   R.Time:14.742(Scan#:1458) MassPeaks:62
RawMode:Averaged 14.733-14.750(1457-1459) BasePeak:108.10(412783)
BG Mode:Calc. from Peak Group 1 - Event 1 Scan

Target Spectrum

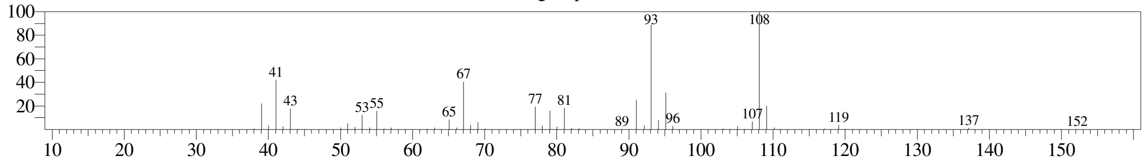

Hit#:1 Entry:9902 Library:NIST14s.lib
SI:92 Formula:C10H16O CAS:4501-58-0 MolWeight:152 RetIndex:1155
CompName:.alpha.-Campholenal $$ (R)-.alpha.-Campholene aldehyde $$ (R)-2-(2,2,3-Trimethylcyclopent-3-en-1-yl)acetaldehyde $$ 3-Cyclopentene-1-ace

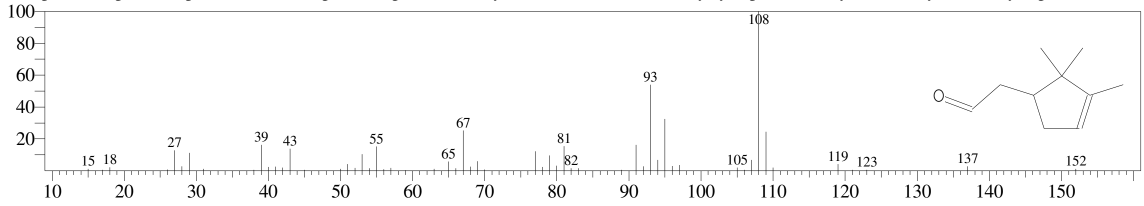

Hit#:2 Entry:9901 Library:NIST14s.lib
SI:91 Formula:C10H16O CAS:4501-58-0 MolWeight:152 RetIndex:1155
CompName:.alpha.-Campholenal $$ (R)-.alpha.-Campholene aldehyde $$ (R)-2-(2,2,3-Trimethylcyclopent-3-en-1-yl)acetaldehyde $$ 3-Cyclopentene-1-ace

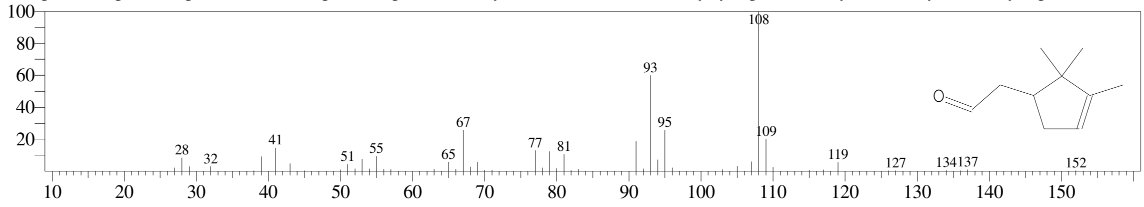

Hit#:3 Entry:9903 Library:NIST14s.lib
SI:89 Formula:C10H16O CAS:4501-58-0 MolWeight:152 RetIndex:1155
CompName:.alpha.-Campholenal $$ (R)-.alpha.-Campholene aldehyde $$ (R)-2-(2,2,3-Trimethylcyclopent-3-en-1-yl)acetaldehyde $$ 3-Cyclopentene-1-ace

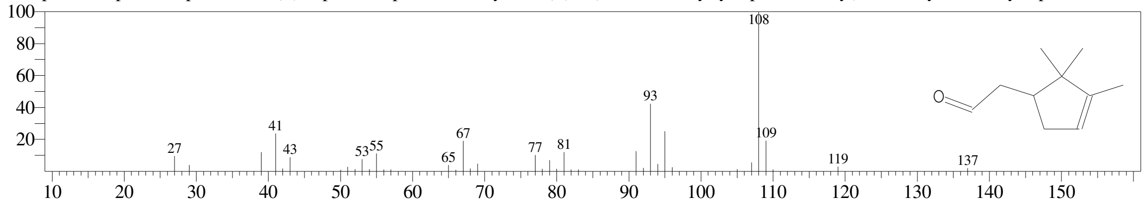

Hit#:4 Entry:16922 Library:NIST14.lib
SI:89 Formula:C10H16O CAS:0-00-0 MolWeight:152 RetIndex:1120
CompName:1,7,7-Trimethylbicyclo[2.2.1]hept-5-en-2-ol

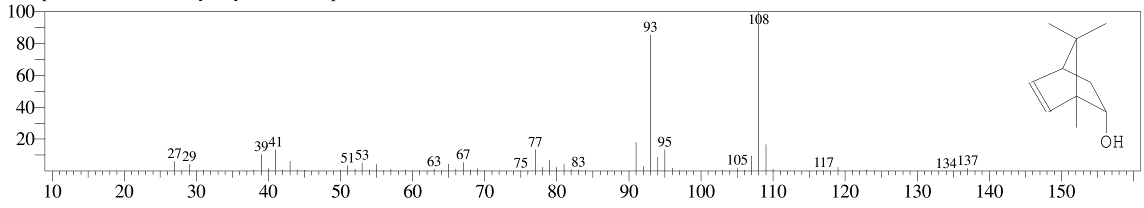

Hit#:5 Entry:16885 Library:NIST14.lib
SI:88 Formula:C10H16O CAS:3570-04-5 MolWeight:152 RetIndex:1131
CompName:Camphenol, 6- $$ 5,5-Dimethyl-6-methylenebicyclo[2.2.1]heptan-2-ol # $$ Camphen-6-ol $$ Camphenol $$ 6-Hydroxycamphene $$

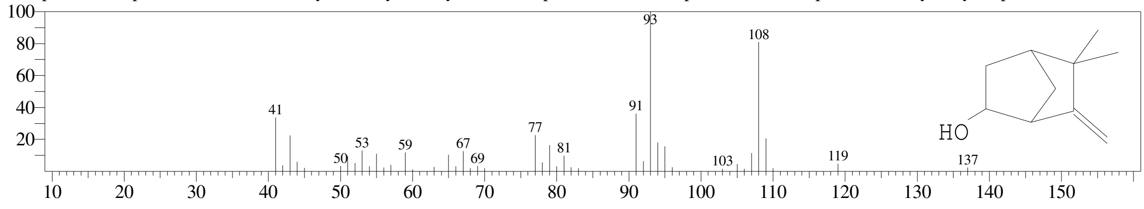

<< Target >>
Line#:7 R.Time:15.242(Scan#:1518) MassPeaks:60
RawMode:Averaged 15.233-15.250(1517-1519) BasePeak:83.05(357823)
BG Mode:Calc. from Peak Group 1 - Event 1 Scan

Target Spectrum

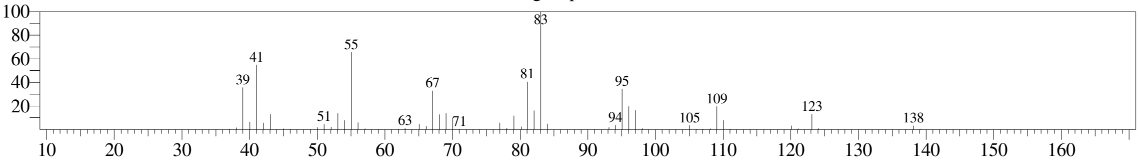

Hit#:1 Entry:10647 Library:NIST14.lib
SI:94 Formula:C9H14O CAS:38651-65-9 MolWeight:138 RetIndex:1047
CompName:Bicyclo[3.1.1]heptan-2-one, 6,6-dimethyl-, (1R)- $$ (1R)-(+)-Nopinone $$ 6,6-Dimethylbicyclo[3.1.1]heptan-2-one-, (1R)- $$ (+)-Nopinone $$

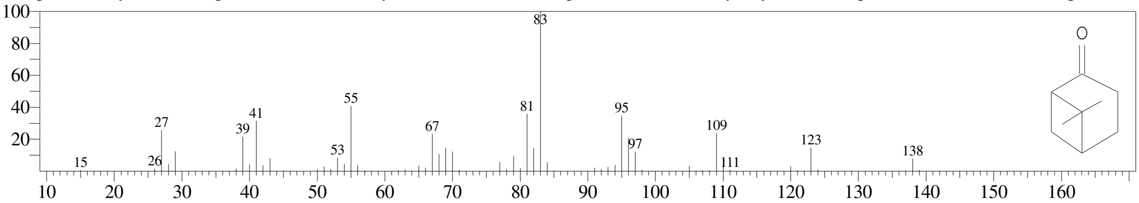

Hit#:2 Entry:10646 Library:NIST14.lib
SI:91 Formula:C9H14O CAS:24903-95-5 MolWeight:138 RetIndex:1047
CompName:Bicyclo[3.1.1]heptan-2-one, 6,6-dimethyl- $$ .beta.-Pinone $$ Nopinon $$ Nopinone $$ 2-Norpinanone, 6,6-dimethyl- $$ 6,6-Dimethylbicyclo

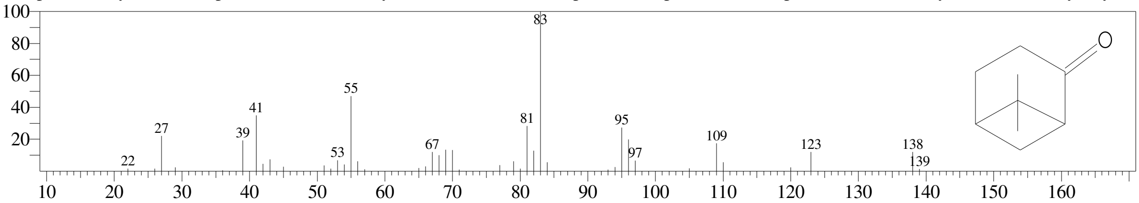

Hit#:3 Entry:7183 Library:NIST14s.lib
SI:90 Formula:C9H14O CAS:24903-95-5 MolWeight:138 RetIndex:1047
CompName:Bicyclo[3.1.1]heptan-2-one, 6,6-dimethyl- $$ .beta.-Pinone $$ Nopinon $$ Nopinone $$ 2-Norpinanone, 6,6-dimethyl- $$ 6,6-Dimethylbicyclo

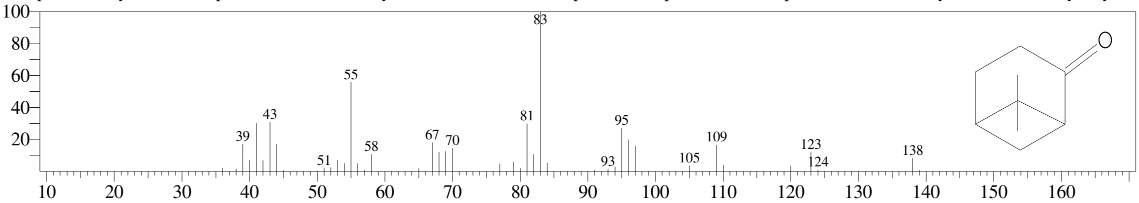

Hit#:4 Entry:7207 Library:NIST14s.lib
SI:87 Formula:C10H18 CAS:85006-04-8 MolWeight:138 RetIndex:926
CompName:1,6-Octadiene, 5,7-dimethyl-, (R)- $$ R(+)5,7-Dimethyl-1,6-octadiene $$ 5,7-Dimethyl-1,6-octadiene, (R)- $$

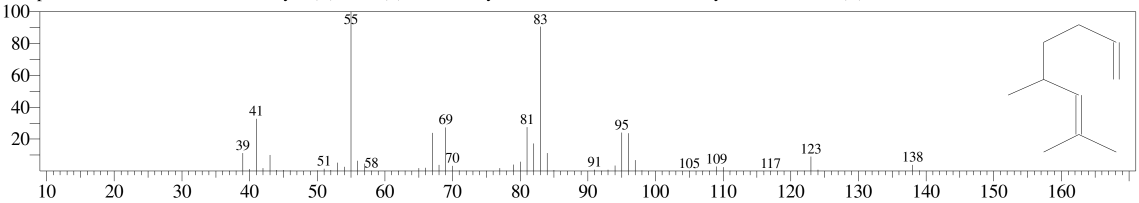

Hit#:5 Entry:111661 Library:NIST14.lib
SI:87 Formula:C18H30O2 CAS:0-00-0 MolWeight:278 RetIndex:1873
CompName:Cyclopentanecarboxylic acid, 3-methyl-4-methylene-, menthyl ester $$ 2-Isopropyl-5-methylcyclohexyl 3-methyl-4-methylenecyclopentanecarb

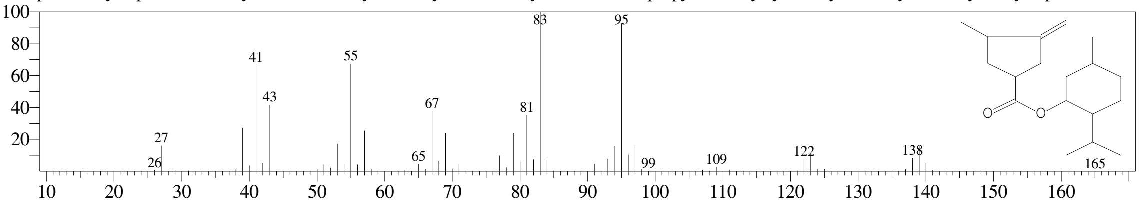

<< Target >>
Line#:8 R.Time:15.442(Scan#:1542) MassPeaks:94
RawMode:Averaged 15.433-15.450(1541-1543) BasePeak:41.05(1280736)
BG Mode:Calc. from Peak Group 1 - Event 1 Scan

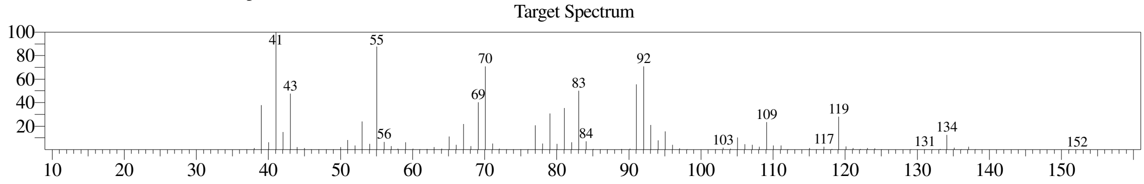

Hit#:1 Entry:9785 Library:NIST14s.lib
SI:95 Formula:C10H16O CAS:547-61-5 MolWeight:152 RetIndex:1131
CompName:Bicyclo[3.1.1]heptan-3-ol, 6,6-dimethyl-2-methylene-, [1S-(1.alpha.,3.alpha.,5.alpha.)]- $$ (1S,3R,5S)-6,6-Dimethyl-2-methylenebicyclo[3.1.1]

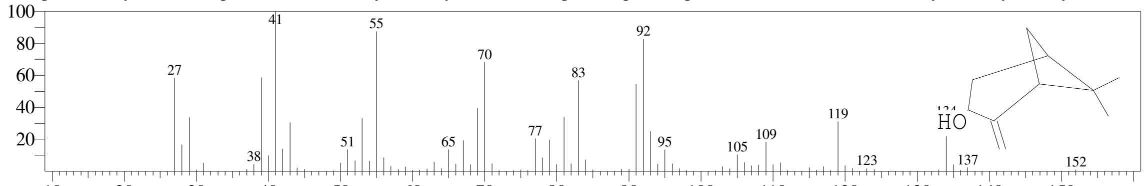

Hit#:2 Entry:16745 Library:NIST14.lib
SI:94 Formula:C10H16O CAS:6712-79-4 MolWeight:152 RetIndex:1131
CompName:Isopinocarveol $$ 6,6-Dimethyl-2-methylenebicyclo[3.1.1]heptan-3-ol # $$ cis-Pinocarveol $$ Bicyclo[3.1.1]heptan-3-ol, 6,6-dimethyl-2-methy

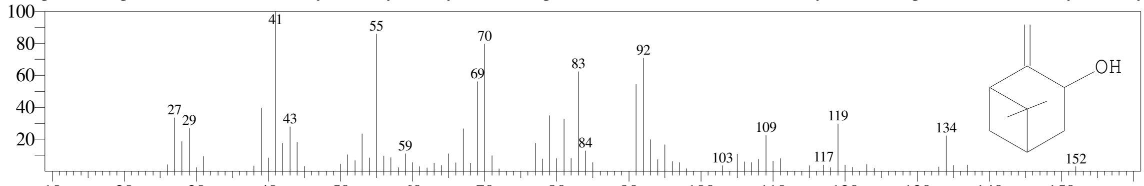

Hit#:3 Entry:16876 Library:NIST14.lib
SI:94 Formula:C10H16O CAS:547-61-5 MolWeight:152 RetIndex:1131
CompName:Bicyclo[3.1.1]heptan-3-ol, 6,6-dimethyl-2-methylene-, [1S-(1.alpha.,3.alpha.,5.alpha.)]- $$ (1S,3R,5S)-6,6-Dimethyl-2-methylenebicyclo[3.1.1]

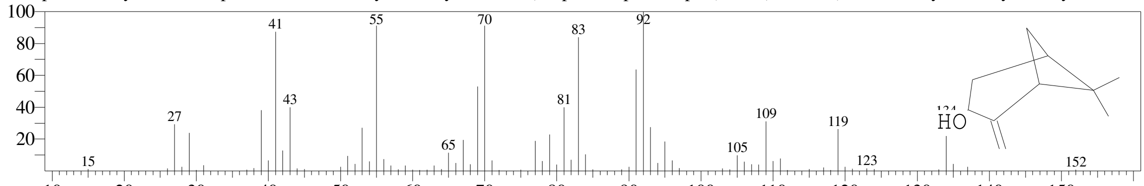

Hit#:4 Entry:16806 Library:NIST14.lib
SI:94 Formula:C10H16O CAS:5947-36-4 MolWeight:152 RetIndex:1131
CompName:Bicyclo[3.1.1]heptan-3-ol, 6,6-dimethyl-2-methylene- $$ Pinocarveol $$ 2(10)-Pinen-3-ol $$ 6,6-Dimethyl-2-methylenebicyclo[3.1.1]heptan-3-

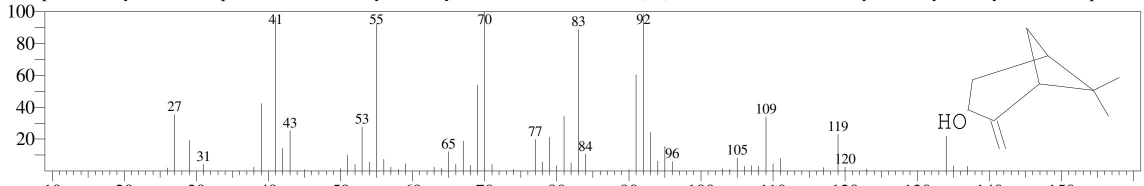

Hit#:5 Entry:9878 Library:NIST14s.lib
SI:92 Formula:C10H16O CAS:547-61-5 MolWeight:152 RetIndex:1131
CompName:Bicyclo[3.1.1]heptan-3-ol, 6,6-dimethyl-2-methylene-, [1S-(1.alpha.,3.alpha.,5.alpha.)]- $$ (1S,3R,5S)-6,6-Dimethyl-2-methylenebicyclo[3.1.1]

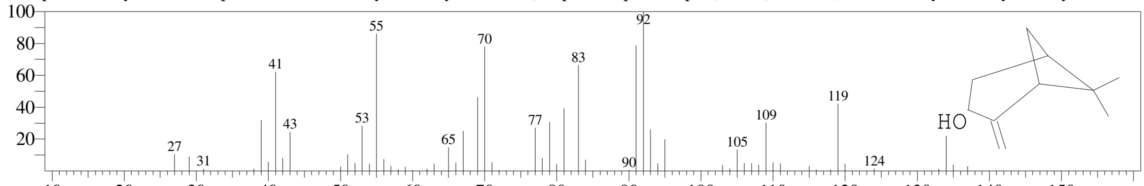

<< Target >>
Line#:9 R.Time:15.658(Scan#:1568) MassPeaks:88
RawMode:Averaged 15.650-15.667(1567-1569) BasePeak:41.05(432134)
BG Mode:Calc. from Peak Group 1 - Event 1 Scan

Target Spectrum

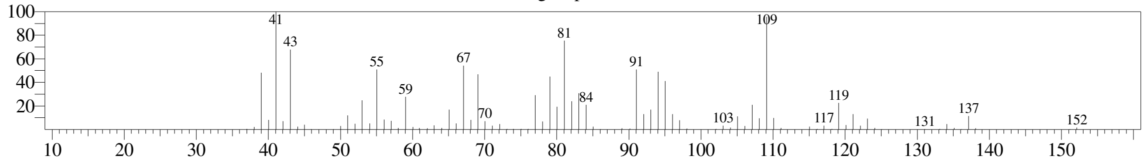

Hit#:1 Entry:9792 Library:NIST14s.lib
SI:93 Formula:C10H16O CAS:473-67-6 MolWeight:152 RetIndex:1136
CompName:Verbenol $$ Bicyclo[3.1.1]hept-3-en-2-ol, 4,6,6-trimethyl- $$ Berbenol $$ 2-Pinen-4-ol $$ 4,6,6-Trimethylbicyclo[3.1.1]hept-3-en-2-ol $$

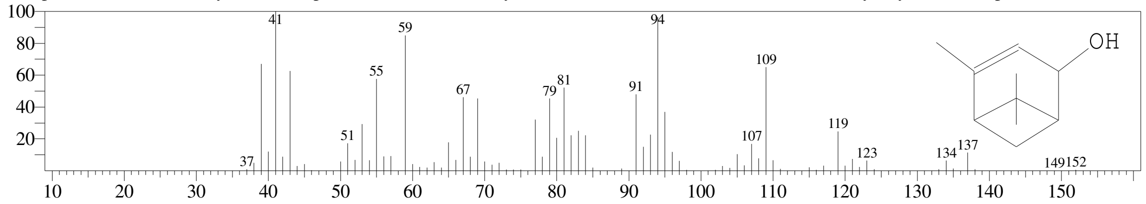

Hit#:2 Entry:16889 Library:NIST14.lib
SI:93 Formula:C10H16O CAS:18881-04-4 MolWeight:152 RetIndex:1136
CompName:Bicyclo[3.1.1]hept-3-en-2-ol, 4,6,6-trimethyl-, [1S-(1.alpha.,2.beta.,5.alpha.)]- $$ (S)-cis-Verbenol $$ (-)-(Z)-Verbenol $$ Bicyclo(3.1.1)hept-3-

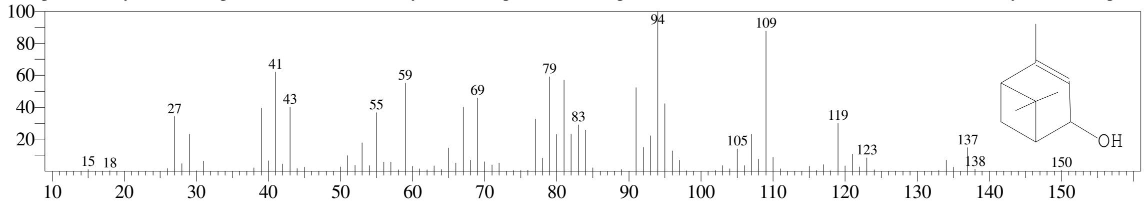

Hit#:3 Entry:16929 Library:NIST14.lib
SI:92 Formula:C10H16O CAS:473-67-6 MolWeight:152 RetIndex:1136
CompName:Verbenol $$ Bicyclo[3.1.1]hept-3-en-2-ol, 4,6,6-trimethyl- $$ Berbenol $$ 2-Pinen-4-ol $$ 4,6,6-Trimethylbicyclo[3.1.1]hept-3-en-2-ol $$

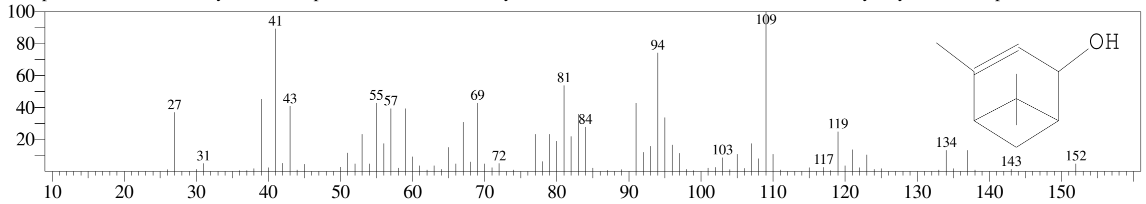

Hit#:4 Entry:16890 Library:NIST14.lib
SI:92 Formula:C10H16O CAS:1845-30-3 MolWeight:152 RetIndex:1136
CompName:cis-Verbenol $$ Bicyclo[3.1.1]hept-3-en-2-ol, 4,6,6-trimethyl-, (1.alpha.,2.beta.,5.alpha.)- $$ Bicyclo(3.1.1)hept-3-en-2-ol, 4,6,6-trimethyl-, (1R

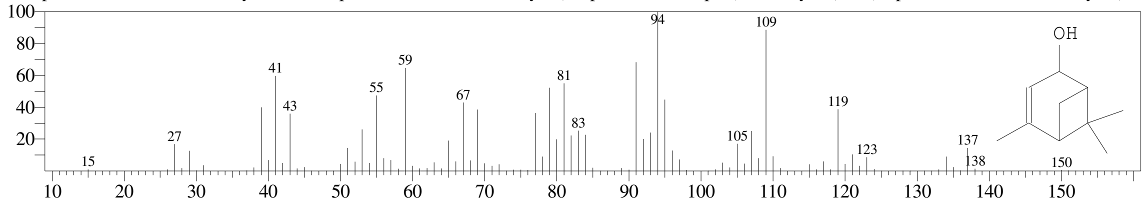

Hit#:5 Entry:9791 Library:NIST14s.lib
SI:91 Formula:C10H16O CAS:1845-30-3 MolWeight:152 RetIndex:1136
CompName:cis-Verbenol $$ Bicyclo[3.1.1]hept-3-en-2-ol, 4,6,6-trimethyl-, (1.alpha.,2.beta.,5.alpha.)- $$ Bicyclo(3.1.1)hept-3-en-2-ol, 4,6,6-trimethyl-, (1R

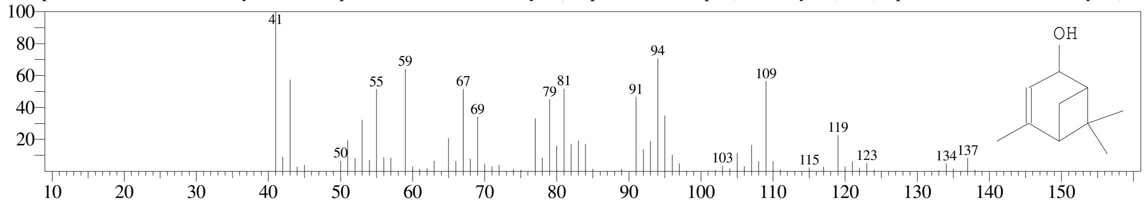

<< Target >>
Line#:10   R.Time:16.350(Scan#:1651) MassPeaks:83
RawMode:Averaged 16.342-16.358(1650-1652) BasePeak:53.00(1025781)
BG Mode:Calc. from Peak Group 1 - Event 1 Scan

Target Spectrum

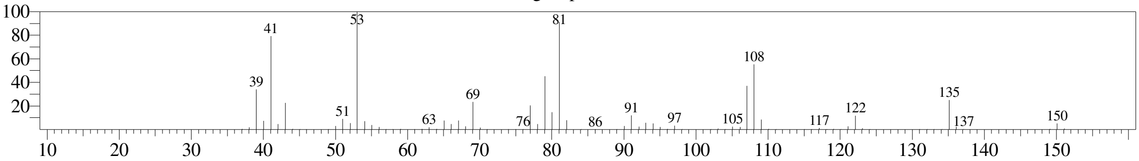

Hit#:1 Entry:9286 Library:NIST14s.lib
SI:93 Formula:C10H14O CAS:30460-92-5 MolWeight:150 RetIndex:1114
CompName:Pinocarvone $$ 2(10)-Pinen-3-one $$ 6,6-Dimethyl-2-methylenebicyclo[3.1.1]heptan-3-one $$ .alpha.-Pinocarvone $$ 3-Nopinenone $$ 2(10)-

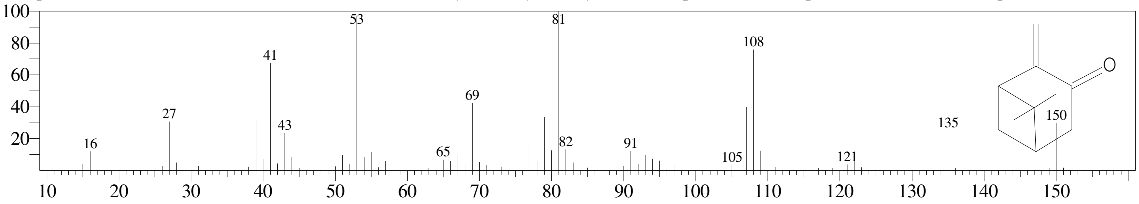

Hit#:2 Entry:9270 Library:NIST14s.lib
SI:91 Formula:C10H14O CAS:30460-92-5 MolWeight:150 RetIndex:1114
CompName:Pinocarvone $$ 2(10)-Pinen-3-one $$ 6,6-Dimethyl-2-methylenebicyclo[3.1.1]heptan-3-one $$ .alpha.-Pinocarvone $$ 3-Nopinenone $$ 2(10)-

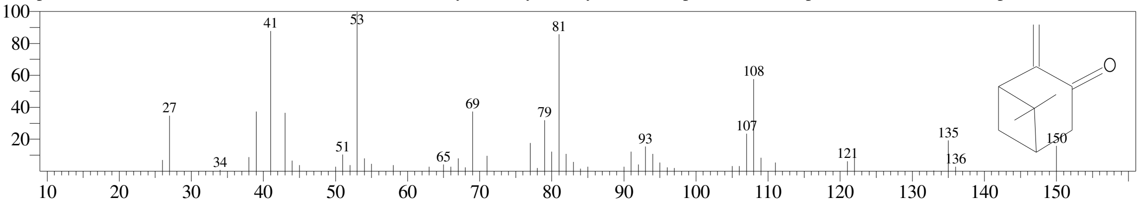

Hit#:3 Entry:9287 Library:NIST14s.lib
SI:91 Formula:C10H14O CAS:30460-92-5 MolWeight:150 RetIndex:1114
CompName:Pinocarvone $$ 2(10)-Pinen-3-one $$ 6,6-Dimethyl-2-methylenebicyclo[3.1.1]heptan-3-one $$ .alpha.-Pinocarvone $$ 3-Nopinenone $$ 2(10)-

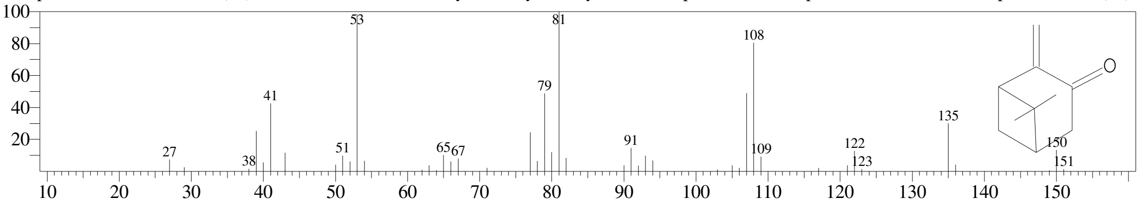

Hit#:4 Entry:15827 Library:NIST14.lib
SI:88 Formula:C10H14O CAS:30460-92-5 MolWeight:150 RetIndex:1114
CompName:Pinocarvone $$ 2(10)-Pinen-3-one $$ 6,6-Dimethyl-2-methylenebicyclo[3.1.1]heptan-3-one $$ .alpha.-Pinocarvone $$ 3-Nopinenone $$ 2(10)-

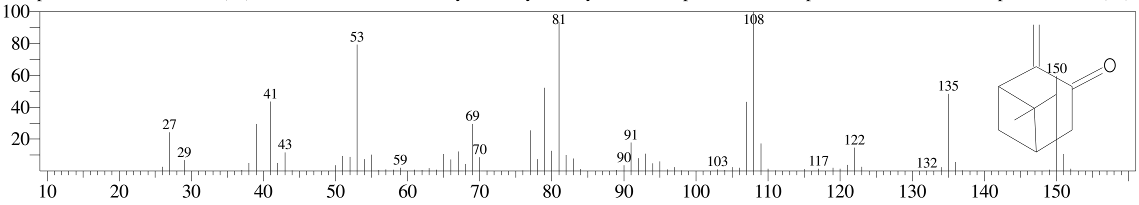

Hit#:5 Entry:18077 Library:NIST14.lib
SI:82 Formula:C10H18O CAS:598-07-2 MolWeight:154 RetIndex:1064
CompName:1,7-Octadien-3-ol, 3,7-dimethyl- $$ .alpha.-Linalool $$ 3,7-Dimethyl-1,7-octadien-3-ol # $$

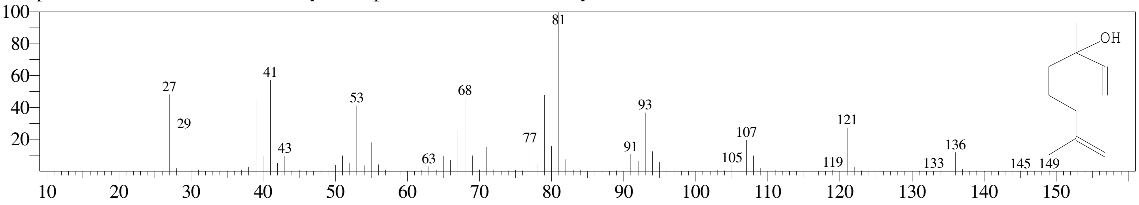

<< Target >>
Line#:11 R.Time:16.842(Scan#:1710) MassPeaks:63
RawMode:Averaged 16.833-16.850(1709-1711) BasePeak:59.05(514007)
BG Mode:Calc. from Peak Group 1 - Event 1 Scan

Target Spectrum

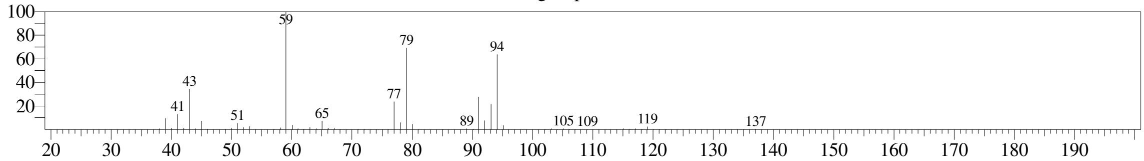

Hit#:1 Entry:16774 Library:NIST14.lib
SI:93 Formula:C10H16O CAS:1686-20-0 MolWeight:152 RetIndex:1125
CompName:p-Mentha-1,5-dien-8-ol $$ 2,4-Cyclohexadiene-1-methanol, .alpha.,.alpha.,4-trimethyl- $$ .alpha.-Phellandren-8-ol $$ 2-(4-Methyl-2,4-cyclohe

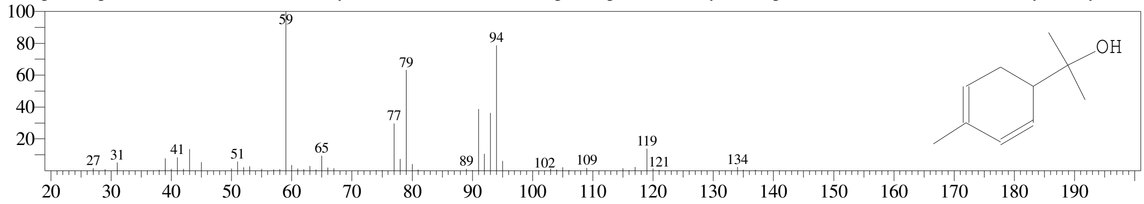

Hit#:2 Entry:9803 Library:NIST14s.lib
SI:84 Formula:C10H16O CAS:1686-20-0 MolWeight:152 RetIndex:1125
CompName:p-Mentha-1,5-dien-8-ol $$ 2,4-Cyclohexadiene-1-methanol, .alpha.,.alpha.,4-trimethyl- $$ .alpha.-Phellandren-8-ol $$ 2-(4-Methyl-2,4-cyclohe

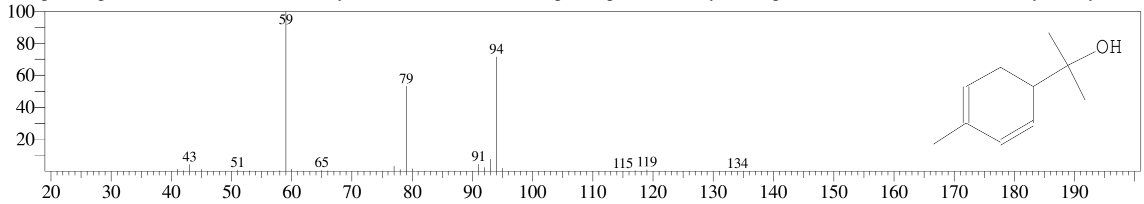

Hit#:3 Entry:56097 Library:NIST14.lib
SI:84 Formula:C12H20O3 CAS:0-00-0 MolWeight:212 RetIndex:1471
CompName:Cyclohexene, 3-acetoxy-4-(1-hydroxy-1-methylethyl)-1-methyl- $$ 6-(1-Hydroxy-1-methylethyl)-3-methyl-2-cyclohexen-1-yl acetate # $$

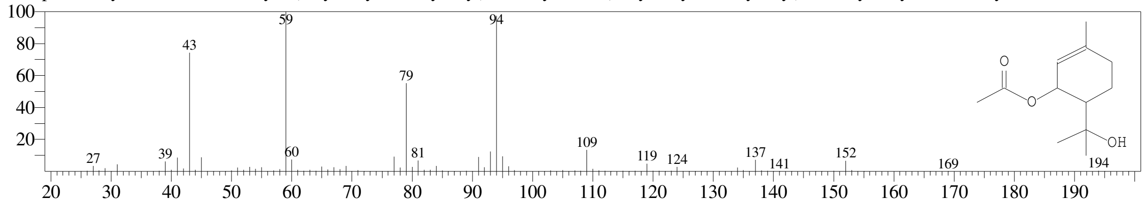

Hit#:4 Entry:16773 Library:NIST14.lib
SI:78 Formula:C10H16O CAS:65293-09-6 MolWeight:152 RetIndex:1120
CompName:p-Mentha-1(7),2-dien-8-ol $$ 2-Cyclohexene-1-methanol, .alpha.,.alpha.-dimethyl-4-methylene- $$ .beta.-Phellandren-8-ol $$ 2-(4-Methylene-2

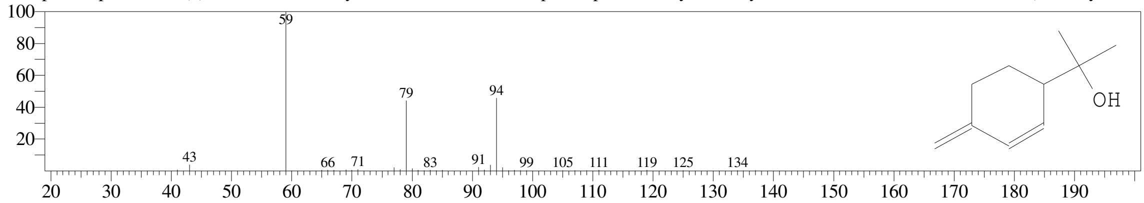

Hit#:5 Entry:43811 Library:NIST14.lib
SI:78 Formula:C10H12O4 CAS:0-00-0 MolWeight:196 RetIndex:1411
CompName:Carbonic acid, 2-methoxyethyl phenyl ester

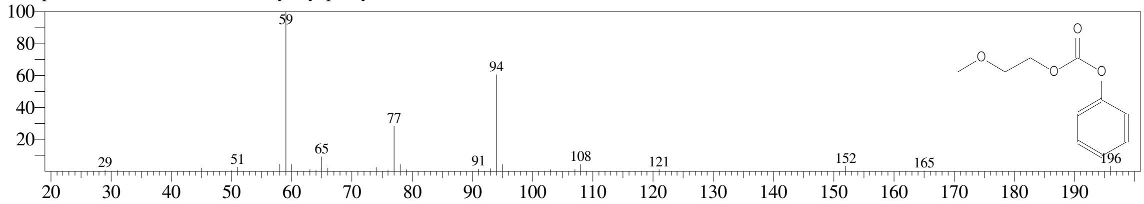

<< Target >>
Line#:12 R.Time:17.892(Scan#:1836) MassPeaks:82
RawMode:Averaged 17.883-17.900(1835-1837) BasePeak:79.05(1166868)
BG Mode:Calc. from Peak Group 1 - Event 1 Scan

Target Spectrum

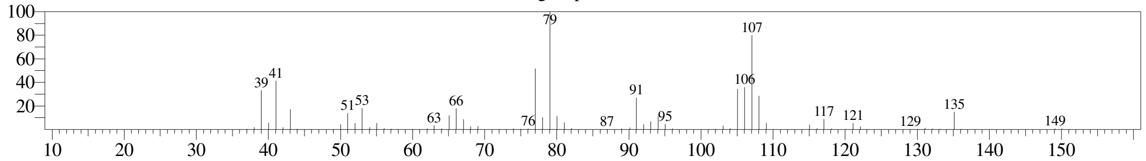

Hit#:1 Entry:9280 Library:NIST14s.lib
SI:95 Formula:C10H14O CAS:564-94-3 MolWeight:150 RetIndex:1136
CompName:Bicyclo[3.1.1]hept-2-ene-2-carboxaldehyde, 6,6-dimethyl- $$ 2-Norpinene-2-carboxaldehyde, 6,6-dimethyl- $$ Myrtenal $$ Benihinal $$ 6,6-D

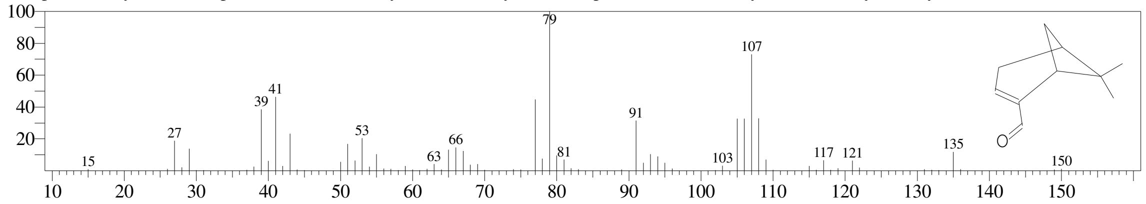

Hit#:2 Entry:15748 Library:NIST14.lib
SI:94 Formula:C10H14O CAS:564-94-3 MolWeight:150 RetIndex:1136
CompName:Bicyclo[3.1.1]hept-2-ene-2-carboxaldehyde, 6,6-dimethyl- $$ 2-Norpinene-2-carboxaldehyde, 6,6-dimethyl- $$ Myrtenal $$ Benihinal $$ 6,6-D

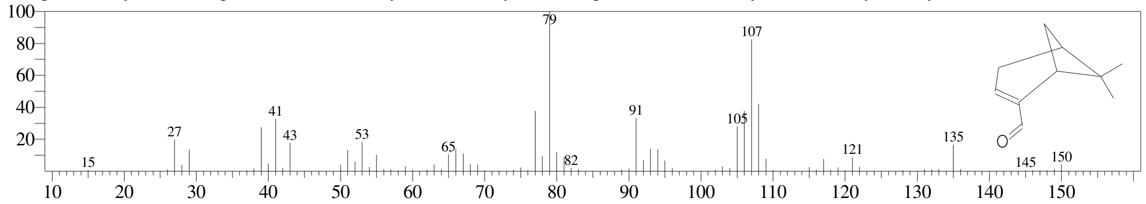

Hit#:3 Entry:9281 Library:NIST14s.lib
SI:94 Formula:C10H14O CAS:564-94-3 MolWeight:150 RetIndex:1136
CompName:Bicyclo[3.1.1]hept-2-ene-2-carboxaldehyde, 6,6-dimethyl- $$ 2-Norpinene-2-carboxaldehyde, 6,6-dimethyl- $$ Myrtenal $$ Benihinal $$ 6,6-D

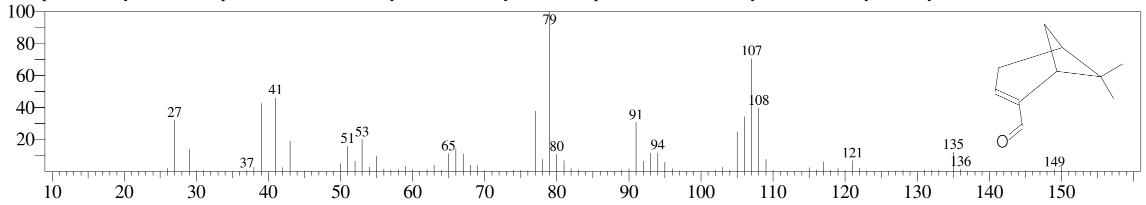

Hit#:4 Entry:15747 Library:NIST14.lib
SI:94 Formula:C10H14O CAS:18486-69-6 MolWeight:150 RetIndex:1136
CompName:(1R)-(-)-Myrtenal $$ 6,6-Dimethylbicyclo[3.1.1]hept-2-ene-2-carbaldehyde # $$

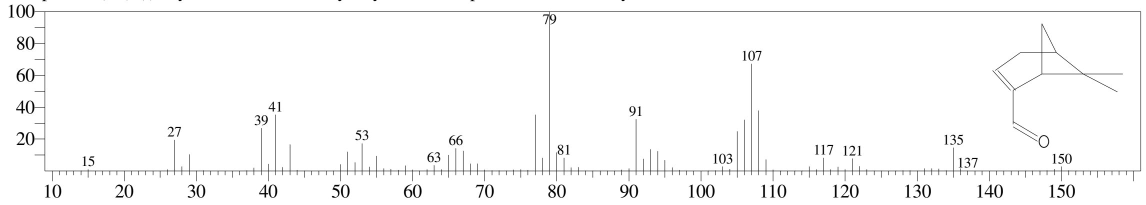

Hit#:5 Entry:9283 Library:NIST14s.lib
SI:91 Formula:C10H14O CAS:564-94-3 MolWeight:150 RetIndex:1136
CompName:Bicyclo[3.1.1]hept-2-ene-2-carboxaldehyde, 6,6-dimethyl- $$ 2-Norpinene-2-carboxaldehyde, 6,6-dimethyl- $$ Myrtenal $$ Benihinal $$ 6,6-D

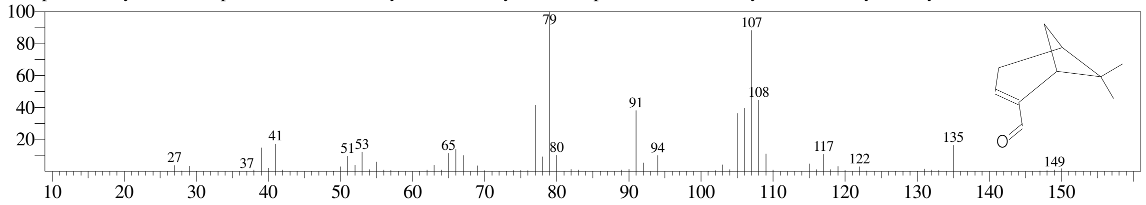

<< Target >>
Line#:13   R.Time:17.992(Scan#:1848) MassPeaks:91
RawMode:Averaged 17.983-18.000(1847-1849) BasePeak:79.05(1349488)
BG Mode:Calc. from Peak Group 1 - Event 1 Scan

Target Spectrum

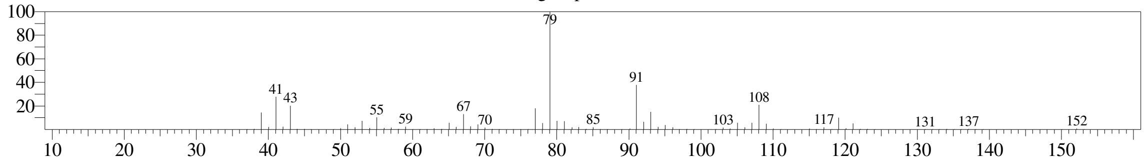

Hit#:1 Entry:9835 Library:NIST14s.lib
SI:95 Formula:C10H16O CAS:515-00-4 MolWeight:152 RetIndex:1191
CompName:Bicyclo[3.1.1]hept-2-ene-2-methanol, 6,6-dimethyl- $$ 2-Pinen-10-ol $$ Myrtenol $$ (6,6-Dimethylbicyclo[3.1.1]hept-2-en-2-yl)methanol # $$

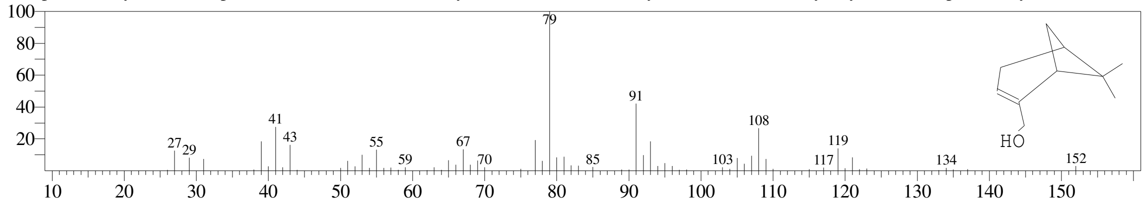

Hit#:2 Entry:16817 Library:NIST14.lib
SI:95 Formula:C10H16O CAS:515-00-4 MolWeight:152 RetIndex:1191
CompName:Bicyclo[3.1.1]hept-2-ene-2-methanol, 6,6-dimethyl- $$ 2-Pinen-10-ol $$ Myrtenol $$ (6,6-Dimethylbicyclo[3.1.1]hept-2-en-2-yl)methanol # $$

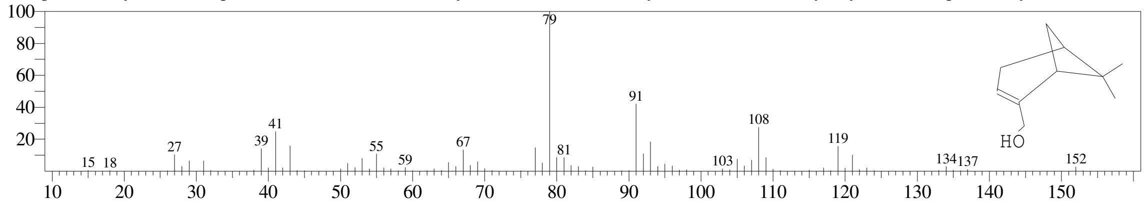

Hit#:3 Entry:9836 Library:NIST14s.lib
SI:92 Formula:C10H16O CAS:515-00-4 MolWeight:152 RetIndex:1191
CompName:Bicyclo[3.1.1]hept-2-ene-2-methanol, 6,6-dimethyl- $$ 2-Pinen-10-ol $$ Myrtenol $$ (6,6-Dimethylbicyclo[3.1.1]hept-2-en-2-yl)methanol # $$

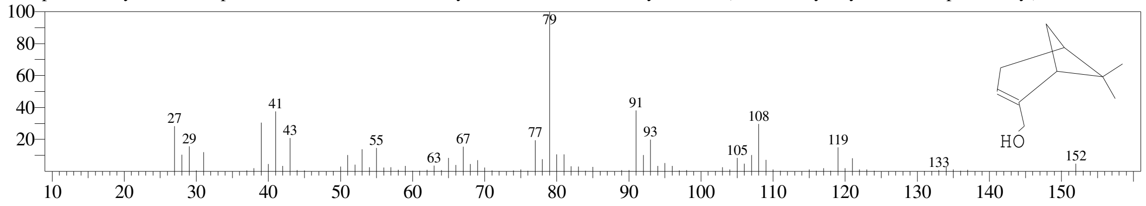

Hit#:4 Entry:16818 Library:NIST14.lib
SI:92 Formula:C10H16O CAS:19894-97-4 MolWeight:152 RetIndex:1191
CompName:(-)-Myrtenol $$ Bicyclo[3.1.1]hept-2-ene-2-methanol, 6,6-dimethyl-, (1R)- $$

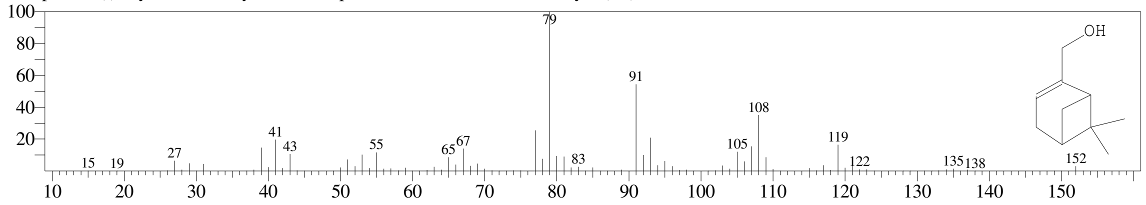

Hit#:5 Entry:9837 Library:NIST14s.lib
SI:89 Formula:C10H16O CAS:515-00-4 MolWeight:152 RetIndex:1191
CompName:Bicyclo[3.1.1]hept-2-ene-2-methanol, 6,6-dimethyl- $$ 2-Pinen-10-ol $$ Myrtenol $$ (6,6-Dimethylbicyclo[3.1.1]hept-2-en-2-yl)methanol # $$

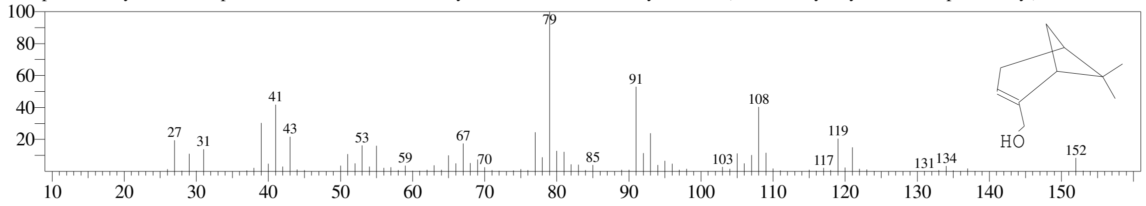

<< Target >>
Line#:14   R.Time:18.408(Scan#:1898) MassPeaks:85
RawMode:Averaged 18.400-18.417(1897-1899) BasePeak:107.10(441599)
BG Mode:Calc. from Peak Group 1 - Event 1 Scan

Target Spectrum

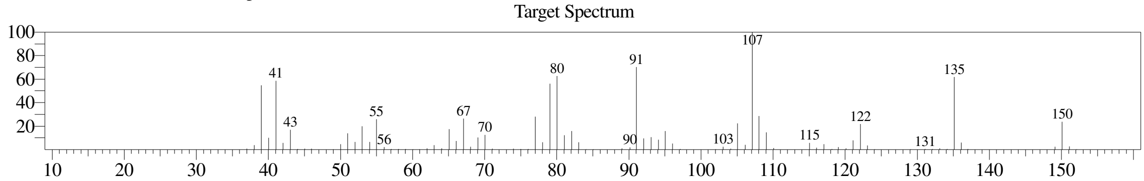

Hit#:1 Entry:9316 Library:NIST14s.lib
SI:95 Formula:C10H14O CAS:80-57-9 MolWeight:150 RetIndex:1119
CompName:Bicyclo[3.1.1]hept-3-en-2-one, 4,6,6-trimethyl- $$ 2-Pinen-4-one $$ Berbenone $$ Verbenone $$ 4,6,6-Trimethylbicyclo[3.1.1]hept-3-en-2-one

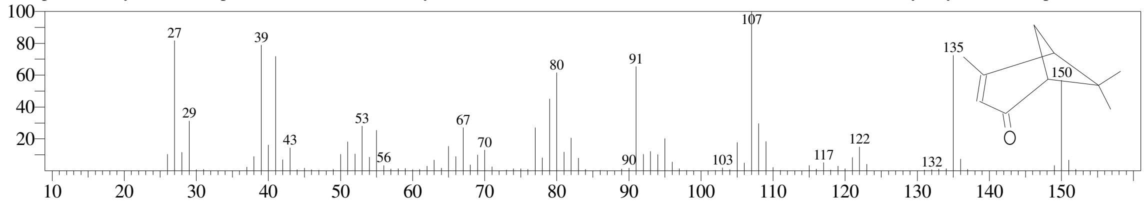

Hit#:2 Entry:9327 Library:NIST14s.lib
SI:94 Formula:C10H14O CAS:80-57-9 MolWeight:150 RetIndex:1119
CompName:Bicyclo[3.1.1]hept-3-en-2-one, 4,6,6-trimethyl- $$ 2-Pinen-4-one $$ Berbenone $$ Verbenone $$ 4,6,6-Trimethylbicyclo[3.1.1]hept-3-en-2-one

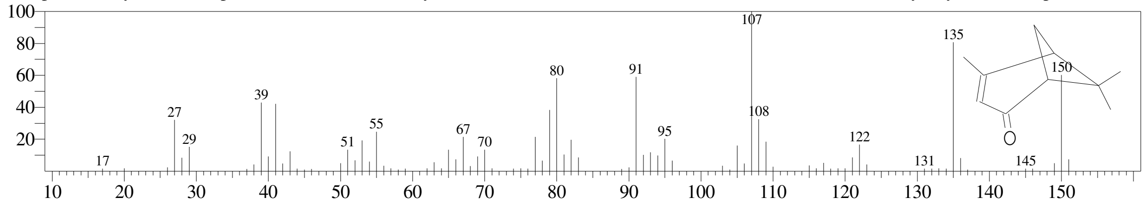

Hit#:3 Entry:15818 Library:NIST14.lib
SI:94 Formula:C10H14O CAS:80-57-9 MolWeight:150 RetIndex:1119
CompName:Bicyclo[3.1.1]hept-3-en-2-one, 4,6,6-trimethyl- $$ 2-Pinen-4-one $$ Berbenone $$ Verbenone $$ 4,6,6-Trimethylbicyclo[3.1.1]hept-3-en-2-one

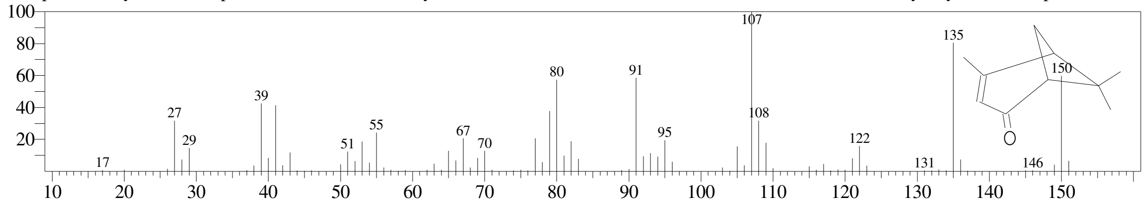

Hit#:4 Entry:9325 Library:NIST14s.lib
SI:94 Formula:C10H14O CAS:80-57-9 MolWeight:150 RetIndex:1119
CompName:Bicyclo[3.1.1]hept-3-en-2-one, 4,6,6-trimethyl- $$ 2-Pinen-4-one $$ Berbenone $$ Verbenone $$ 4,6,6-Trimethylbicyclo[3.1.1]hept-3-en-2-one

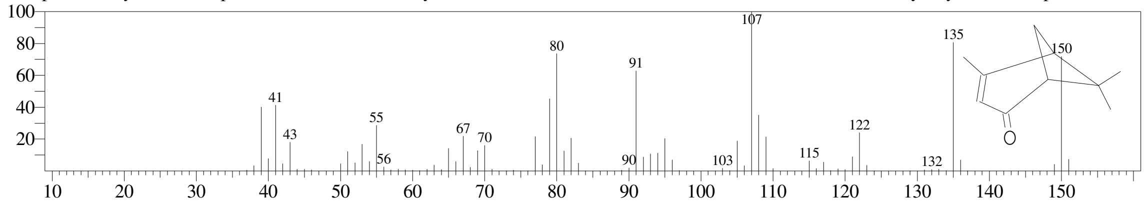

Hit#:5 Entry:15817 Library:NIST14.lib
SI:94 Formula:C10H14O CAS:1196-01-6 MolWeight:150 RetIndex:1119
CompName:Bicyclo[3.1.1]hept-3-en-2-one, 4,6,6-trimethyl-, (1S)- $$ Levoverbenone $$ l-Verbenone $$ Verbenone, (L)- $$ 2-Pinen-4-one, (1S,5S)-(-)- $$

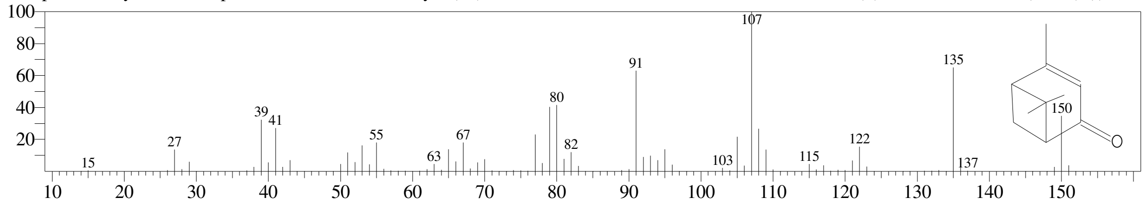

<< Target >>
Line#:15 R.Time:24.092(Scan#:2580) MassPeaks:80
RawMode:Averaged 24.083-24.100(2579-2581) BasePeak:121.10(228319)
BG Mode:Calc. from Peak Group 1 - Event 1 Scan

Target Spectrum

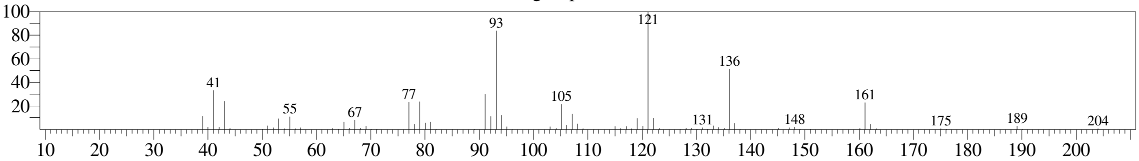

Hit#:1 Entry:19162 Library:NIST14s.lib
SI:94 Formula:C15H24 CAS:20307-84-0 MolWeight:204 RetIndex:1377
CompName:Cyclohexene, 4-ethenyl-4-methyl-3-(1-methylethenyl)-1-(1-methylethyl)-, (3R-trans)- $$ p-Menth-3-ene, 2-isopropenyl-1-vinyl-, (1S,2R)-(-)- $$

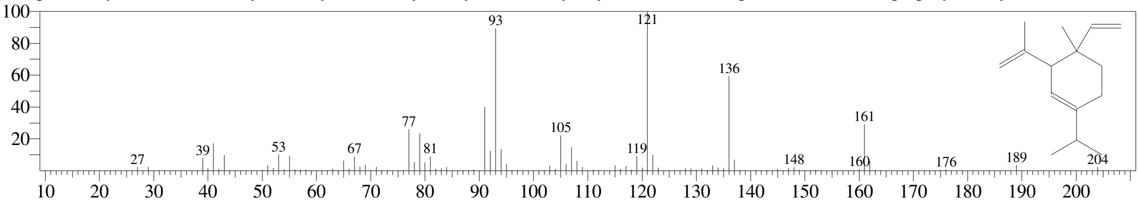

Hit#:2 Entry:49982 Library:NIST14.lib
SI:94 Formula:C15H24 CAS:20307-84-0 MolWeight:204 RetIndex:1377
CompName:Cyclohexene, 4-ethenyl-4-methyl-3-(1-methylethenyl)-1-(1-methylethyl)-, (3R-trans)- $$ p-Menth-3-ene, 2-isopropenyl-1-vinyl-, (1S,2R)-(-)- $$

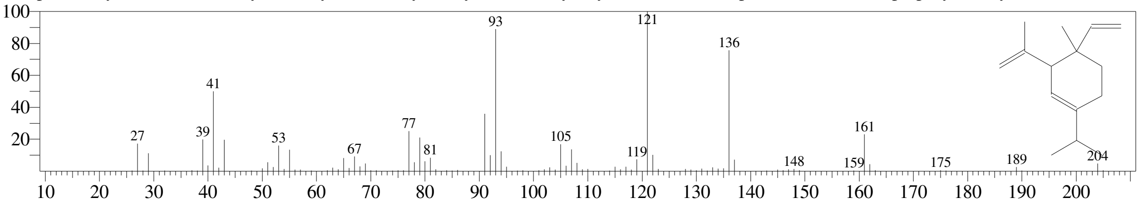

Hit#:3 Entry:19160 Library:NIST14s.lib
SI:91 Formula:C15H24 CAS:20307-84-0 MolWeight:204 RetIndex:1377
CompName:Cyclohexene, 4-ethenyl-4-methyl-3-(1-methylethenyl)-1-(1-methylethyl)-, (3R-trans)- $$ p-Menth-3-ene, 2-isopropenyl-1-vinyl-, (1S,2R)-(-)- $$

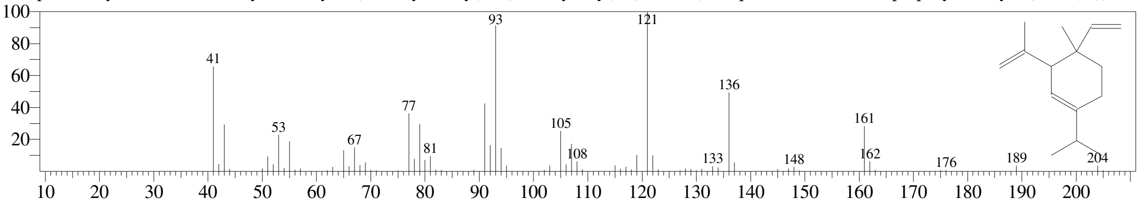

Hit#:4 Entry:6862 Library:NIST14s.lib
SI:91 Formula:C10H16 CAS:99-86-5 MolWeight:136 RetIndex:998
CompName:1,3-Cyclohexadiene, 1-methyl-4-(1-methylethyl)- $$ .alpha.-Terpinene $$ .alpha.-Terpinen $$ p-Mentha-1,3-diene $$ Terpilene $$ 1-Isopropyl-4

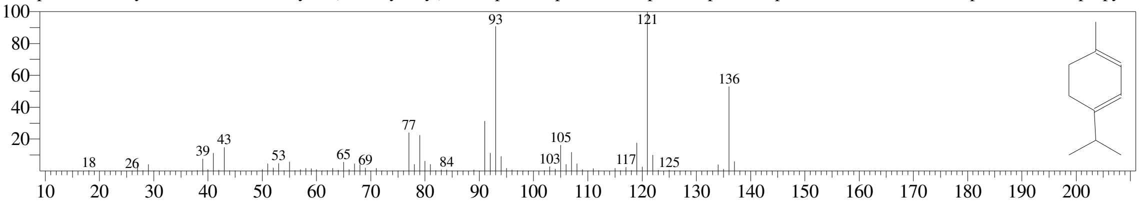

Hit#:5 Entry:10034 Library:NIST14.lib
SI:90 Formula:C10H16 CAS:554-61-0 MolWeight:136 RetIndex:948
CompName:2-Carene $$ Bicyclo[4.1.0]hept-2-ene, 3,7,7-trimethyl- $$ .delta.-2-Carene $$ (.+/-.)-2-Carene $$ 3,7,7-Trimethylbicyclo[4.1.0]hept-2-ene $$

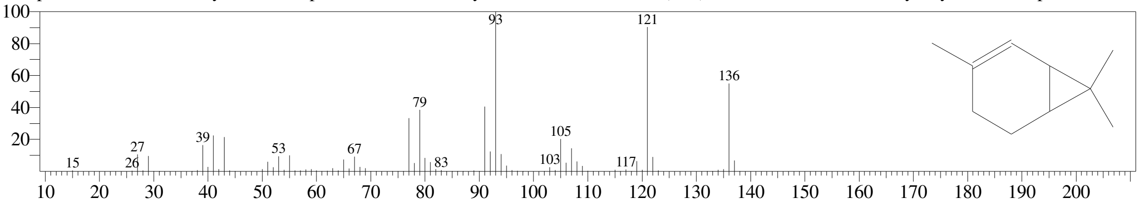

<< Target >>
Line#:16 R.Time:30.183(Scan#:3311) MassPeaks:97
RawMode:Averaged 30.175-30.192(3310-3312) BasePeak:161.10(475541)
BG Mode:Calc. from Peak Group 1 - Event 1 Scan

Target Spectrum

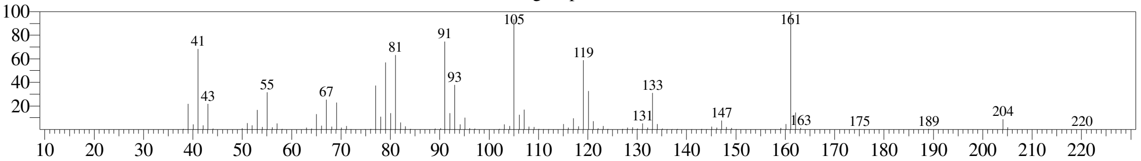

Hit#:1 Entry:50023 Library:NIST14.lib
SI:94 Formula:C15H24 CAS:23986-74-5 MolWeight:204 RetIndex:1515
CompName:Germacrene D $$ (S,1Z,6Z)-8-Isopropyl-1-methyl-5-methylenecyclodeca-1,6-diene $$ D-Germacrene $$ 1(10),4(14),5-Germacratriene $$ (-)-G

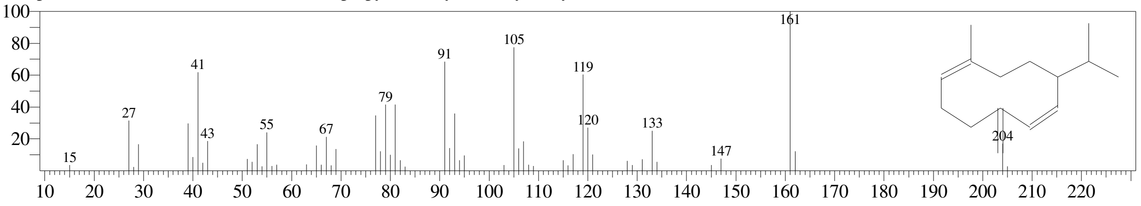

Hit#:2 Entry:50017 Library:NIST14.lib
SI:91 Formula:C15H24 CAS:317819-80-0 MolWeight:204 RetIndex:0
CompName:(S,1Z,6Z)-8-Isopropyl-1-methyl-5-methylenecyclodeca-1,6-diene $$ 1,6-Cyclodecadiene, 1-methyl-5-methylene-8-(1-methylethyl)-, (1Z,6Z,8S)-

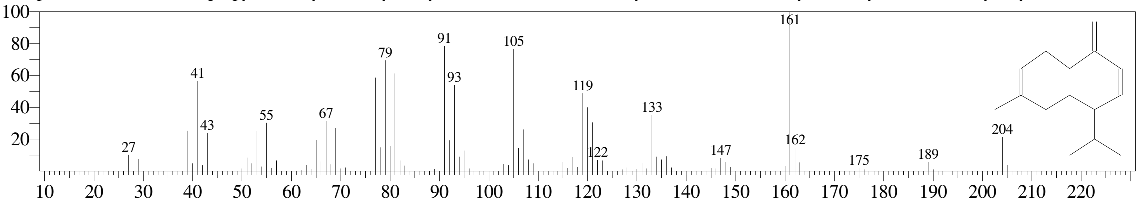

Hit#:3 Entry:49975 Library:NIST14.lib
SI:91 Formula:C15H24 CAS:0-00-0 MolWeight:204 RetIndex:1216
CompName:.beta.-ylangene

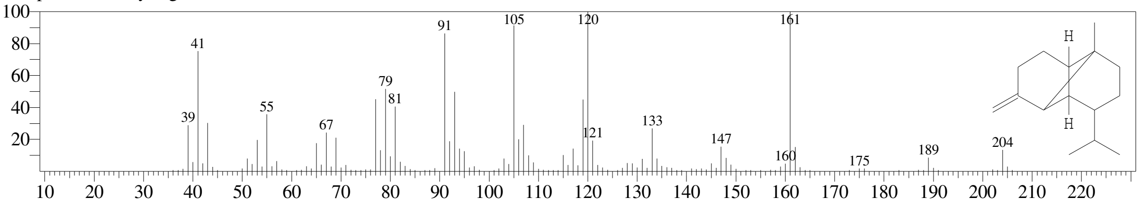

Hit#:4 Entry:50021 Library:NIST14.lib
SI:91 Formula:C15H24 CAS:0-00-0 MolWeight:204 RetIndex:1216
CompName:.beta.-copaene

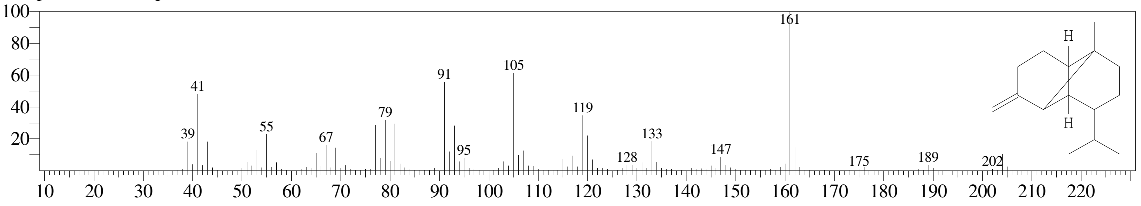

Hit#:5 Entry:19185 Library:NIST14s.lib
SI:91 Formula:C15H24 CAS:23986-74-5 MolWeight:204 RetIndex:1515
CompName:Germacrene D $$ (S,1Z,6Z)-8-Isopropyl-1-methyl-5-methylenecyclodeca-1,6-diene $$ D-Germacrene $$ 1(10),4(14),5-Germacratriene $$ (-)-G

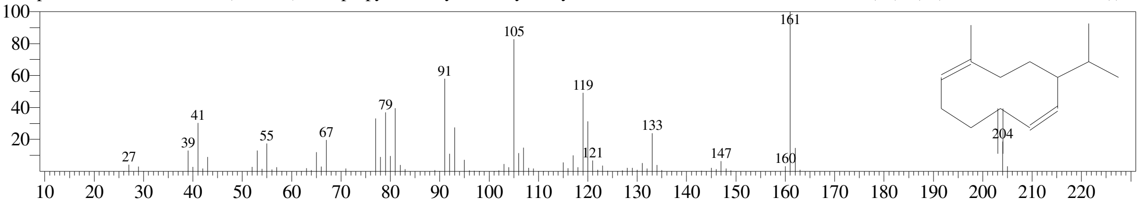

<< Target >>
Line#:17   R.Time:34.050(Scan#:3775) MassPeaks:147
RawMode:Averaged 34.042-34.058(3774-3776) BasePeak:43.00(1138335)
BG Mode:Calc. from Peak Group 1 - Event 1 Scan

Target Spectrum

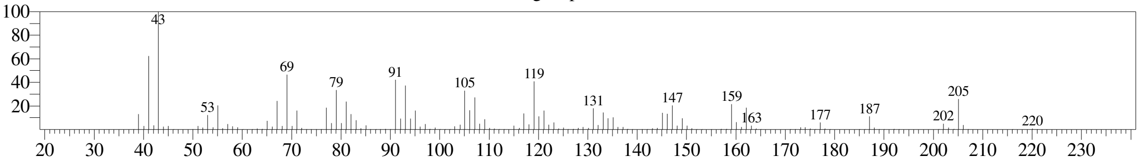

Hit#:1 Entry:62423 Library:NIST14.lib
SI:95 Formula:C15H24O CAS:77171-55-2 MolWeight:220 RetIndex:1536
CompName:(-)-Spathulenol $$ (1aS,4aS,7R,7aS,7bS)-1,1,7-Trimethyl-4-methylenedecahydro-1H-cyclopropa[e]azulen-7-ol $$ 1H-Cycloprop[e]azulen-7-ol,

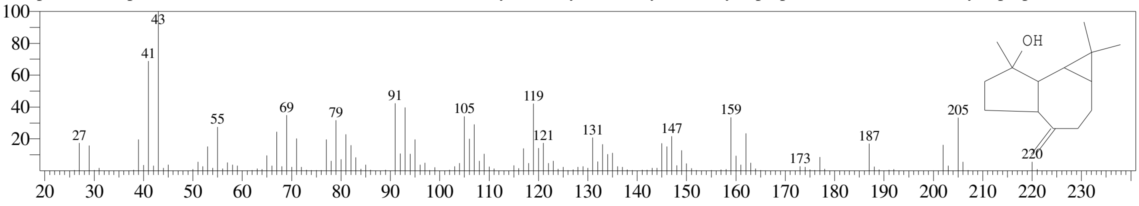

Hit#:2 Entry:62427 Library:NIST14.lib
SI:89 Formula:C15H24O CAS:6750-60-3 MolWeight:220 RetIndex:1536
CompName:1H-Cycloprop[e]azulen-7-ol, decahydro-1,1,7-trimethyl-4-methylene-, [1ar-(1a.alpha.,4a.alpha.,7.beta.,7a.beta.,7b.alpha.)]- $$ (1aR,4aR,7S,7aR

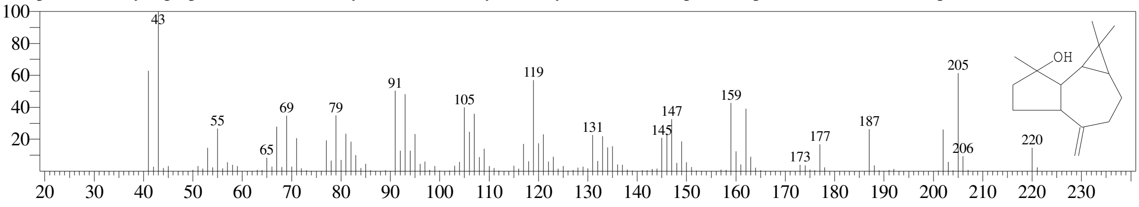

Hit#:3 Entry:21336 Library:NIST14s.lib
SI:89 Formula:C15H24O CAS:6750-60-3 MolWeight:220 RetIndex:1536
CompName:1H-Cycloprop[e]azulen-7-ol, decahydro-1,1,7-trimethyl-4-methylene-, [1ar-(1a.alpha.,4a.alpha.,7.beta.,7a.beta.,7b.alpha.)]- $$ (1aR,4aR,7S,7aR

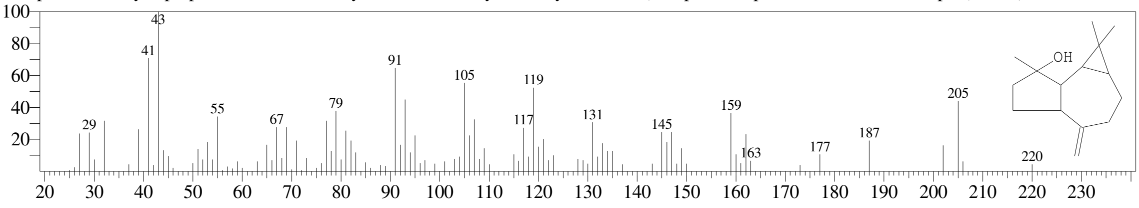

Hit#:4 Entry:77155 Library:NIST14.lib
SI:85 Formula:C15H26O2 CAS:1212211-43-2 MolWeight:238 RetIndex:0
CompName:1,1,4,7-Tetramethyldecahydro-1H-cyclopropa[e]azulene-4,7-diol $$ Aromadendrane-4,10-diol $$

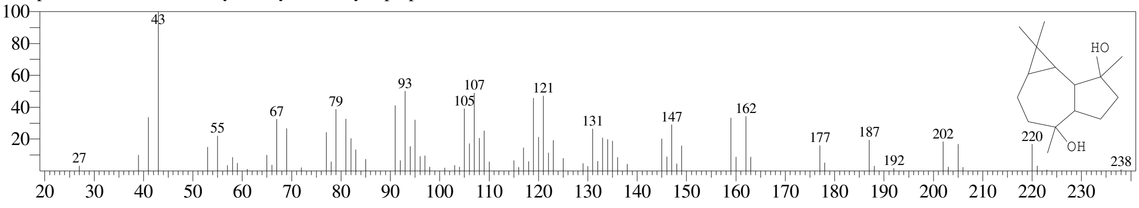

Hit#:5 Entry:62494 Library:NIST14.lib
SI:84 Formula:C15H24O CAS:88395-46-4 MolWeight:220 RetIndex:0
CompName:Isospathulenol $$ (1aR,7S,7aS,7bR)-1,1,4,7-Tetramethyl-1a,2,3,5,6,7,7a,7b-octahydro-1H-cyclopropa[e]azulen-7-ol $$ 1H-Cycloprop[e]azulen

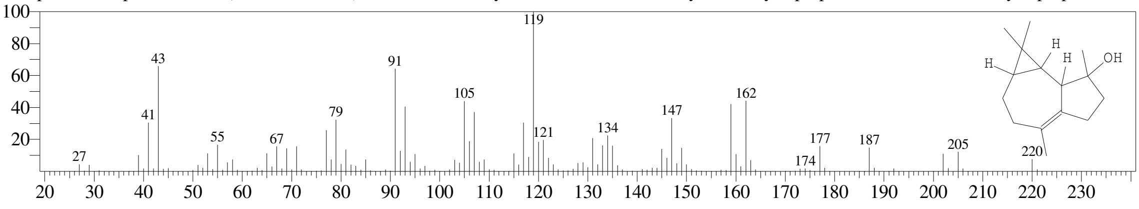

<< Target >>
Line#:18 R.Time:34.158(Scan#:3788) MassPeaks:80
RawMode:Averaged 34.150-34.167(3787-3789) BasePeak:41.05(81668)
BG Mode:Calc. from Peak Group 1 - Event 1 Scan

Target Spectrum

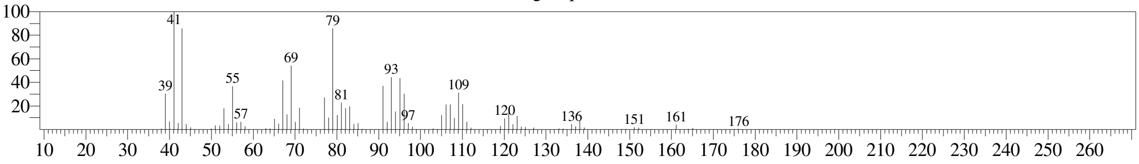

Hit#:1 Entry:62421 Library:NIST14.lib
SI:89 Formula:C15H24O CAS:1139-30-6 MolWeight:220 RetIndex:1507
CompName:Caryophyllene oxide $$ 5-Oxatricyclo[8.2.0.0(4,6)-]dodecane, 4,12,12-trimethyl-9-methylene-, [1R-(1R*,4R*,6R*,10S*)]- $$ 5-Oxatricyclo(8.

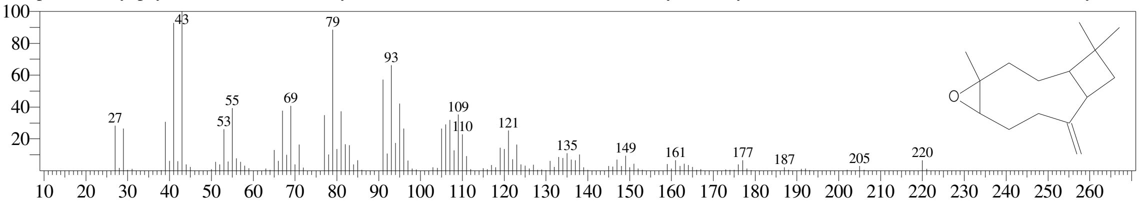

Hit#:2 Entry:21335 Library:NIST14s.lib
SI:88 Formula:C15H24O CAS:1139-30-6 MolWeight:220 RetIndex:1507
CompName:Caryophyllene oxide $$ 5-Oxatricyclo[8.2.0.0(4,6)-]dodecane, 4,12,12-trimethyl-9-methylene-, [1R-(1R*,4R*,6R*,10S*)]- $$ 5-Oxatricyclo(8.

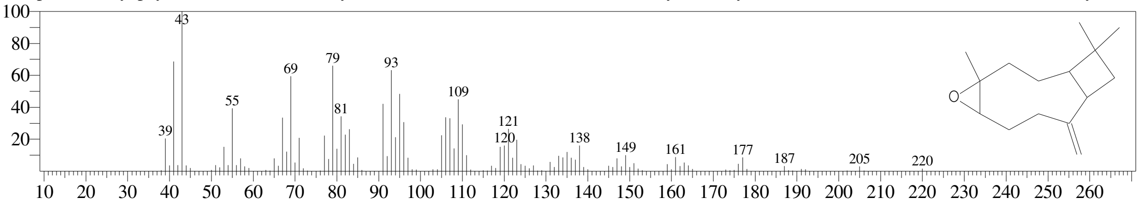

Hit#:3 Entry:17957 Library:NIST14.lib
SI:85 Formula:C10H18O CAS:513-23-5 MolWeight:154 RetIndex:1079
CompName:Bicyclo[3.1.0]hexan-3-ol, 4-methyl-1-(1-methylethyl)- $$ Isothujol $$ 1-Isopropyl-4-methylbicyclo[3.1.0]hexan-3-ol # $$

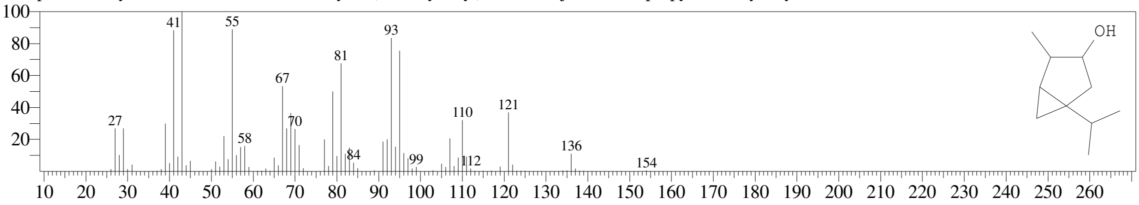

Hit#:4 Entry:97704 Library:NIST14.lib
SI:85 Formula:C19H34 CAS:0-00-0 MolWeight:262 RetIndex:1934
CompName:Z,Z,Z-4,6,9-Nonadecatriene $$ (4Z,6Z,9Z)-4,6,9-Nonadecatriene # $$

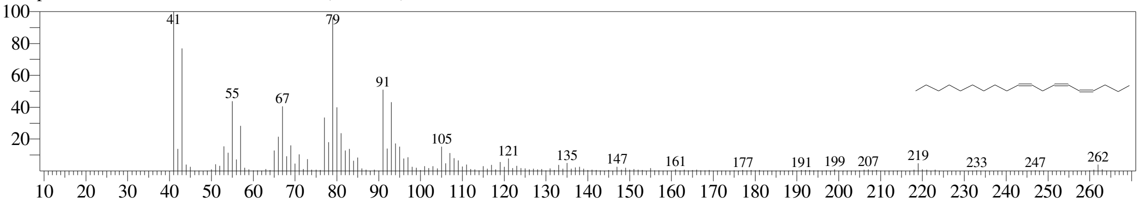

Hit#:5 Entry:17943 Library:NIST14.lib
SI:84 Formula:C10H18O CAS:5944-20-7 MolWeight:154 RetIndex:1228
CompName:3,6-Octadien-1-ol, 3,7-dimethyl-, (Z)- $$ Isogeraniol $$ (3Z)-3,7-Dimethyl-3,6-octadien-1-ol $$ cis-Isogeraniol $$ (Z)-iso-Geraniol $$

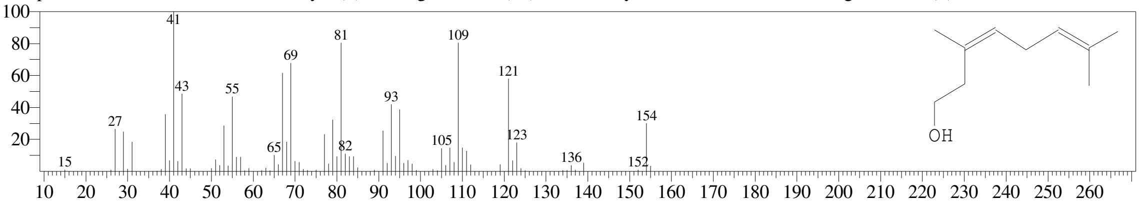

<< Target >>
Line#:19 R.Time:34.550(Scan#:3835) MassPeaks:110
RawMode:Averaged 34.542-34.558(3834-3836) BasePeak:43.00(147363)
BG Mode:Calc. from Peak Group 1 - Event 1 Scan

Target Spectrum

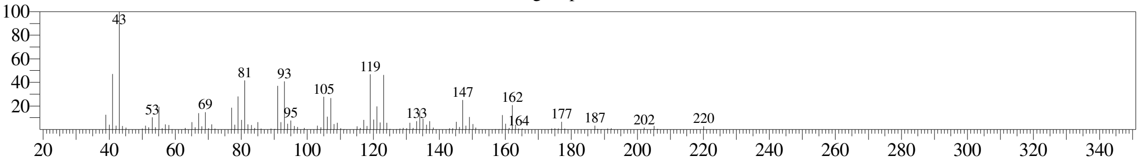

Hit#:1 Entry:62423 Library:NIST14.lib
SI:83 Formula:C15H24O CAS:77171-55-2 MolWeight:220 RetIndex:1536
CompName:(-)-Spathulenol $$ (1aS,4aS,7R,7aS,7bS)-1,1,7-Trimethyl-4-methylenedecahydro-1H-cyclopropa[e]azulen-7-ol $$ 1H-Cycloprop[e]azulen-7-ol,

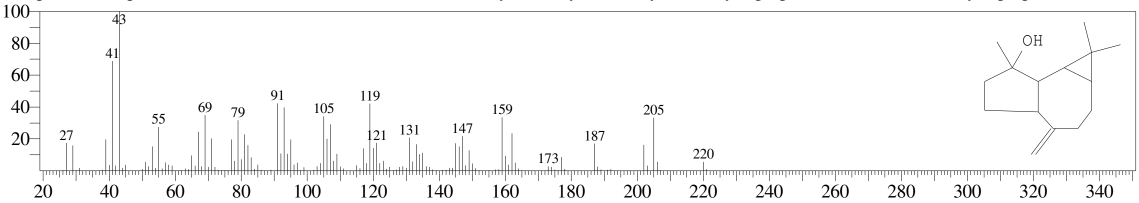

Hit#:2 Entry:172002 Library:NIST14.lib
SI:82 Formula:C22H34O3 CAS:55902-84-6 MolWeight:346 RetIndex:2338
CompName:Kauran-18-al, 17-(acetyloxy)-, (4.beta.)- $$ 18-Oxokauran-17-yl acetate # $$

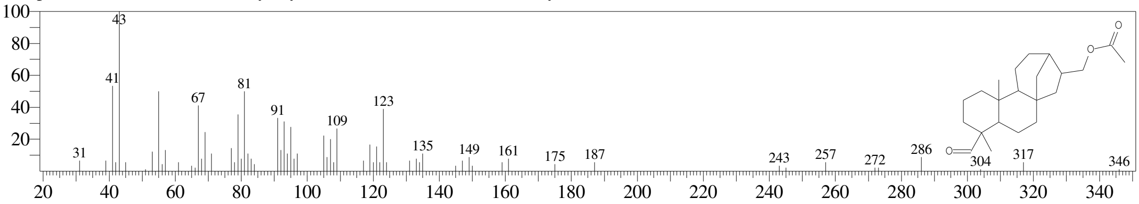

Hit#:3 Entry:62402 Library:NIST14.lib
SI:81 Formula:C15H24O CAS:0-00-0 MolWeight:220 RetIndex:1281
CompName:Isoaromadendrene epoxide $$ 1,3b,6,6-Tetramethyldecahydro-1H-cyclopropa[7,8]azuleno[4,5-b]oxirene # $$

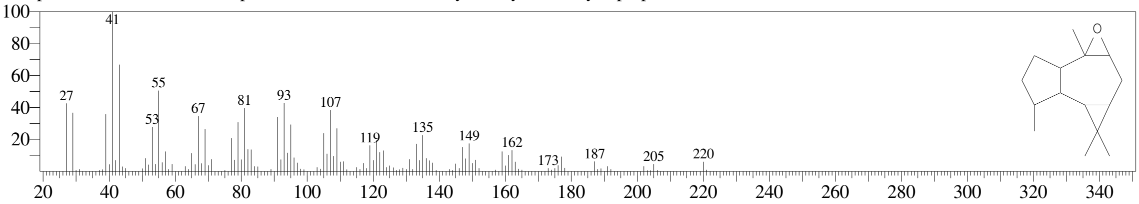

Hit#:4 Entry:62424 Library:NIST14.lib
SI:81 Formula:C15H24O CAS:0-00-0 MolWeight:220 RetIndex:1572
CompName:7-Hydroxyfarnesen

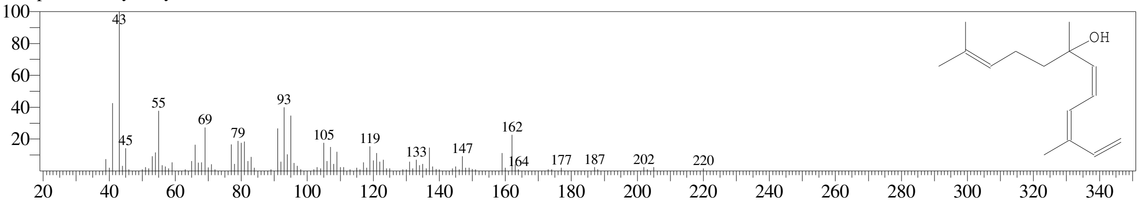

Hit#:5 Entry:60795 Library:NIST14.lib
SI:81 Formula:C15H22O CAS:70038-20-9 MolWeight:218 RetIndex:1452
CompName:7-Oxabicyclo[4.1.0]heptane, 2,2,6-trimethyl-1-(3-methyl-1,3-butadienyl)-5-methylene- $$ 2,2,6-Trimethyl-1-[(1E)-3-methyl-1,3-butadienyl]-5-r

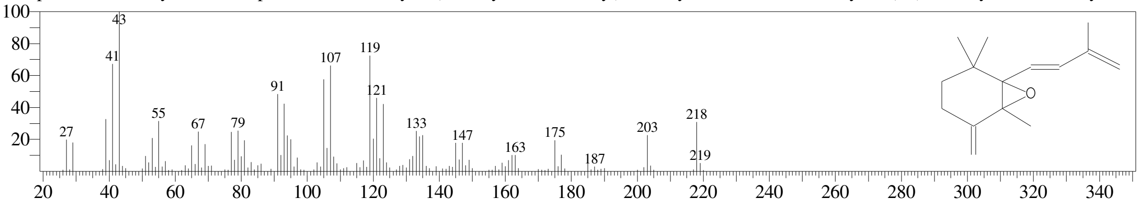

<< Target >>
Line#:20 R.Time:36.017(Scan#:4011) MassPeaks:131
RawMode:Averaged 36.008-36.025(4010-4012) BasePeak:43.00(573776)
BG Mode:Calc. from Peak Group 1 - Event 1 Scan

Target Spectrum

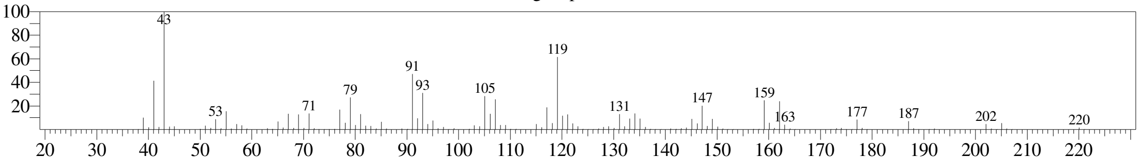

Hit#:1 Entry:62494 Library:NIST14.lib
SI:89 Formula:C15H24O CAS:88395-46-4 MolWeight:220 RetIndex:0
CompName:Isospathulenol $$ (1aR,7S,7aS,7bR)-1,1,4,7-Tetramethyl-1a,2,3,5,6,7,7a,7b-octahydro-1H-cyclopropa[e]azulen-7-ol $$ 1H-Cycloprop[e]azulen

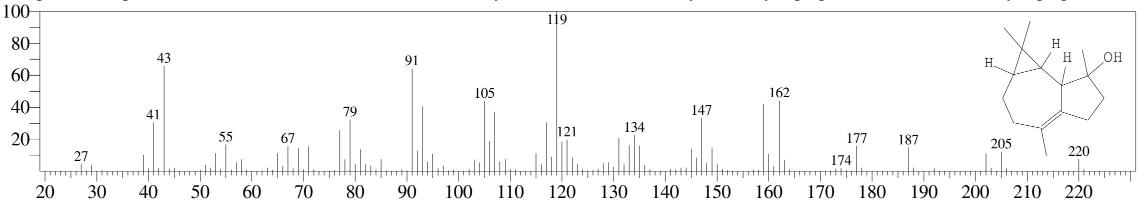

Hit#:2 Entry:62423 Library:NIST14.lib
SI:87 Formula:C15H24O CAS:77171-55-2 MolWeight:220 RetIndex:1536
CompName:(-)-Spathulenol $$ (1aS,4aS,7R,7aS,7bS)-1,1,7-Trimethyl-4-methylenedecahydro-1H-cyclopropa[e]azulen-7-ol $$ 1H-Cycloprop[e]azulen-7-ol,

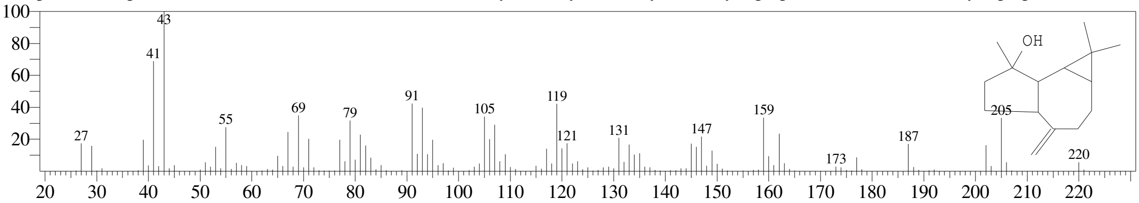

Hit#:3 Entry:62427 Library:NIST14.lib
SI:84 Formula:C15H24O CAS:6750-60-3 MolWeight:220 RetIndex:1536
CompName:1H-Cycloprop[e]azulen-7-ol, decahydro-1,1,7-trimethyl-4-methylene-, [1ar-(1a.alpha.,4a.alpha.,7.beta.,7a.beta.,7b.alpha.)]- $$ (1aR,4aR,7S,7aF

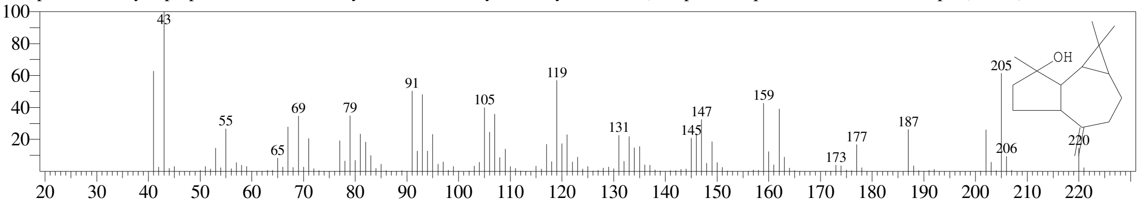

Hit#:4 Entry:21336 Library:NIST14s.lib
SI:83 Formula:C15H24O CAS:6750-60-3 MolWeight:220 RetIndex:1536
CompName:1H-Cycloprop[e]azulen-7-ol, decahydro-1,1,7-trimethyl-4-methylene-, [1ar-(1a.alpha.,4a.alpha.,7.beta.,7a.beta.,7b.alpha.)]- $$ (1aR,4aR,7S,7aF

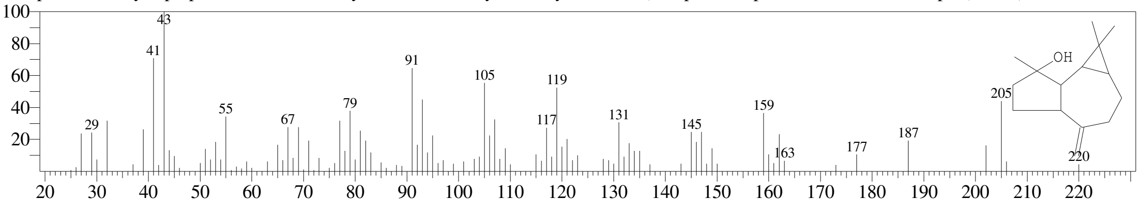

Hit#:5 Entry:62501 Library:NIST14.lib
SI:81 Formula:C15H24O CAS:25570-94-9 MolWeight:220 RetIndex:0
CompName:(1aR,3aR,4R,7R,8aS)-1a,4,9,9-Tetramethyloctahydro-3a,7-methanoazuleno[1,8a-b]oxirene $$ 4H-3a,7-Methano-3H-azuleno[1,8a-b]oxirene, h

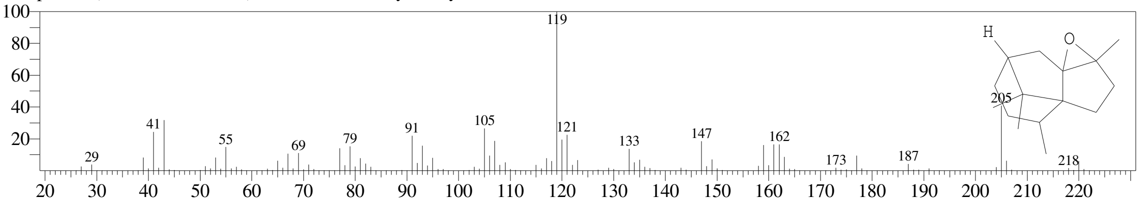

Supplement: Supplementary file 1 [file microorganisms-13-02571-s001.zip › microorganisms-3962249-supplementary.pdf]
